# A deep learning-driven discovery of berberine derivatives as novel antibacterial against multidrug-resistant *Helicobacter pylori*

**DOI:** 10.1038/s41392-024-01895-0

**Published:** 2024-07-08

**Authors:** Xixi Guo, Xiaosa Zhao, Xi Lu, Liping Zhao, Qingxuan Zeng, Fenbei Chen, Zhimeng Zhang, Mengyi Xu, Shijiao Feng, Tianyun Fan, Wei Wei, Xin Zhang, Jing Pang, Xuefu You, Danqing Song, Yanxiang Wang, Jiandong Jiang

**Affiliations:** 1 Institute of Medicinal Biotechnology, Chinese Academy of Medical Sciences and Peking Union Medical College, 100050 Beijing, China; 2 School of Information Science and Technology, Northeast Normal University, Changchun, 130117 China; 3 Department of Pharmacy, Affiliated Hospital of Jining Medical University, Jining Medical University, Jining, 272029 Shandong China; 4 Institute of Health and Medicine, Hefei Comprehensive National Science Center, Hefei, 230601, Anhui, China

**Keywords:** Target identification, Target validation

## Abstract

*Helicobacter pylori* (*H. pylori*) is currently recognized as the primary carcinogenic pathogen associated with gastric tumorigenesis, and its high prevalence and resistance make it difficult to tackle. A graph neural network-based deep learning model, employing different training sets of 13,638 molecules for pre-training and fine-tuning, was aided in predicting and exploring novel molecules against *H. pylori*. A positively predicted novel berberine derivative **8** with 3,13-disubstituted alkene exhibited a potency against all tested drug-susceptible and resistant *H. pylori* strains with minimum inhibitory concentrations (MICs) of 0.25–0.5 μg/mL. Pharmacokinetic studies demonstrated an ideal gastric retention of **8**, with the stomach concentration significantly higher than its MIC at 24 h post dose. Oral administration of **8** and omeprazole (OPZ) showed a comparable gastric bacterial reduction (2.2-log reduction) to the triple-therapy, namely OPZ + amoxicillin (AMX) + clarithromycin (CLA) without obvious disturbance on the intestinal flora. A combination of OPZ, AMX, CLA, and **8** could further decrease the bacteria load (2.8-log reduction). More importantly, the mono-therapy of **8** exhibited comparable eradication to both triple-therapy (OPZ + AMX + CLA) and quadruple-therapy (OPZ + AMX + CLA + bismuth citrate) groups. SecA and BamD, playing a major role in outer membrane protein (OMP) transport and assembling, were identified and verified as the direct targets of **8** by employing the chemoproteomics technique. In summary, by targeting the relatively conserved OMPs transport and assembling system, **8** has the potential to be developed as a novel anti-*H. pylori* candidate, especially for the eradication of drug-resistant strains.

## Introduction

*Helicobacter pylori* (*H. pylori*) is a spiral-shaped gram-negative microaerophilic bacterium which can survive and colonize in the human stomach. *H. pylori* infection is a major risk factor for chronic gastritis, dyspepsia, and peptic ulcers, and is even believed to be a crucial initiating factor leading to gastric cancer and its precancerous lesions.^1–3^ Furthermore, it was the primary carcinogenic pathogen associated with gastric tumorigenesis and classified as a Class I carcinogen by the International Agency for Research on Cancer (IARC) of the World Health Organization (WHO) in 1994.^4,5^ The infection rate of *H. pylori* exceeds 50% worldwide and is even higher in developing countries where sanitation and hygiene practices may be inadequate.^6^

Currently, the first-line eradication regimens for *H. pylori* infection primarily consist of antibiotic-based triple therapy and quadruple therapy, which involve two types of antibacterial drugs, along with a proton pump inhibitor (omeprazole (OPZ)/vonoprazan/lansoprazole, etc.) or/and bismuth agent for synergistic treatment.^7,8^ However, several large clinical trials and meta-analyses have shown that the eradication rate of the standard triple therapy has generally declined to less than 80%.^9^ The main cause is the global increasing drug resistance rate of *H. pylori* to clinically recommended antibiotics, including amoxicillin (AMX), clarithromycin (CLA), metronidazole (MTZ), levofloxacin (LEV), and tetracycline (TC) over the past two decades.^10,11^ The resistance rate of MTZ ranges from 29% to 60% in different WHO regions during 2012 to 2016, whereas the resistance rate of CLA ranges from 21% to 35%.^12^ Additionally, the dual resistance rate of CLA and MTZ is greater than 25%, which makes recurrent infections extremely difficult to be cured.^13,14^ It is worth noting that CLA-resistant *H. pylori* was listed as one of the high-priority category pathogens by WHO, highlighting the urgent need for new antibacterials to combat this issue.^15,16^ Current *H. pylori* eradication regimens are also associated with side effects and gastrointestinal flora imbalances, further emphasizing the need for new treatment options.

Berberine (BBR) has long been used as traditional Chinese medicine for treating gastrointestinal disorders. An increasing number of clinical studies have suggested that the use of BBR in combination with triple therapy could improve the eradication rates of *H. pylori*,^17,18^ with the advantages of proper pharmacokinetic properties, low incidences of adverse reactions, and improved internal environment and pathological repair, etc. It was found that BBR could inhibit the growth, respiration, and sugar metabolism of *H. pylori*.^19^ In addition, BBR can exert anti-*H. pylori* effects by inhibiting the activity of the arylamine *N*-acetyltransferase^20^ and urease.^21,22^ Meanwhile, another protoberberine alkaloid palmatine (PMT) also exhibits antibacterial activity against *H. pylori* both in vitro and in vivo,^23,24^ as well as its potential therapeutic effects on gastritis and peptic ulcer caused by *H. pylori* infection. However, both BBR and PMT only exhibited moderate inhibitory activity against *H. pylori* (minimum inhibitory concentrations, MIC = 16–256 μg/mL).^21,24^ Thus, there is a potential to obtain candidates from BBR or PMT with improved activity, new mechanism of action, and favorable safety profiles.

Deep learning has emerged as a pivotal tool in drug discovery, revolutionizing the field by enabling the rapid and efficient screening of compounds for potential therapeutic applications. Its significance lies in the ability to analyze large datasets with multiple features, extracting intricate patterns that may not be discernible through traditional methods. These models can predict the potency, toxicity, and mode of action of compounds with remarkable accuracy, expediting the identification of promising candidates. Furthermore, deep learning facilitates the exploration of chemical space, allowing for the generation of novel compounds with desired properties.^25^ In the context of antibacterial development, deep learning models have been successfully used in the discoveries of novel antimicrobial agents, such as anti-*Acinetobacter baumannii*^26,27^ and methicillin-resistant *Staphylococcus aureus*^28^ candidates. Therefore, the objective of this study was to establish a dependable deep learning model for the exploration of novel candidates against *H. pylori*.

Firstly, the molecular fingerprint features and molecular graph embeddings were concatenated to form the compound feature vectors through pre-training and fine-tuning. After a rigorous iterative learning process, a deep learning model was constructed based on different training sets of 13,638 molecules. To validate the effectiveness of the deep learning model, a series of novel BBR derivatives were strategically designed, predicted, synthesized, and biologically validated. This endeavor led to the successful prediction of four candidates which exhibited remarkable anti-*H. pylori* activity. Among these predicted candidates, 3-,13-disubstituted analog **8** exhibited the most promising activity against all tested antibacterial-susceptible and resistant *H. pylori* strains, with the MIC values ranging from 0.25–0.5 μg/mL, indicating strong inhibitory activity and a potential different mechanism of action compared to the first-line anti-*H. pylori* antibiotics. Given its potential as a novel anti-*H. pylori* agent, **8** was selected as a representative compound for the further investigation of its potency, safety, and mechanisms of action.

This study showcases a comprehensive and integrative methodology in anti-*H. pylori* agent discovery, leveraging both traditional experimental techniques and cutting-edge machine learning algorithms.

## Results

### A deep learning training set is established for novel anti-*H. pylori* agents exploration

First, the dataset was curated from reputable sources and ensured the diversity in chemical structures and activity levels. A sizable collection of 938 compounds with known anti-*H. pylori* properties was established. This dataset included 801 reported anti-*H. pylori* compounds with structural diversity from ChEMBL database,^29^ as well as 137 self-established BBR derivatives.^30–32^ An MIC value of 16 μg/mL was set as the critical value. The compounds with MICs ≤ 16 μg/mL were defined as ‘active’ (label 1) and MICs > 16 μg/mL as ‘inactive’ (label 0). The proposed deep learning framework firstly represented compounds with molecular graph, and extracted the molecular extended-connectivity fingerprints (ECFP)^33^ which preserve rich functional group information. Feature engineering was performed to extract the ECFP that captured essential functional group information, and leveraged message passing deep neural network to extract properties directly from molecular structure.

**Fig. 1:**
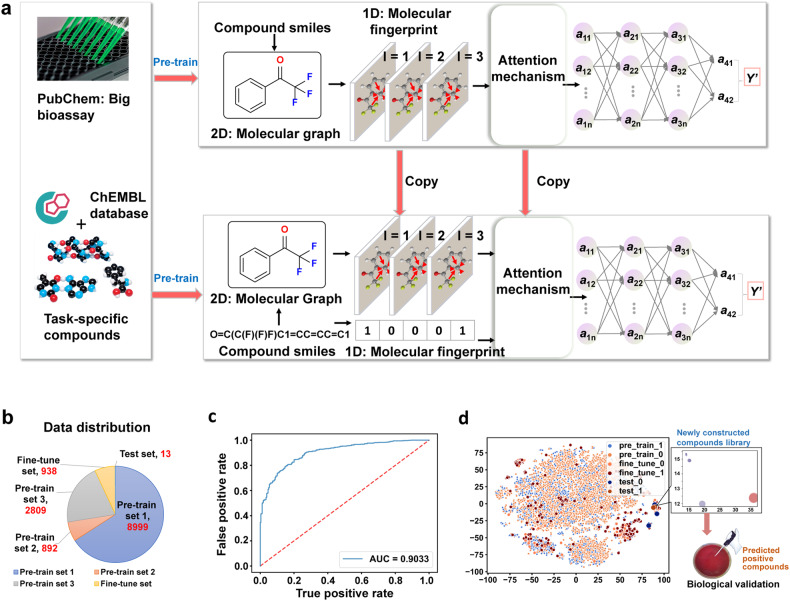
Establishment of the deep learning model. **a** Deep learning-based anti-*H. pylori* compound discovery. SMILES simplified molecular input line entry system. **b** A pie chart for data distributions, including three pre-train sets, a fine-tune set and a test set. **c** ROC-AUC plot evaluating model performance under the ten-fold cross-validation. **d** t-Distributed stochastic neighbor embedding (t-SNE) of all molecules from the pre-training, fine tune, and test set, revealing chemical relationships between these compounds

Since the significant interactions between atomic pairs with topologically distant could also affect the overall molecular properties (Fig. 1a), a deep graph neural network (Attentive FP)^34^ was applied to learn the embeddings of molecular graph, including both local and nonlocal features of the molecular structures. More specifically, every compound was represented with molecular graph, where nodes denoted atoms, and edges denoted bonds (Fig. 1a). By leveraging RDKit and DGL-LifeSci packages, vectors with a length of 39 for nodes and 11 for edges were obtained to represent the chemical properties of atoms and bonds, respectively. Attentive FP was used to translate the molecular graph with node and edge features into a continuous vector, which was the compound representation. Attentive FP iteratively aggregated the features of atoms and bonds with graph attention network (GAT)^35^ in the messaging phases, which allowed an atom to focus on most relevant neighborhoods. Then, it retained and filtered information with a gated recurrent network unit (GRU)^36^ in the readout phases, which allowed the model to capture the implicit effects among distant atoms. After obtaining the molecular graph representation, an attention mechanism to self-adaptively integrate molecular graph representation and ECFP fingerprints was introduced.

Considering that the 938 compounds with known anti-*H. pylori* properties were insufficient for training a successful deep learning model, we utilized the pre-train-then-fine-tuning paradigm,^37^ which pre-trained the deep learning model on large-scale bioassays related to *H. pylori* from PubChem databas,^38^ and fine-tuned the pre-trained deep learning model on the collection of 938 compounds. The pre-train database included 8999, 892, and 2809 compounds (Fig. 1b), respectively. All the above-mentioned training set information was provided as supplementary data sets. In the fine-tune phase, the parameters of the nonlinear multilayer perceptron network (MLP) in the pre-trained deep learning model were initialized and the model was further optimized on the collection of 938 compounds for capturing task-specific patterns. Finally, the molecular fingerprint features and molecular graph embeddings were self-adaptively integrated to form the compound feature vectors and then an MLP layer^39^ was leveraged to predict their activity against *H. pylori*.

The predictive accuracy of the model was assessed through ten-fold cross-validation on the training dataset and external validation on the independent dataset. Cross-validation techniques were applied to validate the robustness and reliability of the model.^40^ The performance of our final model was quantified as follows: the area under the receiver operating characteristic curve (ROC-AUC) attained a value of 0.9033, signifying commendable discriminative capacity; the area under precision-recall curve (AUPR) registered at 0.9615, indicating a robust precision-recall balance. Moreover, the F1-score, a composite metric denoting the harmonious interplay of precision and recall, manifested at 0.8797, attesting to a noteworthy equilibrium between these facets. The model also attained an accuracy rate of 0.8326, representing the proportion of accurately classified instances. Furthermore, the recall, an indicator of the model’s ability to correctly identify actual positives, attained a value of 0.8454, while the precision, signifying the proportion of predicted positives correctly classified, was recorded at 0.9169. These metrics collectively corroborated the model’s effectiveness in addressing specific classification tasks within the ambit of *H. pylori* inhibition.

Thus, this established deep learning model enabled the establishment of a correlation between the structural characteristics of these compounds and their antibacterial activity against *H. pylori*. To validate the effectiveness of this deep learning model, a series of novel BBR derivatives were strategically designed for prediction.

### Four potential anti-*H. pylori* candidates are successfully predicted through this established deep learning model and verified through an activity evaluation in vitro

**Fig. 2:**
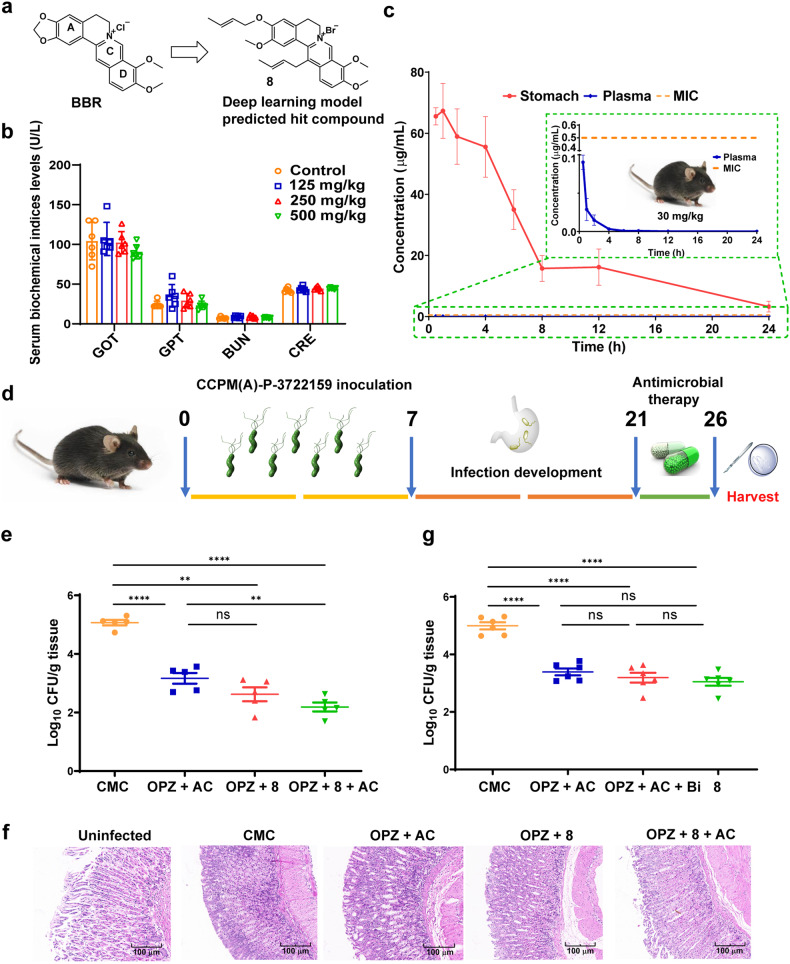
In vivo antibacterial evaluations for compound **8**. **a** Chemical structures of BBR and **8**. **b** Serum biochemical indices of liver and kidney functions for mice in different treatment groups (*n* = 6). **c** Plasma and stomach concentration–time profiles of **8** following a single oral dose of 30 mg/kg (*n* = 4). **d** The schematic diagram of *H. pylori* infection and treatment process in C57BL/6 mice. **e**, **g** The viable counts in the stomach of mice infected with *H. pylori* CCPM(A)-P-3722159 in each group (n = 5) after different treatments. The administration dosage of each treatment component is as follows: OPZ (200 μg/kg); **8** (30 mg/kg); AMX (15 mg/kg); CLA (15 mg/kg); CMC, carboxymethyl cellulose; AC, AMX + CLA; Bi, bismuth citrate (5 mg/kg). **f** Hematoxylin and eosin (H&E) staining of stomach tissues

It is reported that modifications on the D-ring of BBR/PMT (Fig. 2a), such as 9-position mono-substitution, have limited enhancements of anti-*H. pylori* activity.^32^ While modifications were conducted on 13-position of ring C (Fig. 2a), the corresponding derivatives only exhibited moderate anti-*H. pylori* potencies.^41^ Meanwhile, there is scarce literature reporting on the anti-*H. pylori* activity of A-ring modified derivatives, making them highly attractive for novel anti-*H. pylori* drug discovery utilizing deep learning models. Considering the synthetic accessibility, we selectively chose 3-position of the A-ring for modifications with various types of substituents, including chain alkanes, cycloalkanes and substituted phenyls. Thus, a set of 3-substituted novel BBR/PMT derivatives was virtually designed for prediction. Two of them (**5** and **6**) were positively predicted and the rest nine were predicted to be negative (**1**–**4,** **9**–**13**). To verify the accuracy and reliability of the deep learning model employed, all designed compounds were synthesized through an easy-to-operate one-step synthetic procedure as shown in Supplementary Scheme 1, and subsequently subjected to the antibacterial activity evaluation. Simultaneously, two 3,13-disubstituted derivatives (**7** and **8**) were accidentally obtained and identified during the synthesis of **5** and **6**, respectively, with the existence of an excessive α-C containing electrophilic reagent. Compared to previously reported procedures involving more than three steps,^42^ the disubstituted derivatives could be obtained with satisfactory yields ranging from 61–67%. These two compounds were also predicted to be positive (**7**–**8**).

All constructed BBR/PMT derivatives were first evaluated for their activity against six different *H. pylori* strains, including two American Type Culture Collection (ATCC) reference strains of ATCC 43504 and ATCC 700392, and other four clinical isolates, taking BBR, PMT, CLA, AMX, LEV, and MTZ as positive controls. The tested strains included CLA-resistant strains (CCPM(A)-P-3716289 and CCPM(A)-P-3716370), MTZ-resistant strains (ATCC 43504 and CCPM(A)-P-3716289), LEV-resistant strains (CCPM(A)-P-3716289 and CCPM(A)-P-2316370), and an AMX-resistant strain (SS1). The chemical structures of the designed compounds, the deep learning prediction results, and their MIC values against the tested *H. pylori* strains are listed in Table 1. The results demonstrate a notable degree of predictive success, as evidenced by the MIC values. Specifically, the positively predicted compounds (**5**–**8**) exhibited substantially lower MIC values, ranging from 0.25–8 μg/mL. In contrast, for the negatively predicted compounds (**1**–**4**, **9**–**13**), the MIC values went up to a range of 16 to >256 μg/mL. Therefore, compounds **5**, **7**, and **8** with the best antibacterial potencies were selected as representative compounds for further investigation. This approach exemplifies a judicious combination of computational prediction through deep learning models and experimental validation, constituting a powerful strategy for candidate exploration in future anti-*H. pylori* drug development.

**Table 1:**
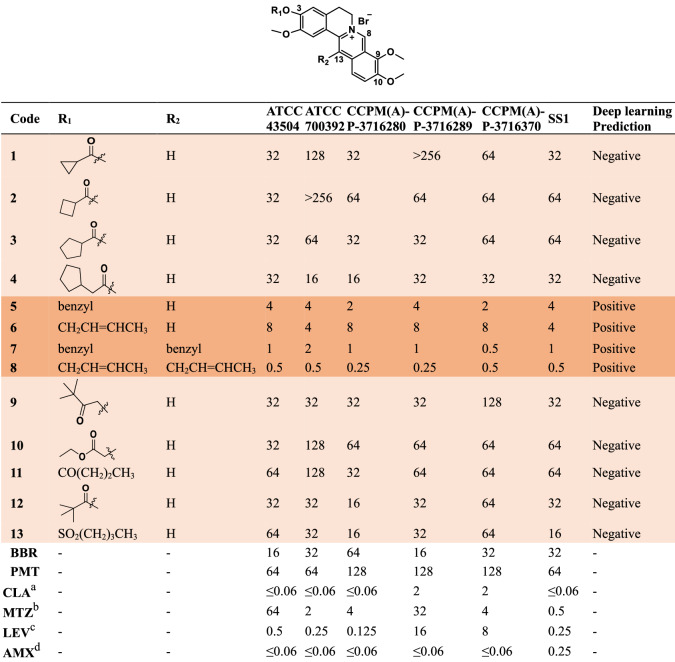
Activities of the designed compounds for prediction against *H. pylori* strains (MIC, μg/mL)

: Positively evaluated compounds

: Negatively evaluated compounds

^a^MIC breakpoint of CLA against *H. pylori*: ≤0.25 μg/mL for susceptible, and >0.25 μg/mL for resistant, according to the European Committee on Antimicrobial Susceptibility Testing (EUCAST)

^b^MIC breakpoint of MTZ against *H. pylori*: ≤8 μg/mL for susceptible, and >8 μg/mL for resistant, according to EUCAST

^c^MIC breakpoint of LEV against *H. pylori*: ≤1 μg/mL for susceptible, and >1 μg/mL for resistant, according to EUCAST

^d^MIC breakpoint of AMX against *H. pylori*: ≤0.125 μg/mL for susceptible, and >0.125 μg/mL for resistant, according to EUCAST

### Representative compound 8 shows a favorable safety and satisfactory pharmacokinetic profile

The effects of predicted hits **5**, **7**, and **8** on cell viability were evaluated using the MTT assay in gastric epithelial cells (GES-1), hepatocellular carcinoma (HepG2), human non-small lung cancer (H460) and human embryonic kidney (293 T) cells. The cell viability was determined after the exposure to varying concentrations of these compounds. As presented in Supplementary Table S1, compound **8** (Fig. 2a) exhibited lower cytotoxicity with the median toxic concentration (TC_50_) values ranging from 50.59 to 57.07 μM, compared to those of **5** (17.68–24.96 μM) and **7** (8.81–12.70 μM). Compound **8** exhibited the best anti-*H. pylori* activity and the lowest cytotoxicity, as well as the most favorable therapeutic index. Therefore, it was selected as a potential candidate for further studies.

The acute oral toxicity test of compound **8** was conducted in Kunming mice. The mice were closely monitored for 14 days, and the medium lethal dose (LD_50_) value of **8** was over 500 mg/kg, which indicated a satisfactory safety profile of **8** for oral administration. Then, the blood samples collected from the above mice were assessed for the biochemical indices of liver and kidney functions. As illustrated in Fig. 2b, **8** did not lead to obviously elevation of glutamic oxalacetic transaminase (GOT), glutamic pyruvic transaminase (GPT), blood urea nitrogen (BUN) or creatine (CRE), indicating no detectable adverse effect of **8** on liver or kidney function.

To explore the pharmacokinetic profile of compound **8**, the stomachs and plasma of C57BL/6 mice were collected and detected at different time points after a single oral dose of 30 mg/kg. As illustrated in Fig. 2c, the gastric concentrations of **8** maintained above its MIC value (0.5 μg/mL) after 24 h (3.25 ± 1.51 μg/g, Supplementary Table S2), indicating an ideal gastric retention that could ensure its anti-*H. pylori* efficacy in vivo. Meanwhile, the maximum concentration (*C*_max_) of **8** in plasma was below 0.1 μg/mL (Supplementary Table S3), and it became undetectable (below the detection limit of 0.001 μg/mL) after 6 h, suggesting a low possibility of systemic side effects. Besides, the acid stability of **8** was also assessed under the pH values of 1.0 and 3.0 (to simulate the acidic environment in gastric acid), at different time points (2, 8, and 24 h). As shown in Supplementary Table S4, the content of **8** was still above 90% after 24 h treatment in the acidic environment. Taken together, the favorable acid stability and pharmacokinetic properties of **8**, including bare absorption to system circulation and long gastrointestinal retention, make it suitable for being developed as an anti-*H. pylori* agent.

### Compound 8 exhibits ideal potencies against drug-susceptible and resistant clinical isolates including multidrug-resistant (MDR) strains

Twenty-seven clinically isolated *H. pylori* strains were employed for further potency evaluation of **8**. As shown in Table 2, compound **8** exhibited a robust activity with an MIC of 0.5 μg/mL against all tested strains (14 CLA-resistant strains, 11 MET-resistant strains, 10 LEV-resistant strains, 2 AMX-resistant strains, and 6 MDR strains, and all the resistant information is highlighted in dark color in Table 2).

**Table 2:**
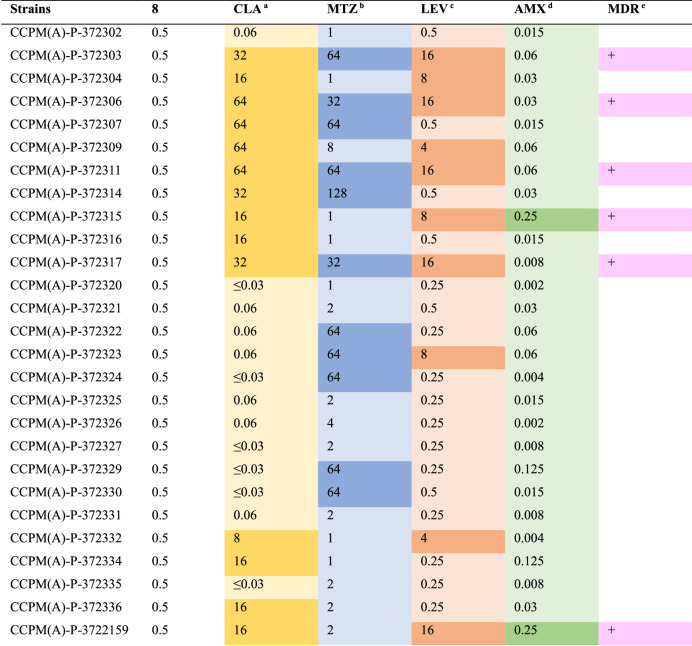
Activities of compound **8** against 27 clinical isolated *H. pylori* strains (MIC, μg/mL)

^a^CLA: MIC breakpoint of CLA for *H. pylori*: ≤0.25 μg/mL for susceptible, and >0.25 μg/mL for resistant, according to EUCAST

^b^MTZ: MIC breakpoint of MTZ for *H. pylori*: ≤8 μg/mL for susceptible, and >8 μg/mL for resistant, according to EUCAST

^c^LEV: MIC breakpoint of LEV for *H. pylori*: ≤1 μg/mL for susceptible, and >1 μg/mL for resistant, according to EUCAST

^d^AMX: MIC breakpoint of AMX for *H. pylori*: ≤0.125 μg/mL for susceptible, and >0.125 μg/mL for resistant, according to EUCAST

^e^MDR, multidrug-resistant, defined as acquired non-susceptibility to at least one agent in three or more antimicrobial categories

Compound **8** was then challenged over a 36-day serial passage assay to determine the rate of potential resistance induction on *H. pylori* ATCC 43504, which is susceptible to CLA and AMX originally. As shown in Supplementary Fig. S1, repeated exposure to sub-MIC level of **8** or AMX did not develop resistance in the tested *H. pylori* strain by serial passage (12 passages). After 12 passages under permanent selective pressure of CLA, the bacteria showed resistance to CLA with the MIC reaching and stabilizing at 4 μg/mL (256-fold of initial MIC).

Checkerboard assay was performed to test the combined effects of **8** and AMX or CLA. As displayed in Supplementary Table S5, when combined with CLA, synergistic effects (fractional inhibitory concentration index, FICI ≤ 0.5) could be observed in 10 out of 25 tested strains (5 out of 9 CLA-resistant strains) with the FICI values of 0.188–0.50. Meanwhile, only additive effect (0.5 < FICI ≤ 4) was observed in the combination of AMX and **8** (Supplementary Table S6).

### Compound 8 shows a promising in vivo activity against the MDR *H. pylori* strain CCPM(A)-P-3722159

The in vivo antibacterial activity of compound **8** was evaluated in the C57BL/6 mouse gastric infection model (Fig. 2d). The mice were first randomly assigned into five groups: an uninfected control group and four infected groups with different treatments, which included a vehicle carboxymethyl cellulose (CMC) control group, dual therapy group (OPZ plus **8** [OPZ + **8**]), triple therapy group (OPZ plus AMX and CLA [OPZ + AC]), and quadruple therapy group (OPZ plus AMX, CLA, and **8** [OPZ + AC + **8**]), respectively. The mice in the infected groups were orally administrated *via* gavage with *H. pylori* CCPM(A)-P-3722159, a mouse-adapted MDR strain (resistant to AMX, CLA, and LEV), every other day for four times. After a two-week colonization period, the different treatments were performed as above for five consecutive days. The therapeutic efficacy was evaluated by comparing the viable bacteria counts in the mouse stomachs. As shown in Fig. 2e, treatment with OPZ + **8** (30 mg/kg) significantly decreased the gastric bacteria load of the infected mice from 1.3 × 10^5^ to 6.5 × 10^2^ CFU/g (2.2-log reduction in comparison to CMC group), which was similar to that of the triple-therapy group (OPZ + AC, 1.8-log reduction in bacterial burden). Remarkably, the quadruple-therapy treatment (OPZ + AC + **8**) further decreased the bacteria load to 2.0 × 10^2^ CFU/g (2.8-log reduction), representing a 99.8% inhibition of stomach colonization compared with CMC group. These results suggest that, with the pretreatment of OPZ, **8** could exert comparable eradicative efficacy to the combination of OPZ, AMX and CLA in vivo, and exhibited improved activity when combined with AMX and CLA, thereby increasing the clearance of the colonized multidrug-resistant *H. pylori*.

Additionally, there was no significant body weight loss after the different treatment, as shown in Supplementary Fig. S2. Histopathological examination of fixed stomach sections revealed that *H. pylori* infection led to a more porous and bloated structure of the gastric gland, the obvious inflammatory infiltration, and the increase of pepsinogen (high pepsinogen usually related to *H. pylori* infection, peptic ulcer, and gastritis) compared with the uninfected tissue (Fig. 2f). The dual, triple, and quadruple-therapy treatments alleviated the gastric inflammation in some degree and decreased the level of pepsinogen, indicating the eradication of the pathogens.

### The diversity of the intestinal flora and the abundance of probiotics are partially restored with the treatment of compound 8

**Fig. 3:**
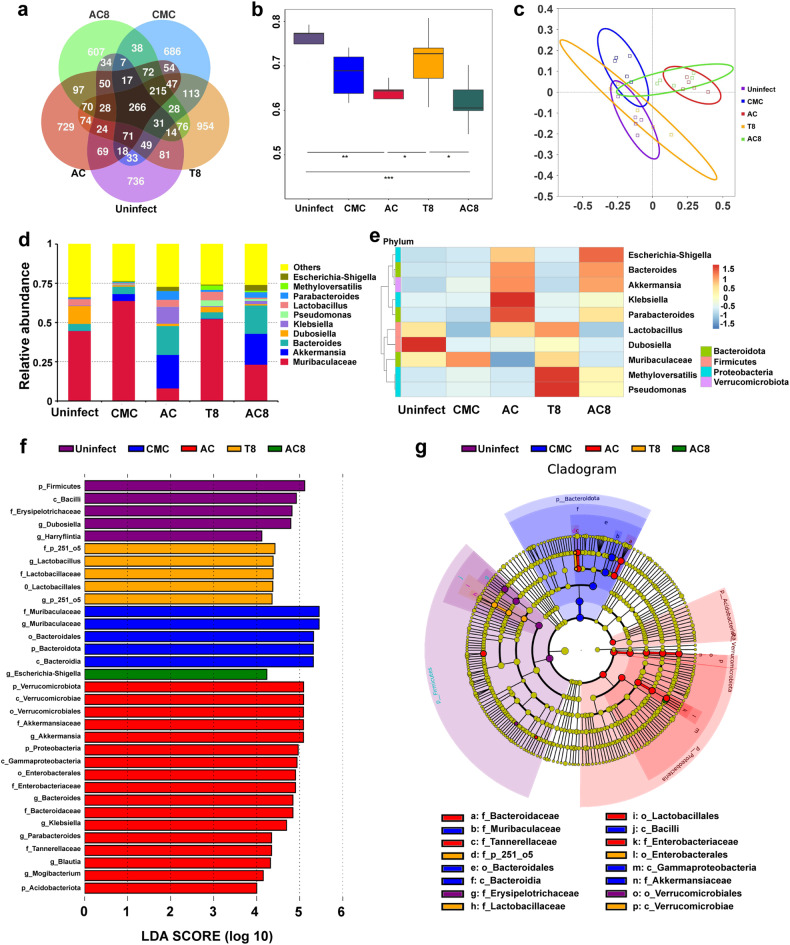
Gut microbiome analysis in different treatment groups (*n* = 5). Uninfect, the uninfected group; CMC, vehicle control group; T8, dual therapy group (OPZ + **8**); AC, triple therapy group (OPZ + AC); AC8, quadruple therapy group (OPZ + AC + **8**). **a** The Venn diagram of microbial characteristic sequences of each treatment group. **b** Alpha diversity analysis on microbiota diversity of each treatment group. **c** Beta diversity of PCoA analysis. **d** A bar plot analysis at the genus level (ten bacterial genera with the highest abundance). **e** A heatmap analysis at the genus level (ten bacterial genera with the highest abundance). **f** LDA value distribution histogram revealed by LEfSe software. When species with LDA Score >4 are statistically different, the length of the histogram (LDA Score) represent the impact size of the different species. **g** Evolutionary branching trees from the inside out in a clade represent the level of phylum, class, order, family, genus

The long-term use of antibiotics often leads to a disturbance of the intestinal flora and a decrease in gut microbiota diversity. To investigate whether **8** affects the gut microbiota, stool samples were collected from each group, and 16S rRNA gene sequencing was employed to analyze the gut microbiota constitution. The Venn graph was used to analyze the characteristic sequence numbers of each group. As shown in Fig. 3a, the largest number of same specific characteristic sequences between **8** treatment group (T8: OPZ + **8**) and the uninfected group were observed, compared with other comparisons. Using alpha diversity (Pieloi_e) analysis (Fig. 3b), the microbiota diversity in the vehicle control group (CMC) and triple therapy group (OPZ + AC) was found to be significantly decreased compared with that in the uninfected group at the genus level. It is worth noting that, the box diagram showed that the diversity of intestinal flora of mice in group T8 was close to that in the healthy group (*p* > 0.05), higher than that in CMC group and group OPZ + AC. Principal coordinate analysis (PCoA) showed that, in comparison to CMC and OPZ + AC groups, the composition of intestinal flora of the T8 group exhibited more similarity to the uninfected group (Fig. 3c).

Next, a bar plot and a heat map analysis at the genus level were performed to show the ten bacterial genera with the highest abundance of each treatment group (Fig. 3d, e). Through the relative abundance analysis at genus level, the intestinal flora disorder was observed in AC group, with the overgrowth of several genera, including *Klebsiella*, *Escherichia-Shigella*, and *Bacteroides*. In contrast to AC group, the microbiota constitution of the dual therapy group (T8) was sustained and the abundance of probiotics, including *Lactobacillus* and *Dubosiella* was partially restored. The bacterial genera with the highest abundance in each mouse was also displayed in Supplementary Fig. S3. In addition, *Bifidobacterium*, another kind of well-known probiotics (not belonging to ten highest abundance), was also significantly enriched in the dual therapy group compared with AC group (Supplementary Fig. S4), confirming that **8** has the tendency to avoid dysbiosis of intestinal flora. To further display the observed differences in the microbiome composition, linear discriminant analysis (LDA) effect size (LEfSe) analysis (Fig. 3f) was performed, and the cladogram was generated based on LEfSe analysis (Fig. 3g). Consistent with the above results, there was a significant increase in the abundance of *Lactobacillus* (LDA (log_10_) > 4.0, *p* < 0.05) in the dual therapy group. These results suggest that **8** might not exert an impact on the diversity of the intestinal flora, and increase the abundance of some probiotics while eradicating *H. pylori*.

To figure out why compound **8** could exhibit anti-*H. pylori* activity without exerting an impact on intestinal microbiota, the antibacterial spectrum of **8** was evaluated. The antibacterial activities of **8** against common gram-positive and negative bacteria were shown in Supplementary Table S7. Compound **8** only exhibited a moderate antibacterial efficacy against *Staphylococcus aureus* ATCC 29213 (MIC value: 8 μg/mL), while being ineffective against all tested gram-negative bacteria. Therefore, the antibacterial spectrum indicates the specific inhibitory effect of compound **8** against *H. pylori*, while exerting minor impact on the intestinal microbiota.

### Mono-therapy of 8 shows a comparable potency compared with both the triple-therapy and the quadruple-therapy

Proton pump inhibitor including OPZ is recommended to take before meals to avoid the over-production of gastric acid, so as to increase the stability of antibiotics. Considering that compound **8** possessed an ideal profile of acid stability, in vivo activity of compound **8** itself was evaluated, without the co-administration of OPZ. As shown in Fig. 2g, the mono-therapy of **8** showed a comparable potency compared with both the triple-therapy (OPZ + AMX + CLA) and the quadruple-therapy (OPZ + AMX + CLA + bismuth citrate). These results indicated that mono treatment of compound **8** may be applied as an alternative therapy of traditional triple or quadruple *H. pylori* eradication regimen.

### Morphologic analysis is carried out on *H. pylori* after the treatment of compound 8

**Fig. 4:**
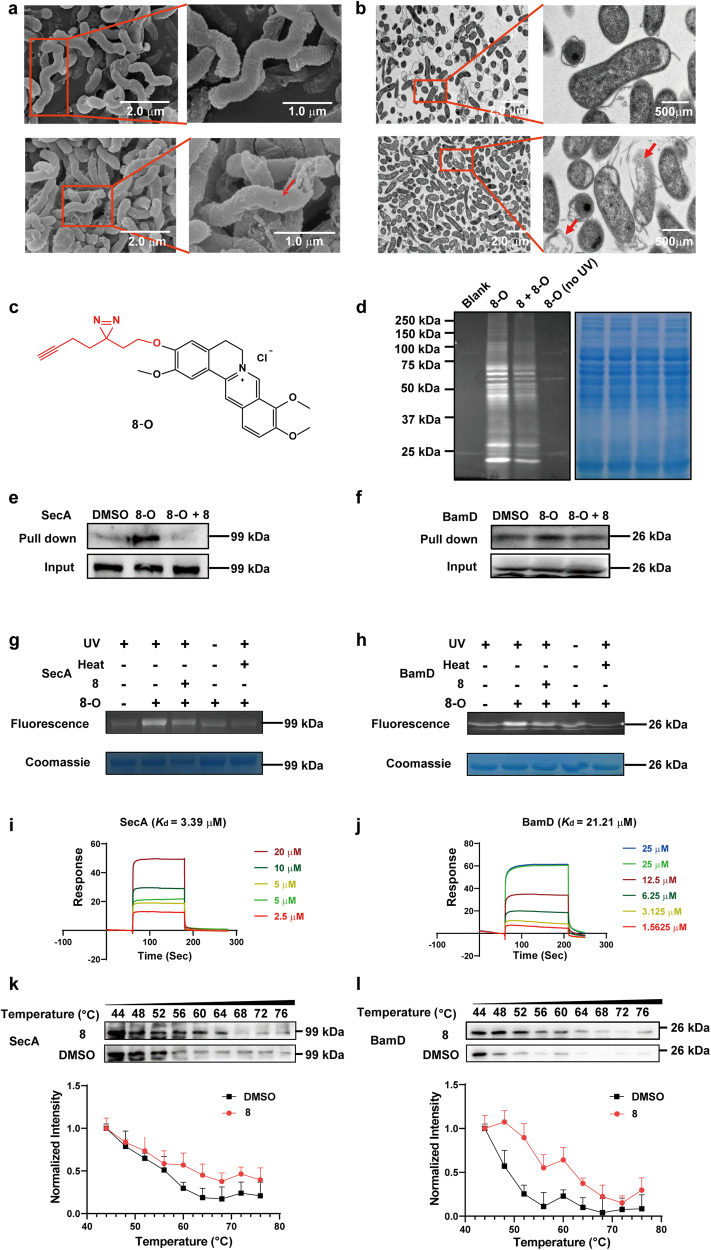
Mechanism of action and direct targets exploration on compound **8**. **a**, **b** Images for morphology of *H. pylori* under electron microscope (**a**) SEM images of *H. pylori* treated without (upper) or with (lower) **8**. **b** TEM images of *H. pylori* treated without (upper) or with (lower) **8**. **c** The structure of the active photoaffinity probe **8**-**O**. **d** Cy3-labeled target proteins were identified using fluorescent gel imaging. SecA (**e**) and BamD (**f**) were pulled down from *H. pylori* by using probe **8**-**O** in immunoblot assay. SecA and BamD pulled down by **8**-**O** were competitively inhibited by **8**. The recombinant SecA (**g**) and BamD (**h**) proteins pulled down by **8**-**O** were competitively inhibited by **8**. Surface plasmon resonance (SPR) sensorgrams obtained on SecA (**i**)/BamD (**j**)-coated chips at different concentrations of **8**. The thermal stability of SecA (**k**)/BamD (**l**) proteins with or without **8**-treatment (*n* = 3)

Bacterial cell morphologic changes can provide valuable clues on the antibacterial mode of action, and are often used for pilot mechanism investigation. Therefore, we performed scanning electron microscopy (SEM) and transmission electron microscopy (TEM) analysis on *H. pylori* ATCC 43504 after the treatment of compound **8**. Bacterial cells were incubated with or without sub-MIC (1/2 MIC, 0.25 μg/mL) level of **8** for 2 days. The SEM and TEM analysis results showed that the integrity of the *H. pylori* outer membrane was compromised, and obvious perforations were observed compared to the untreated control group (Fig. 4a, b). This suggests that the mechanism of action of **8** might be related to its impact on the integrity of the bacterial outer membrane, which warrants further investigation.

### Direct targets of 8 against *H. pylori* are explored and verified through activity-based protein profiling (ABPP) technique

The effectiveness of **8** against both drug-susceptible and resistant *H. pylori* strains suggests that it might possess a unique mechanism of action distinct from those of the first-line antibiotics used for the treatment of *H. pylori* infection. Hence, it is of great significance to identify the direct targets of **8** and further elucidate its specific mechanism of action.

ABPP technique, a chemical biological tool for target protein exploration,^43–45^ was applied for the target fishing and identifying of **8** in this study, and the workflow of the specific process was described in Supplementary Fig. S5. Due to the lack of functional groups of **8** that can form covalent bonds with its target proteins, a photoaffinity probe (**8**-**O**, Fig. 4c) of **8** containing a diazirine photo cross-linking tag and an alkynyl functional group on position 3 was constructed. As mentioned above, mono substitution at position 3, and di-substitutions at positions 3 and 13 were beneficial for anti-*H. pylori* activity. Considering both structural similarity and synthetic feasibility, we opted for a probe design with a mono substitution at position 3. To make sure that probe **8**-**O** possessed a similar mechanism as compound **8** and is suitable for target exploration, we assessed the effects of **8**-**O** on the integrity of the *H. pylori* membrane through SEM and TEM analysis. As shown in the Supplementary Fig. S6, similar to compound **8**, probe **8**-**O** induced rupture and perforation of the *H. pylori* outer membrane. Subsequently, the probe’s activity against *H. pylori* was evaluated. As expected, **8**-**O** exhibited comparable potency against the tested strains, with MICs ranging from 0.5–2 μg/mL as illustrated in Supplementary Table S8, indicating a similar mechanism with **8**. Consequently, **8**-**O** was deemed a viable functional probe for subsequent target exploration and verification.

Following by the addition of probe **8**-**O** (25 μM) to the lysate of *H. pylori* ATCC 43504, the mixture was incubated for 1 h (Supplementary Fig. S5). Upon exposure to 365 nm light, the diazirine photo cross-linking tag of **8**-**O** could generate free radical fragments. These fragments could then form covalent bonds with adjacent hydroxyl groups of target proteins. Next, the alkyne reporter group of the **8**-**O**/protein conjugate was coupled with an azide-modified fluorescent dye (Cy3) *via* a click reaction. The Cy3-labeled complex was separated using SDS-polyacrylamide gel electrophoresis (SDS-PAGE), with DMSO treatment serving as the blank control. Fluorescent bands with molecular weights (MW) ranging from 25–150 kDa were observed, and the addition of **8** competitively weakened several of these bands, as depicted in Fig. 4d. This result demonstrated that **8**-**O** might partially occupy the binding sites of **8**’s targets, and was suitable for further verifications as a chemical tool. Similarly, a biotin-labeled complex was formed by coupling **8**-**O**/protein conjugate with biotin-azide (Supplementary Fig. S5). After being purified and enriched, the complex was identified through liquid chromatography-tandem mass spectrometry (LC-MS/MS) analysis in three biological replicates. Totally, 24 proteins were identified twice in the analysis (Supplementary Table S9). Among these, two proteins belonging to the bacterial general secretory pathway (Sec pathway) and β-barrel assembly machinery (BAM), namely protein translocase subunit SecA (SecA) and outer membrane protein assembly factor BamD (BamD), were selected for further verification, respectively. Since Sec pathway and BAM complex are known to be responsible for transporting and assembling the majority of OMPs to the outer membrane, targeting this system could potentially affect the integrity of the bacterial outer membrane, which is consistent with the findings in SEM and TEM analysis on **8**-treated *H. pylori* cells. Thus, SecA and BamD were given priority for further investigation.

### Specific interactions between 8 and SecA/BamD

Firstly, after pre-treatment of **8** in live *H. pylori*, SecA and BamD were successfully confirmed to be the potential direct targets of **8** through immunoblot assays using the **8**-**O** probe in the pull-down experiments (Fig. 4e, f). Obvious competitive inhibition could be detected when **8** was pre-treated in situ, indicating possible specific interactions between **8** and these two proteins. Meanwhile, the recombinant *H. pylori* SecA and BamD proteins were also expressed and purified for further verification. In the presence of both UV (365 nm) exposure and the active probe **8**-**O** treatment, SecA/**8**-**O** conjugate with Cy3-labeling was successfully pulled down (Fig. 4g). Whereas, the fluorescent band was significantly weakened when either UV exposure or the active probe was absent, indicating the necessity of covalent bond formation between SecA and **8**-**O** for successful pull-down. The fluorescence also faded when SecA was pre-treated with **8**, indicating possible competitive inhibitions. Moreover, the fluorescent band of the **8**-**O**/SecA complex almost vanished under the condition of 95 °C, suggesting that the active labeling of **8**-**O** binding with SecA only occurred in the natively folded state rather than in the heat-treated unfolded state. Similar results were also observed in the BamD treatment group (Fig. 4h).

It was found that **8** could dose-dependently bind to immobilized SecA and BamD with *K*_d_ values of 3.39 and 21.21 μM (Fig. 4i, j), respectively, in surface plasmon resonance (SPR) analysis. These results further confirmed the direct interactions between **8** and SecA or BamD. Besides, the cellular thermal shift assay (CESTA) was applied for further validation of their specific interactions, as displayed in Fig. 4k, l. Taking DMSO as the blank control, the thermal stability of the SecA protein decreased with a serial increase in temperatures ranging from 44 to 76 °C. However, with the addition of **8**, the stability of SecA improved significantly, indicating the possible formation of an **8**/SecA complex. The same trend was observed for BamD. These findings demonstrated that **8** might serve as a potential substrate of SecA as well as BamD and enhance the thermostability of these two proteins.

### Active binding site analysis between 8 and SecA/BamD

**Fig. 5:**
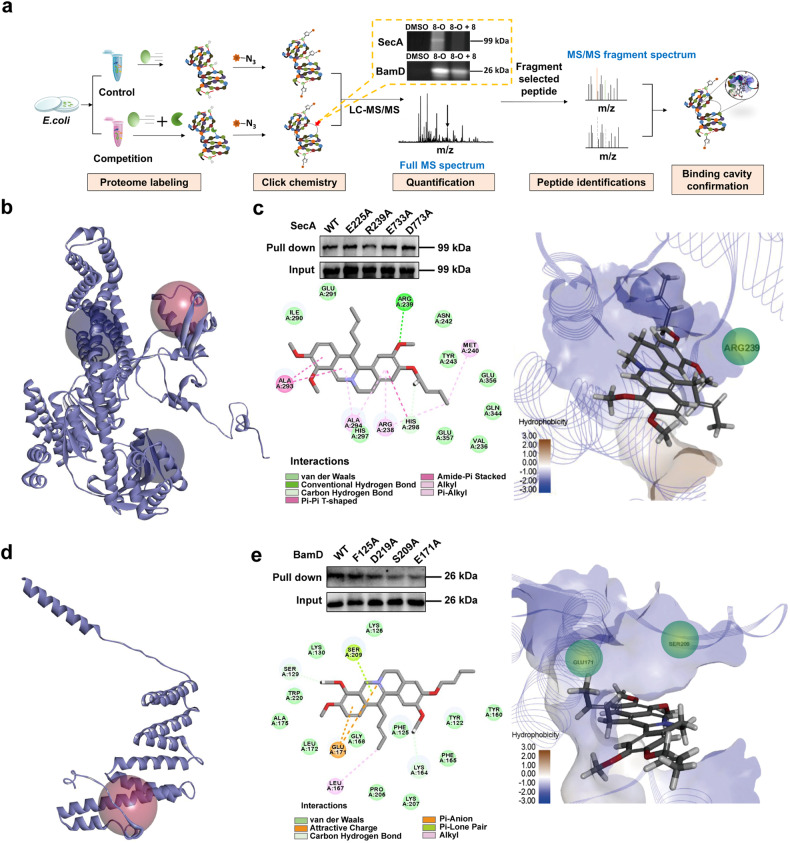
The exploration of active binding sites between **8** and SecA/BamD. **a** Experimental workflow for binding site and interaction residues investigation and validation based on LC-MS/MS analysis. The predicted docking patterns between **8** and SecA (**b**)/BamD (**d**) were performed by Discovery Studio 4.5 software based on the peptide fragment difference identification results of LC-MS/MS analysis. Specific binding pattern between **8** and SecA (**c**)/BamD (**e**)

To further figure out the specific binding sites and amino acid residues interacting with **8**, protein mass spectrometry analysis was conducted. As shown in Fig. 5a, *Escherichia coli* (*E. coli*) strain Rosetta overexpressing *H. pylori* SecA or BamD was pretreated with or without **8** before probe **8**-**O** was added. After proteome labeling and coupling with biotin, the specific peptide differences between the probe treatment and competitive inhibition group were analyzed through peptide fragment identification. Mass spectrometry analysis of the characteristic peaks was performed on the specific peptides of SecA/BamD, which might interact with **8**. These characteristic peaks revealed that three different active cavities of SecA might serve as the potential binding sites of **8** (Supplementary Fig. S7). Then, the docking pattern analysis (Fig. 5b) was simulated in Discovery Studio 4.5 software (BIOVIA, San Diego, California, USA) for the prediction of the dominant contribution of each amino acid residue in these three cavities, and four potential residues forming hydrogen-bond interactions were selected for single-mutation verification. After being single mutated to alanine, the specific binding site was verified (KAENLFGVDNLYKIENAALSHHLDQALK), and 239-arginine inside this cavity was found to play a key role in SecA-**8** interaction (the bright red ball, Fig. 5c). The two- and three-dimensional specific binding modes were displayed in Fig. 5c. Similarly, two adjacent peptide segments of BamD in space (one cavity), including “YRPYVEYMQIKFILGQNELNRAIANVYK” and “IDETLEK”, might contribute together to the interaction between BamD and **8** (Supplementary Fig. S8). Guided by the docking pattern and single mutation analysis, 171-glutamic acid and 209-serine were further confirmed to play the key roles among these residues. These findings provide solid evidences for the therapeutic targets verification of **8** and valuable insights for the exploration of novel candidates against *H. pylori*.

### Transcriptome analysis and RT-qPCR validation of the disturbance of 8 to OMPs

**Fig. 6:**
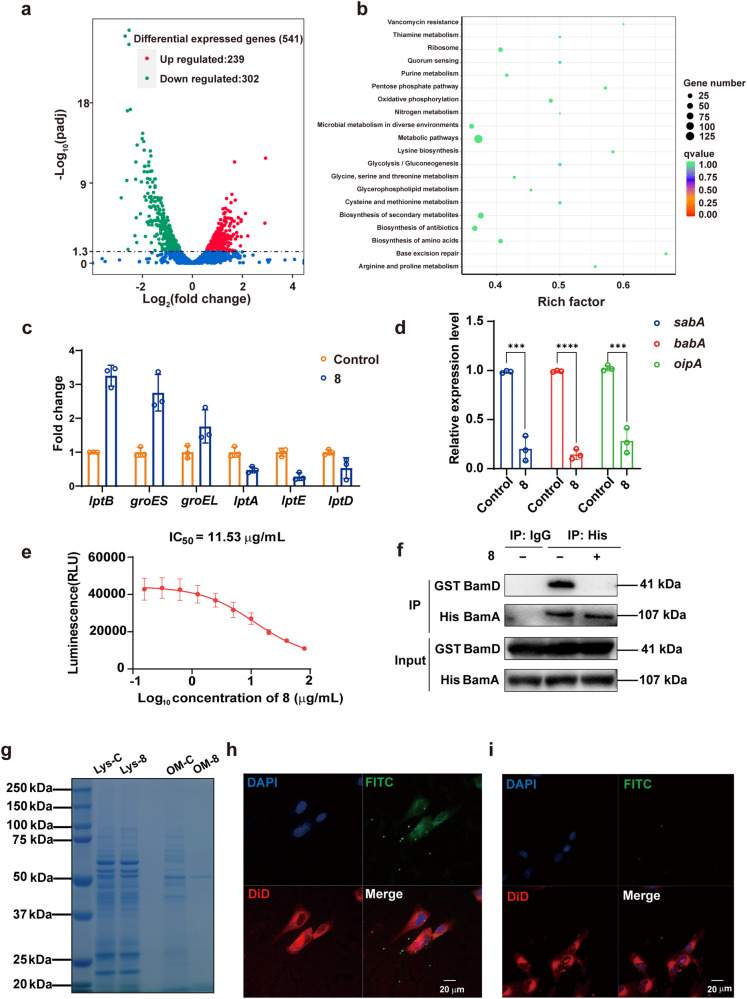
Compound **8** disturbs the OMPs related gene transcription and inhibits the protein function of SecA and BamD. **a**, **b** Transcriptome analysis of *H. pylori* with or without the treatment of **8** (*n* = 3). **a** Volcano plot analysis (Red dots: 239 up-regulated genes; Green dots: 302 down-regulated genes), and (**b**) KEGG analysis. **c** The differential expression genes at transcriptional level related to the OMPs secretion and transport dysfunction. **d** RT-qPCR verifications on gene transcription of the key *H. pylori* OMPs after the treatment of **8** (*n* = 3). **e** Inhibition of **8** on the ATPase activity of SecA (*n* = 3). **f** The interaction of BamA and BamD was inhibited by **8** in Co-IP analysis. **g** The change of the total amount of *H. pylori* OMPs after the treatment of **8**. **h**, **i** Confocal analysis on adhesive effect of **8**-treated *H. pylori* to GES-1 cells. No treatment group (**h**); **8** treatment group (**i**). For cell nucleic acid staining: 4’,6-diamidino2-phenylindole (DAPI); for cell membrane staining: 1,1’-Dioctadecyl-3,3,3’,3’-tetramethylindodicarbocyanine, 4-chlorobenzenesulfonate salt (DiD); for bacteria staining: fluorescein isothiocyanate (FITC)

Transcriptomic analysis was performed to gain comprehensive understanding of the antibacterial mechanism of **8** and verify its impact on OMPs (Fig. 6a, b). The inhibition of Sec pathway has been reported to impair the secretion of unfolded intracellular OMPs into the periplasmic space, leading to the over accumulation of OMPs within the intracellular space.^46^ As depicted in the Kyoto Encyclopedia of Genes and Genomes (KEGG) analysis, ribosome synthesis related genes were obviously down-regulated, which might due to the excessive accumulation of intracellular proteins. Specifically, after the treatment of **8,** *groEL* and *groES* responsible for intracellular protein folding were significantly up-regulated, which might be used to deal with the excessive unfolded proteins (Fig. 6c). Lipopolysaccharide (LPS) transport highly dependents on Lpt machinery system, which consists of LptB located in cytoplasm and the other components in inner membrane (LptF, LptG), periplasmic space (LptA, LptC) or outer membrane (LptD, LptE). The impaired outer membrane transport will also result in a hampered LPS transport. It is worth noting that, as a cytoplasmic protein, the transcriptional level of LptB was significantly up-regulated after the treatment of **8** for the compensation of LPS deficiency in outer membrane. While as the Sec and Bam pathway was suppressed, the proteins located outside the inner membrane (LptA, LptD, LptE) could not be transported out and stacked in cytoplasm, which led to a negative regulation in the transcription of their coding genes (Fig. 6c). The transcription levels of *H. pylori* adhesion proteins in outer membrane, including BabA, SabA, and OipA were also significantly decreased in the transcriptome study (data not shown) and RT-qPCR validation (Fig. 6d). Collectively, these data suggest that the treatment of **8** arouses OMP aggregation in the cytoplasmic and periplasmic spaces and ineffective transportation, which is consistent with the Sec pathway and Bam machinery dysfunction.

### Inhibition of protein functions of SecA and BamD by 8

SecA plays an indispensable role in the Sec complex as an ATPase.^47^ Therefore, the ATPase activity of SecA in the presence of **8** was measured. As depicted in Fig. 6e, **8** could dose dependently inhibit SecA, with an IC_50_ value of 11.53 μg/mL. Furthermore, to demonstrate the potential of SecA as an anti-*H. pylori* target, a previously reported SecA inhibitor CJ-21058 (IC_50_ = 7.0 μM)^48^ was evaluated for its anti-*H. pylori* potency. The MIC values of CJ-21058 against tested *H. pylori* strains were found to be in the range of 4-8 μg/mL (Supplementary Table S10), suggesting that SecA has the potential to be an attractive anti-*H. pylori* target and screening for SecA inhibitors could be an effective strategy for developing novel anti-*H. pylori* candidates.

In gram-negative bacteria, the assembly of OMPs requires the Bam machinery complex, in which BamA is the central component. The β-barrel domain of BamA interacts with four lipoproteins, including the essential lipoprotein BamD that directly interacts with BamA, and the other accessory lipoproteins BamB, BamC, and BamE.^49^ BamD facilitates the delivery of OMP substrates to BamA β-barrel and the subsequent assembly. To investigate if the function of BamD was affected by **8**, a Co-Immunoprecipitation (Co-IP) test was performed using GST-tagged BamD and His-tagged BamA. As depicted in Fig. 6f, BamD exhibited a strong interaction with BamA, and this effect was suppressed by **8**, indicating that **8** might inhibit the function of the BAM machinery by affecting the BamA-BamD interaction.

## Discussion

The prevalence of *H. pylori* infection remains significant worldwide. In the context of increasing rates of drug-resistant *H. pylori* strains globally, it is of great importance to develop new treatment strategies that can overcome the limitations of current regimens. The use of agents with novel mechanism for combating *H. pylori* is essential to potentially avoid cross-resistance with existing antibacterials.

In this study, a deep learning model was successfully established for the prediction of new anti*-H. pylori* agents, and a novel candidate **8** specifically acting on *H. pylori* was predicted. The results of the proposed deep learning model demonstrate a promising accuracy in identifying potential anti-*H. pylori* agents, thereby offering a possible tool in the pursuit of novel therapeutic interventions against this medically significant pathogen. The integration of deep learning and traditional drug discovery holds immense promise for addressing the growing threat of this pathogen, advancing the possible process towards more effective and sustainable therapeutic solutions. This approach may not only accelerate the anti*-H. pylori* candidate discovery but also provide a more targeted and informed approach for compound selection.^25^ Building upon the accurate prediction and validation on a small-scale dataset in this study, we intend to further conduct predictions and validations on larger datasets. Also, leveraging this deep learning model, structure-based rational fragment growth might be an efficient approach to establish an effective compound library against *H. pylori* including drug-resistant strains, which can be utilized for the initial or recurrent eradication therapy.

Due to the efficacy of **8** against multidrug-resistant *H. pylori* strains (those resistant to first-line agents such as MET, CLA, and AMX) and its synergistic or additive effects when combined with CLA or AMX we observed in this study, **8** may offer a potential alternative to one or two antibiotics to tackle the severe drug resistance. The poor compliance of the patients with the current eradication therapies resulting from the prolonged high-dose and the pronounced impact of antibiotics on intestinal microbiota are factors that limit the efficacy of the current regimens used clinically. The unique pharmacokinetic profile of **8**, characterized by its predominant gastric retention, may reduce its systemic side effects, dosing frequency, and course of treatment. Besides, less gastrointestinal flora disturbance by **8** enables its promising compliance. In addition, the favorable acid stability contributes to the efficacy of the mono-therapy of **8**, exhibiting comparable eradicative effects compared with both triple-therapy (OPZ + AMX + CLA) and the quadruple-therapy (OPZ + AMX + CLA + bismuth citrate) groups. The promising anti-*H. pylori* activity both in vitro and in vivo, favorable acid stability and gastric accumulation, low possibility of resistance evolution, and less perturbance on intestinal flora enable **8** an ideal candidate for *H. pylori* eradication.

ABPP is a technique in the field of proteomics that possesses several distinct advantages, contributing to its widespread adoption and application in diverse areas of biological research. ABPP facilitates the identification of potential therapeutic targets by profiling the active proteome in disease-relevant tissues or cells. These verified targets may be crucial for understanding the key players in disease pathways and for designing targeted therapeutic interventions. To demonstrate that probe **8**-**O** based target exploration was reliable, several verifications were conducted. Besides, to verify the reliability of the above-mentioned target exploration results, the same interactions between **8**-**O** and the specific binding sites of SecA/BamD were predicted by molecular docking, as depicted in Supplementary Fig. S9. Also, **8**-**O** could dose dependently inhibit SecA, with an IC_50_ value of 13.92 μg/mL (Supplementary Fig. S10). And a similar inhibitory effect on BamA-BamD interaction was observed in compound **8**-**O** treatment group (Supplementary Fig. S11). Thus, **8**-**O** could be regarded as an appropriate tool for ABPP-based direct target fishing, and verification results were highly consistent with the mechanisms speculated in morphologic observation and transcriptomic investigation in this study.

SecA and BamD, the validated targets of compound **8**, as indispensable component of the Sec complex and Bam machinery in OMPs transport, respectively, are essential for bacterial viability, making them promising targets for antibacterial therapy. The Sec pathway is involved in the translocation of unfolded preproteins and the insertion of membrane proteins across or into the cytoplasmic membrane, while the Bam machinery mediates the folding and insertion of β-barrel OMPs into the outer membrane of gram-negative bacteria. Currently, limited studies have been conducted on inhibitors of *H. pylori* Sec and Bam systems. Compound **8** was predicted and found to act on both systems, potentially blocking the entire transport process of OMPs. Consequently, the OMPs cannot be adequately assembled into the outer membrane. Therefore, the inhibition of SecA and BamD would lead to the deficiency in OMPs of the gram-negative bacteria. In order to verify **8** can lead to the OMPs deficiency of *H. pylori* by targeting SecA and BamD, the outer membrane of *H. pylori* from the untreated control group and 1/2 MIC (0.25 μg/mL) of **8**-treated group was isolated by super-centrifugation to quantify the change in the total OMPs amount after the treatment of **8**. As shown in Fig. 6g, a significant reduction in the amount of OMPs in the **8**-treated group was observed, which further confirmed the effect of **8** on transport and assembly system of OMPs. The reduction of outer membrane proteins was also observed after **8**-**O** treatment (Supplementary Fig. S12).

OMPs also play a critical role in the adhesion of *H. pylori* to the host cells as the important first step in persistent colonization.^50^ The absence of OMPs, especially adhesion-related proteins (BabA, SabA, OipA, etc.), could lead to a decline in the ability of *H. pylori* to adhere to gastric host cells, and this effect was observed for the *H. pylori* after the treatment of **8**. As shown in Fig. 6h, i, the nuclei, cell membrane, and *H. pylori* are stained with blue, red, and green fluorescence, respectively. After treatment with **8**, the green fluorescence was markedly weakened (Fig. 6i), suggesting a significant reduction of the adhesive or intracellular *H. pylori*.

Increasing attention has been paid to the OMP secretion and transport system of gram-negative bacteria in recent years.^51^ OMPs have been shown to be involved in bacterial adhesion and immune stimulation/evasion, and are essential for colonization and/or pathogenesis.^52^ As OMP transport and assembly system of *H. pylori* is relatively conserved and species-specific,^53^ agents targeting this system can be regarded as attacking the Achilles’ Heel of *H. pylori*. Therefore, agents targeting OMP transport system might be used alone or considered as an ideal component against *H. pylori*.

**Fig. 7:**
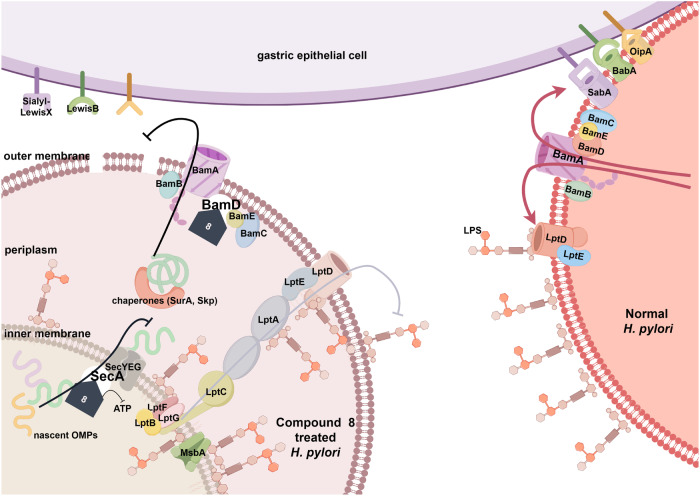
Cartoon of the mechanism of action of **8** (By Figdraw)

To sum up, a deep learning model was successfully established through correlating a set of over 10,000 molecules with anti-*H. pylori* potencies. This approach showcases a comprehensive and integrative methodology in anti-*H. pylori* drug discovery, leveraging both traditional experimental techniques and cutting-edge machine learning algorithms for enhanced candidate selection and prioritization. A novel BBR derivative **8** was positively predicted and found to exhibit satisfactory potencies against all tested *H. pylori* strains, including MDR strains, with an MIC range of 0.25-0.5 μg/mL, and demonstrated a good safety profile both in vitro and in vivo. The pharmacokinetic study revealed that the gastric concentration of **8** maintained higher than its MIC value up to 24 h, indicating an ideal gastric retention performance of **8** for gastrically colonized *H. pylori* eradications. Mono-therapy of **8**, as well as **8**-based dual (OPZ + **8**) and quadruple-therapy treatment (OPZ + AC + **8**) significantly decreased stomach bacteria load in vivo. Using ABPP technique, SecA and BamD belonging to OMP secretion and transport system were identified as direct targets of **8**, and further confirmed by pull-down, CESTA, and single mutation assays (Fig. 7). By down-regulating OMPs, **8** could also inhibit the adhesion of *H. pylori* to GES-1 cells. Based on its novel mechanism, the risk of cross-resistance of **8** with existing antibacterial agents was relatively low. In conclusion, compound **8**, with safety and unique pharmacokinetic properties, could exert anti-*H. pylori* activity through targeting SecA and BamD simultaneously, making it a potential new chemical entity for eradicating *H. pylori* including drug-resistant strains.

## Materials and methods

### Chemicals, reagents, bacterial strains and experimental animals

All the chemical reagents and anhydrous solvents were purchased from commercial sources (J&K scientific) and used without further purification. CLA, MTZ, LEV, and AMX were purchased from National Institutes for Food and Drug Control, Beijing, China. All *H. pylori* strains used in this study were obtained from the Collection Center of Pathogen Microorganism of Chinese Academy of Medical Sciences (CAMS-CCPM-A) in China, including the American Type Culture Collection (ATCC) reference strains. Two standard strains (*H. pylori* ATCC 43504 and ATCC 700392) and 31 clinically isolated strains, including CLA, MTZ, LEV, AMX resistant strains, were included in the current study.

All the animals were purchased from SPF Biotechnology Co. (Beijing, China). In vivo acute toxicity and pharmacokinetic studies were performed under the permission and supervision of the Ethics Committee of Institute of Medicinal Biotechnology, Chinese Academy of Medical Sciences and Peking Union Medical College, China (Approval No.: IMB-20210224D602). In vivo anti-*H. pylori* activity evaluations were performed under the permission and supervision of the Ethics Committee of Beijing Viewsolid Biotechnology Co. LTD, China (Approval No.: VS2126A00691 & VS2126A00720).

### Deep learning model construction

A training set of 938 compounds with known anti-*H. pylori* properties were first collected (801 reported anti-*H. pylori* compounds and 137 BBR derivatives). Compounds with MICs ≤16 μg/mL were defined as ‘active’ (label 1) and the compounds with MICs >16 μg/mL were defined as ‘inactive’ (label 0). The proposed deep learning framework represented compounds with a molecular graph, and extracted the molecular ECFP fingerprints preserving rich functional group information. Then, a deep graph neural network (Attentive FP) was applied to learn the embeddings of molecular graphs. The pre-train-then-fine-tuning paradigm was utilized to pre-train the deep learning model on some large-scale bioassays (12,700 compounds) from PubChem database and fine-tune the pre-trained deep learning model on the collection of 938 compounds. Finally, the molecular fingerprint features and molecular graph embeddings were concatenated to form the compound feature vectors and then an MLP layer was leveraged to predict their activity against *H. pylori*.

### Antimicrobial susceptibility tests in vitro

The MICs were determined by agar dilution method following the Clinical and Laboratory Standards Institute (CLSI) guidelines (M45) as follows. Briefly, the bacterial suspension adjusted to 2.0 McFarland density (about 10^8^ CFU/mL) was prepared from a Columbia agar plate containing 5% defibrinated sheep blood. Then, 1 μL of the bacterial suspension was directly inoculated onto the MH agar plates with 5% defibrinated sheep blood containing 2-fold serial dilutions of test compounds. The plates were incubated at 37 °C for 72 h under microaerobic conditions (10% CO_2_). *H. pylori* ATCC 43504 was used as a control strain. After incubation, the plates were examined visually, and the MICs were defined as the lowest concentration of a drug showing no growth. The MIC results were obtained from two independent experiments. AMX, CLA, MTZ, and LEV were tested as quality controls. The resistance breakpoints were defined as >0.125, >0.25, >8, and >1 μg/mL for AMX, CLA, MTZ, and LEV, respectively, according to EUCAST.

The agar dilution checkerboard assay was used to assess the combination effect of compound **8** and AMX/CLA on 25 *H. pylori* strains. MH blood agar (including 5% defibrated sheep blood) plates containing different concentration combinations of **8** and AMX/CLA were inoculated with bacterial suspension (adjusted to 2.0 McFarland density) spots of 1 μL. After incubation at 37 °C for 72 h under microaerobic conditions, the effect of the combination was evaluated using the FICI equation: FICI = (MIC of drug A in the combination/MIC of drug A alone) + (MIC of drug B in the combination/MIC of drug B alone). The combination was defined to be synergistic when the FICI was ≤0.5; additive when 0.5 < FICI ≤ 4; antagonistic when FICI > 4. The experiments were performed in triplicate on different days.

### Resistance development by serial passage

Bacteria were induced to develop resistance to drugs by continuously passaging bacteria grown in sub-MIC levels of antimicrobial agents. *H. pylori* ATCC 43504 was serially passaged in sub-MIC concentration of **8**, for 12 consecutive passages (36 days) to assess the potential resistance induction. AMX and CLA were tested under identical conditions as controls. Briefly, bacteria suspension was adjusted to a density of 2.0 McFarland and diluted 20-fold in Brain & Heart Infusion (BHI broth) supplemented with 10% (v/v) fetal bovine serum (FBS) containing **8**, AMX or CLA at the concentration of their 1/4 MIC, respectively. The inocula were incubated for 72 h at 37 °C with shaking under microaerobic conditions. The process was repeated for 12 consecutive passages. The MICs of each passage were determined together with the un-induced strain.

### Cell culture

GES-1 cells, HepG2 cells and 293T cells were cultured with DMEM complete medium (10% FBS, 1% penicillin/streptomycin was added to the medium), and H460 cells were cultured with RPMI-1640 complete medium (10% FBS, 1% penicillin/streptomycin was added to the medium) and placed in a constant temperature incubator at 37 °C and 5% CO_2_ nourish. When cells have grown to approximately 80–90% confluence, subculture was conducted at a ratio of 1:3.

### In vitro cytotoxicity assay (MTT assay)

Serial dilutions of test compounds, starting from 100 µM were incubated with GES-1, HepG2, H460 or 293T cells in 96-well plates for 24 h at 37 °C. Subsequently, 20 μL MTT (5 mg/mL) was added to each well and cultured for 4 h. After the removal of the culture medium, 150 μL DMSO was added to dissolve formazan crystals and the absorbance at 570 nm was measured on a fluorescent microplate reader (Synergy H1 model, BioTek, Agilent, USA). The TC_50_ values of test compounds were calculated by GraphPad Prism 8 (San Diego, CA).

### In vivo acute toxicity test

Kunming mice weigh 18-20 g, 6 in each group, half males and half females were used in acute toxicity evaluation. Compound **8** was administered orally once at the dose of 125, 250, or 500 mg/kg, and the animals were monitored and death recorded for 2 weeks. After 14 days post administration, blood was taken after fasting. The liver and kidney function of the mice was evaluated by detecting the GOT, GPT, BUN, and CRE.

### Pharmacokinetic study

C57BL/6 mice (20 ± 2 g, half males and half females) were housed in a temperature (22 ± 2 °C) and humidity (55 ± 6%)-controlled facility. The mice had free access to standard laboratory food and water, and food was withdrawn overnight before experiments. Thirty-two mice were randomly divided into eight groups (4 mice each time point), and blood samples and stomachs were collected at 0.5, 1, 2, 4, 6, 8, 12 and 24 h after a single oral dose of **8** (30 mg/kg). Plasma or stomach homogenate (50 μL) was mixed with 200 μL acetonitrile, vortexed for 2 min and centrifuged at 15,000 rpm for 15 min at 4 °C. The supernatant was directly injected into the LC-MS/MS system for quantitative analysis of **8**, with BBR as an internal standard. Quantification was performed in the selected reaction monitoring mode with (*m*/*z*)^+^ 336 → 320 (collision energy 20 eV) and 446 → 390 (collision energy 20 eV) for **8** and BBR, respectively. Concentration-versus-time profiles of **8** in plasma and stomach were obtained.

### Anti-*H. pylori* efficacy in vivo

Specific-pathogen-free female C57BL/6 mice (18 ± 2 g) were used for this study. Each mouse received 0.3 mL of 10^9^ CFU/mL *H. pylori* CCPM(A)-P-3722159 through oral gavage every 48 h, repeated four times (on day 1, 3, 5, and 7), and the infection was allowed to develop for 2 weeks. The infected mice were randomly assigned to four treatment groups (*n* = 5) to receive CMC, dual therapy (OPZ + **8**), triple therapy (OPZ + AC) and quadruple therapy (OPZ + AC + **8**), respectively. Mice were administered OPZ through oral gavage at a dose of 200 μg/kg 30 min before the administration of the assigned treatments. Compound **8** (30 mg/kg), AC (15 mg/kg AMX and 15 mg/kg CLA), and **8** + AC were administered once daily for a consecutive 5 days by oral gavage. The control group received an equivalent volume of CMC. Forty eight hours after the last treatment, mice were sacrificed, and the stomachs were harvested. The stomach was cut along the greater curvature, and the gastric content was removed and rinsed with PBS. The stomachs were cut into two longitudinal sections, and each section was weighed. The two sections were used for the assessment of bacterial colonization and histology (HE stain), respectively. For bacterial colonization, a gastric tissue section was suspended in 1 mL PBS and homogenized for *H. pylori* recovery. The homogenate was serially diluted and spotted onto a Columbia blood agar plate containing vancomycin (10 μg/mL), amphotericin (5 μg/mL), cefsulodin sodium (5 μg/mL), trimethoprim (5 μg/mL), colistin (0.3 μg/mL) and bacitracin (30 μg/mL). The plates were then incubated at 37 °C under microaerobic conditions for 3 to 5 days, and bacterial colonies were counted, and expressed as CFU per gram of stomach.

In addition to the above in vivo efficacy evaluation, the mono therapy of compound **8** was also performed compared with triple- and quadruple-therapy in the second batch of animal experiment with the same dosage of each drug as in the first batch.

### Scanning electron microscopy and transmission electron microscopy

The effects of **8** on the structure of *H. pylori* were examined via SEM and TEM. One hundred microliters bacterial suspension (~10^8^ CFU/mL) of *H. pylori* ATCC 43504 was inoculated onto Columbia agar plates with 5% defibrinated sheep blood containing **8** at 1/2MIC (0.25 μg/mL) for 48 h, collected and fixed with 2.5% glutaraldehyde in PBS for 24 h. The fixed bacteria were rinsed in fresh PBS, passed through an ethanol gradient for dehydration, dried, coated with gold, and observed by scanning electron microscope (Hitachi SU8020, Tokyo, Japan). The fixed organisms were washed and postfixed with 1% osmium tetroxide. Then the samples were washed, dehydrated in a graded series of ethanol, and embedded in Epon Araldite. Ultrathin sections containing the cells were placed on copper grids, stained with uranyl acetate and lead citrate, observed, and photographed with a TEM microscope (Hitachi, Tokyo, Japan).

### Experimental procedure for ABPP-based direct target exploration

After the protein concentration of *H. pylori* ATCC 43504 lysate was determined using Bradford protein assay (Bio-Rad USA), it was prepared into several 1 μg/μL aliquots using PBS buffer containing 1% SDS. Then they were treated with probe (**8**-**O**) DMSO solution with or without **8** (500 µM), and the mixtures were incubated at room temperature for 2 h followed by UV irradiation (~365 nm) for 20 min. Equal amount sample with different treatment was used for fluorescent tagging or biotin tagging. For each click reaction, SDS (0.1%), TCEP (1 mM), CuSO_4_ (1 mM), TBTA (100 µM) and Cy3-azide or biotin-azide (100 µM) was sequentially added to the lysates, incubated for 2 h, and then the labeled proteins were precipitated with acetone. For fluorescence detection, the samples were dissolved with 1×SDS loading buffer and boiled at 95 °C for 10 min. For LC-MS/MS and immunoblot detection, the samples were dissolved in PBS buffer with 1% SDS, and incubated with washed Pierce Streptavidin Magnetic Beads for 2 h. After washing 5 times with TBS-T, the beads were mixed with 1×SDS loading buffer, followed by boiling at 95 °C for 10 min. Samples were detected by gel fluorescence or immunoblot blot assay (Tanon 5200 system, Tanon, Shanghai, China).

The proteins were released from beads after 95 °C boiling and identified with a Label-free based LC-MS/MS approach (Beijing Qinglian Biotech Co., Ltd., China). MS was analyzed using Maxquant software (version 1.5.3.30) with the UniProtKB/Swiss-Prot *Helicobacter Pylori* ATCC 43504 database.

### Protein labeling and pull-down assay

*H. pylori* was pretreated with **8** or DMSO for 1.5 h and then treated with **8**-**O** for 0.5 h at room temperature, and lysed. After being 365 nm ultraviolet irradiated, click chemical reagents were used to introduce biotin, then the samples were enriched and purified with Pierce streptavidin magnetic beads. The immobilized beads were then washed three times with TBS-T buffer and mixed with 1 × loading buffer, followed by boiling at 95 °C for 10 min. The amount of target protein in the pull-down after enrichment of lysate and magnetic beads was detected by immunoblot analysis.

### SPR analysis

The SPR assay was performed on a BIAcore T200 biosensor system (GE Healthcare Life Sciences, Piscataway, NJ, USA) at 25 °C using a CM5 chip. The bindings of SecA or BamD at different concentrations of **8** were performed in 1 × PBS-P (GE Healthcare Life Sciences) at a flow rate of 30 μL/min for 120 s. After each binding reaction, a further dissociation time of 60 s was applied after each injection to allow the signal to return to the baseline.

### CESTA assay

For the temperature-dependent thermal shift assay, 50 µL of lysates (3 mg/mL) from SecA-overexpressing or BamD-overexpressing *E. coli* strains were incubated with DMSO or 500 µM of **8** at each temperature point from 44 to 76 °C for 4 min. The samples were centrifuged at 20,000 × *g* for 10 min at 4 °C to separate the supernatant and pellet. The supernatant (16 µL) was mixed with 4 µL of 5 × loading buffer and then separated on a 10% SDS-PAGE for immunoblotting analysis of SecA or BamD.

### Binding-site exploration between 8 and SecA/BamD

*E. coli* lysates (100 µL) with recombinant SecA or BamD (3 mg/mL in 10 mM PBS buffer) were incubated with **8** (400 µM) or DMSO respectively, and photo-labeled with 25 µM of the probe. The labeled proteins were washed with cold acetone and methanol, and then solubilized with 100 µL of 1% SDS/PBS. Then the samples were incubated with washed beads for 2 h at room temperature. After washing five times with TBS-T, the beads were reconstituted in 500 µL of water for the pretreatment and LC-MS/MS analysis.

### Molecular modeling analysis

An automated docking was carried out using the 3D structure of SecA (Alphafold code: AF-I0ZGP5-F1), BamD (Alphafold code: AF-I0ZHZ3-F1) and Discovery Studio 4.5 software. All conformers of **8** were interactively docked to active binding pockets selected based on previous experimental results using the Libdock protocol.

### Site-directed mutagenesis

The lysates of *E. coli* expressing SecA-, BamD-wild type (WT), SecA-E225A, R239A, E733A, D773A and BamD-F125A, E171A, S209A, D219A were preincubated with **8**-**O** (25 μM) at 25 °C for 0.5 h, and then incubated with biotin-streptavidin complex at 25 °C for 2 h. Following TBS-T buffer wash, the proteins that had attached to the beads were analyzed by immunoblotting using antibody to SecA or BamD. Site-mutated plasmids of SecA and BamD were obtained by seamless cloning and confirmed by sequencing (DIA-UP Biotech, Beijing, China).

### RNA extraction and transcriptome analysis

Briefly, *H. pylori* cells were incubated with/without 0.25 μg/mL **8** for 2 days, then collected. Extraction of RNA was carried out using an RNAsimple total RNA Kit (TIANGEN, China) according to the manufacturer’s instructions. RNA quantity measurement and transcriptome analysis was conducted by Allwegene Tech (Beijing, China). Then sequencing was performed in an Illumina Hiseq platform with paired ends mode. The RNA-Seq raw data was filtrated and employed to assemble the bacterial transcriptome using Cutadapt software and RNA-Seq reads were mapped using the Bowtie2 software against the genome of *H. pylori* ATCC 43504. The experiment was designed and performed with three biological replicates.

### Quantitative PCR with reverse transcription (RT-qPCR)

Total RNA was extracted as mentioned above. An aliquot (500 ng) of the total RNA from each sample was subjected to cDNA synthesis using HiScript® III All-in-one RT SuperMix (Vazyme, China). The housekeeping gene 16S rRNA was used as control. The cDNAs were quantified by qPCR using Taq Pro Universal SYBR qPCR Master Mix (Vazyme) on a qTOWER®3 Real-Time PCR Systems (Analytik Jena) according to the manufacturers’ instructions. Primers used in this study are listed in Supplementary Table S11. All assays were carried out in three independent experiments in triplicates. The relative RNA expression levels were calculated according to ΔΔ*Ct* with normalization to the 16S rRNA levels.

### SecA ATPase activity

Purified recombinant SecA was incubated at 37 °C with different concentrations of **8** for 1 h in a reaction buffer. ATP was then added, and the mixture was further incubated at 37 °C for another 40 min. The amount of ATP converted into adenosine diphosphate (ADP) was determined by luminescent ADP detection with an ADP Glo Kinase Assay kit (Promega) according to the manufacturer’s protocol.

### Co-IP assay

To prove that **8** influences the interaction between BamD and BamA, the solution of purified His-tagged BamA and GST-tagged BamD was treated with or without **8** for 2 h, immunoprecipitated with anti-His beads (Abbkine), and then assessed by immunoblot analysis.

### *H. pylori* OMPs extraction and quantification

*H. pylori* cells were harvested, washed and suspended in 20 mM Tris-HCl (pH 7.5). DNase and protease inhibitors were added to the cell suspension, and the bacteria cells were disrupted by repeated ultrasonication. Cell debris was removed by centrifugation (9000 × *g*, 20 min). Total membrane pellet was collected by centrifugation (50,000 × *g*, 40 min), resuspended in 20 mM Tris-HCl containing 2.0% sodium lauryl sarcosine, and incubated at room temperature for 30 min. The sarcosine-insoluble constituent (OMPs) was collected by centrifugation (50,000 × *g*, 30 min), washed with 20 mM Tris-HCl (pH 7.5), and dissolved in XT Sample Buffer (Bio-Rad) with 50 mM DTT.

### Fluorescence staining and *H. pylori* adhesion assays

Briefly, GES-1 cells were seeded and cultured on cover glass bottom dishes, and then infected with FITC-labeled *H. pylori*. After a 3-day incubation, *H. pylori* cells were harvested from agar plates with/without **8** exposures, re-suspended in 0.15 M NaCl and 0.1 M Na_2_CO_3_, and adjusted to 1.0 McFarland. Ten microliters of freshly prepared 1% FITC in DMSO were added to the suspension and incubated for 1 h in the dark. Bacteria were recovered by centrifugation at 3000 × *g* for 5 min, and resuspended in 1.0 mL PBS supplemented with 5% inactivated fetal bovine serum, 0.2% BSA and 0.05% Tween-20. The FITC-labeled *H. pylori* cells were added into the dishes and incubated 4 h at 37 °C. After three washes with PBS to remove nonadherent bacteria, paraformaldehyde PBS solution (4%) was applied to immobilize for 10 min. The cell membrane and nucleus were stained with DiD (5 μM, 20 min) and DAPI (10 μg/mL, 5–10 min) at room temperature sequentially, respectively. After fully removing the stains, the *H. pylori* adhesion to GES-1 cells were observed and imaged under confocal laser microscope (LSM710, Zeiss, Germany).

### Statistical analyses

Statistical analysis was performed with one-way ANOVA by SPSS 16.0 with all data points showing a normal distribution, *p < 0.05, **p < 0.01, ***p < 0.001, and ****p < 0.0001. No exclusion of data points was used.

### Supplementary information

SUPPLEMENTAL MATERIAL

Dataset 4

Dataset 1

Dataset 2

Dataset 3

Dataset 5

## Data Availability

The data of this study are available as reasonable consultation with the corresponding authors. The proteomics and protein sequence data have been deposited to the ProteomeXchange Consortium via the PRIDE partner repository with the dataset identifier PXD052333.
