# Supplementary material for: A deep learning-driven discovery of berberine derivatives as novel antibacterial against multidrug-resistant Helicobacter pylori
Source: Signal Transduct Target Ther. 2024 Jul 8;9:183. doi: 10.1038/s41392-024-01895-0 (PMC11228022; doi:10.1038/s41392-024-01895-0)
Supplement: Supplementary file 1 — SUPPLEMENTAL MATERIAL [file 41392_2024_1895_MOESM1_ESM.docx]

Supplementary Materials for

A deep learning-driven discovery of berberine derivatives as novel antibacterial against multidrug-resistant *Helicobacter pylori*

Xixi Guo^†,1^, Xiaosa Zhao^†,2^, Xi Lu^†,1^, Liping Zhao^†,1^, Qingxuan Zeng^1^, Fenbei Chen^1^, Zhimeng Zhang^1^, Mengyi Xu^1^, Shijiao Feng^1^, Tianyun Fan^1^, Wei Wei^1^, Xin Zhang^3^, Jing Pang*^,1^, Xuefu You*^,1^, Danqing Song*^,1^, Yanxiang Wang*^,1^, Jiandong Jiang^1^

Correspondence to: [pangjing@imb.pumc.edu.cn](mailto:pangjing@imb.pumc.edu.cn) (P.J.); [xuefuyou@imb.pumc.edu.cn](mailto:xuefuyou@imb.pumc.edu.cn) (Y.X.F.); [songdanqingsdq@hotmail.com](mailto:songdanqingsdq@hotmail.com) (S.D.Q.); [wangyanxiang@imb.pumc.edu.cn](mailto:wangyanxiang@imb.pumc.edu.cn) (W.Y.X.)

**This PDF file includes:**

Materials and Methods

Supplementary Synthetic Scheme

Figures. S1 to S12

Tables S1 to S11

^1^H, ^13^C NMR Spectra

Sources of deep learning database

**Materials and Methods**

General

All of the chemical reagents and anhydrous solvents were purchased from commercial sources (J&K scientific, Beijing, China) and used without further purification. Melting points (mp) were obtained with an MPA 100 OptiMelt Automated Melting Point System (Stanford Research Systems, California, USA). ^1^H NMR spectra were obtained on a Varian Inova spectrometer (Varian, San Francisco, CA, USA), and ^13^C NMR spectra were performed on a Bruker Avance III 400 spectrometer in DMSO-*d_6_* or CD_3_OD, with Me_4_Si as the internal standard. ESI high-resolution mass spectra (HRMS) were recorded on an Autospec Uitima-TOF mass spectrometer (Micromass UK Ltd, Manchester, UK). Flash chromatography was performed on a Combiflash Rf 200 (Teledyne, Nebraska, USA). The purities of all positive compounds were greater than 95%, as determined by high-performance liquid chromatography (HPLC). The HPLC traces and other analytical data for key target compounds **5**, **7** and **8** are provided in the Supporting Information as follows.

Synthetic Procedures

The synthetic route used for the preparation of all the 3-position mono-substituted (**1**–**6**, **9**–**13**) and 3-,13-position di-substituted derivatives (**7**–**8**) of BBR is presented in Supplementary Scheme 1. Taking the advantage of electrophilic feature on position 13, all the 3-,13-position di-substituted derivatives of BBR were synthesized through a novel one-step synthetic route. This contrasts with previously reported procedures that involved three or more steps. In the presence of sodium hydride (NaH, 2.0 eq) as the base, Jatrorrhizine was heated in DMF with substituted α-unsaturated halogenated hydrocarbons (1.1 eq or 4.0 eq) to produce the corresponding 3-monosubstituted derivatives (**1**–**6**, **9**–**13**) in yields ranging from 53% to 82%. Additionally, 3-,13-disubstituted derivatives (**7**–**8**) were obtained with yields ranging from 61% to 67%. Compared to previously reported procedures (13–17%), the overall yields of the disubstituted derivatives **7**–**8** were significantly improved. The products were separated using flash column chromatography with CH_3_OH/CH_2_Cl_2_ as the eluent.

General Synthesis Procedure of Compounds **1**–**4**, **11**, **12**

To a stirred solution of **1** (1.0 mmol) in anhydrous DMF, NaH (2.0 mmol) and catalytic equivalent of NaI were added and heated to 71 °C. Then, acid halide (4.0 mmol) were added to the reaction system overnight, then the reaction mixture was cooled, filtered, and purified using chromatography over silica gel using CH_2_Cl_2_/CH_3_OH as the gradient eluent to obtain (**1**–**4**, **11**, **12**) as turmeric solid.

General Synthesis Procedure of Compounds **5**–**6**, **9**–**10**

To a stirred solution of **1** (1.0 mmol) in anhydrous DMF, K_2_CO_3_ (4.0 mmol) and catalytic equivalent of NaI were added and heated to 71–120 °C. Then, α-unsaturated halogenated hydrocarbons (4.0 mmol) were added to the reaction system for 6 h, then the reaction mixture was cooled, filtered, and purified using chromatography over silica gel using CH_2_Cl_2_/CH_3_OH as the gradient eluent to obtain (**5**–**6**, **9**–**10**) as turmeric solid.

General Synthesis Procedure of Compound **13**

To a stirred solution of **1** (1.0 mmol) in anhydrous DMF, NaH (2.0 mmol) and catalytic equivalent of NaI were added and heated to 71 °C. Then, Sulfonyl halide (4.0 mmol) were added to the reaction system for 8 h, then the reaction mixture was cooled, filtered, and purified using chromatography over silica gel using CH_2_Cl_2_/CH_3_OH as the gradient eluent to obtain (**13**) as turmeric solid.

General Synthesis Procedure of Compounds **7**–**8**

To a stirred solution of **1** (1.0 mmol) in anhydrous CH_3_CN, NaH (2.0 mmol) and catalytic equivalent of NaI were added and heated to 71 °C. Then, α-unsaturated halogenated hydrocarbons (4.0 mmol) were added to the reaction system for 0.5–1 h, then the reaction mixture was cooled, filtered, and purified using chromatography over silica gel using CH_2_Cl_2_/CH_3_OH as the gradient eluent to obtain (**7**–**8**) as turmeric solid.

**Supplementary Synthetic Scheme**

Synthetic Schemes

**Reagents and conditions:**

(a) NaI, K_2_CO_3_, DMF, 71–120 ^o^C, 6 h; (b) NaI, NaH, ACN, 71 ^o^C, 0.5–1 h; (c) NaI, NaH, DMF, 71 ^o^C, 8 h; (d) NaI, NaH, DMF, 71 ^o^C, 12 h.

**Supplementary Figure 1**

**
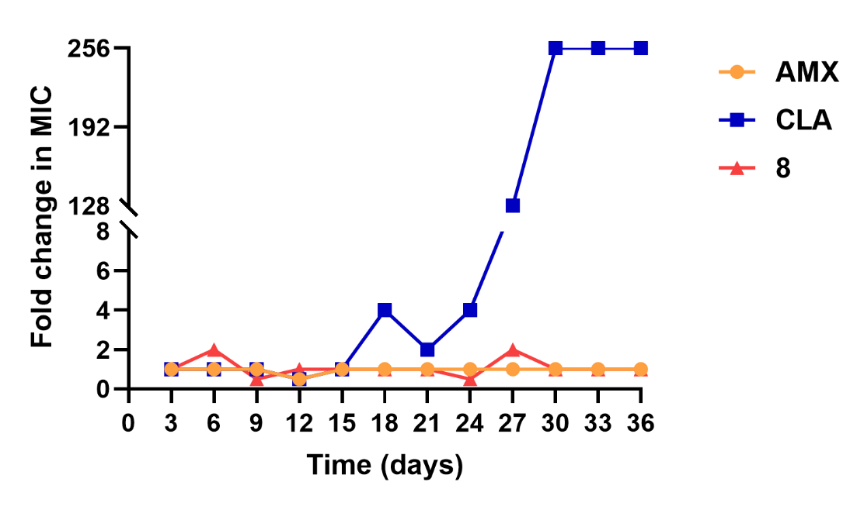
**

**Figure. S1.**

**Resistance development evaluations of AMX, CLA and compound 8.**

**Supplementary Figure 2**

**
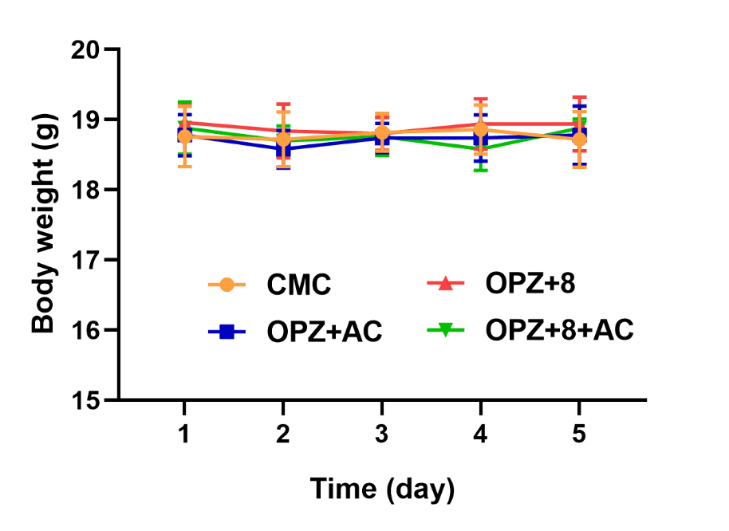
**

**Figure. S2**.

**Body weights of the mice in each group (*n* = 5).**

**Supplementary Figure 3**

**
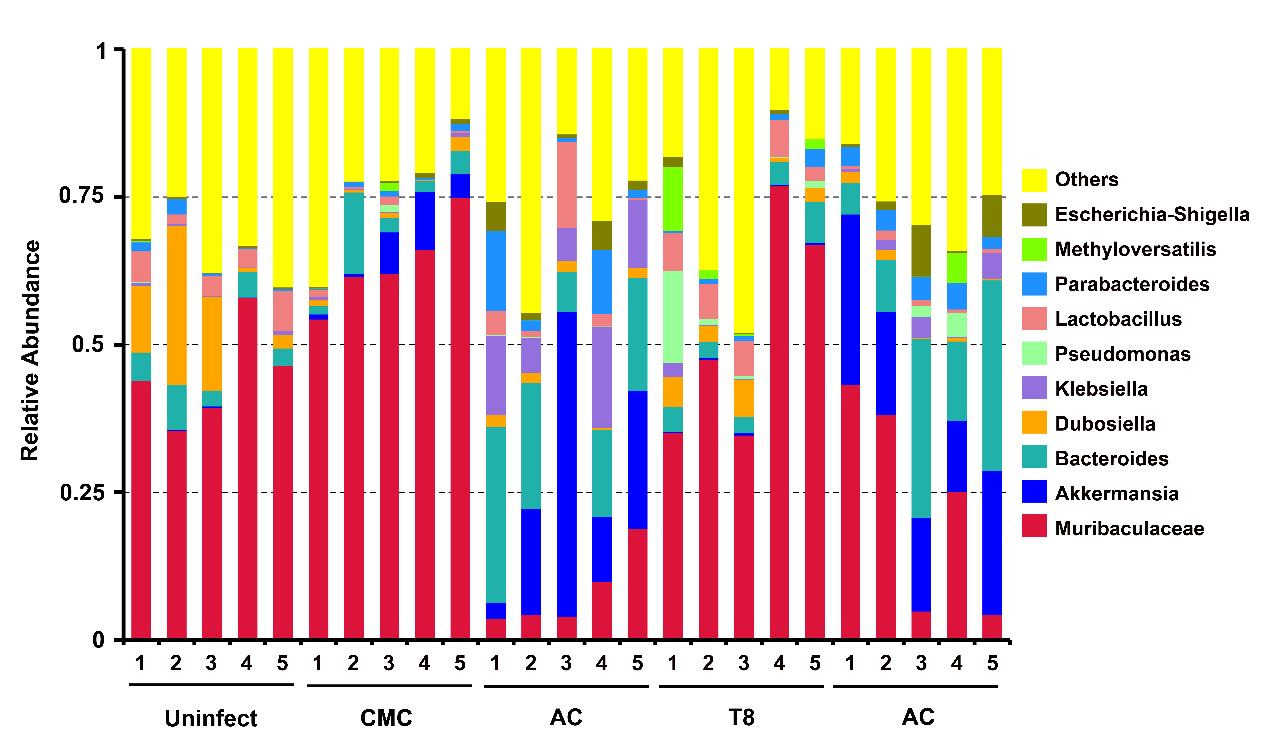
**

**Figure. S3**.

**A bar plot analysis of each sample’s abundance at the genus level (t****en bacterial genera with the highest abundance).**

Abbreviations: Uninfect, the uninfected group; CMC: CMC control group; AC: triple therapy group (OPZ + AC); T8: dual therapy group (OPZ + **8**); AC8: quadruple therapy group (OPZ + AC + **8**).

**Supplementary Figure 4**

**
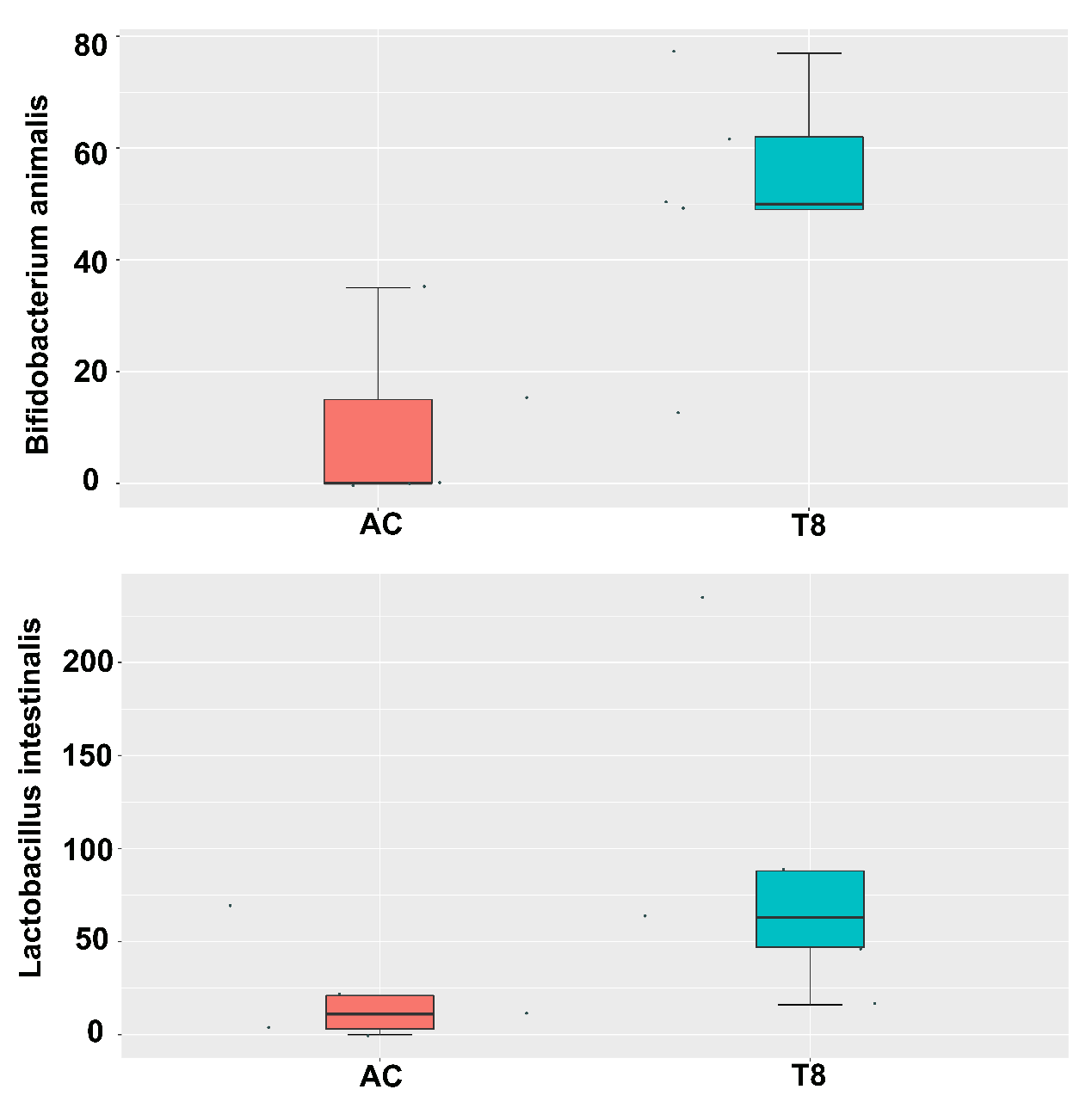
**

**Figure. S4**.

**The abundance of** ***Lactobacillus* and *Bifidobacterium*.**

Abbreviations: T8: dual therapy group (OPZ + **8**); AC: triple therapy group (OPZ + AC).

**Supplementary Figure 5**


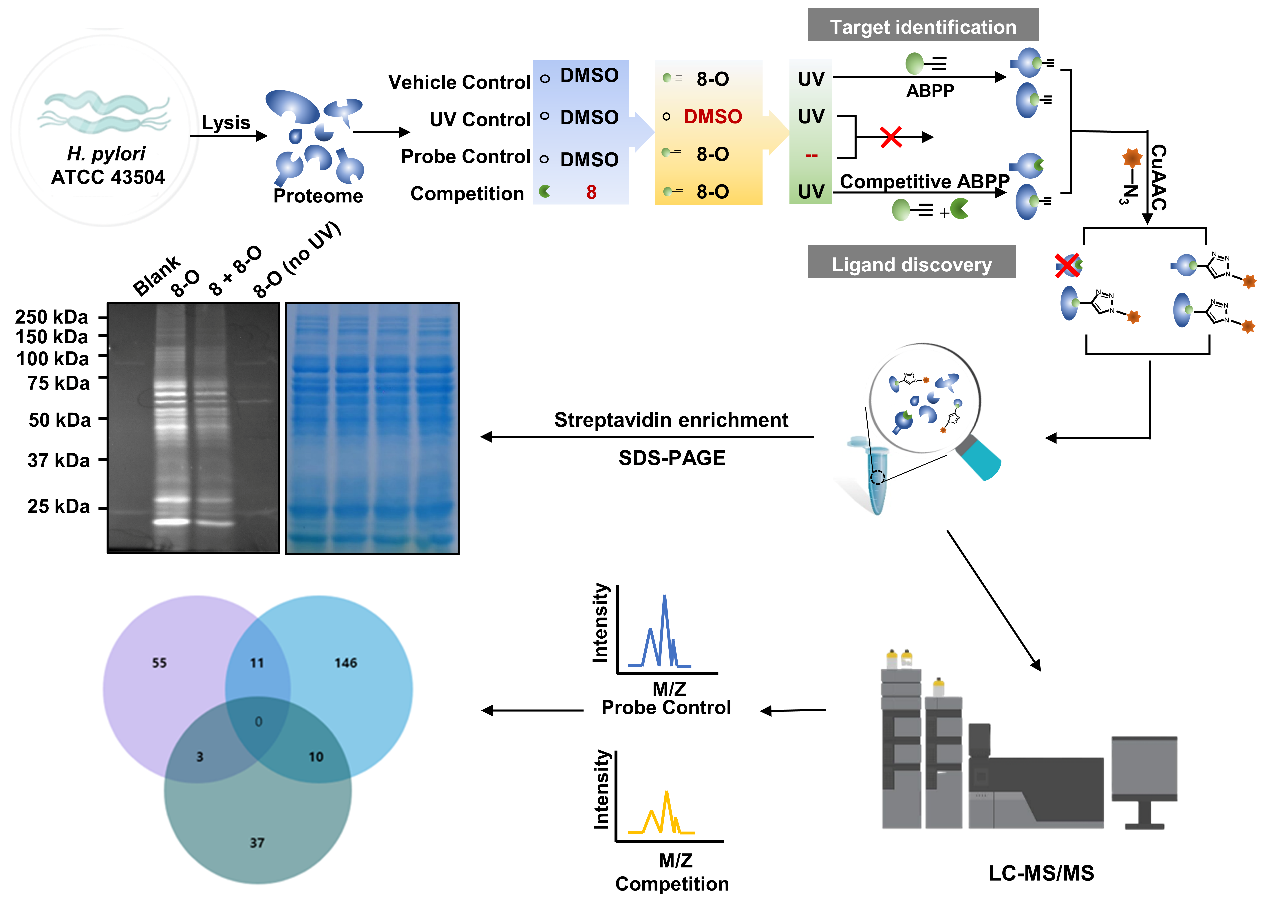


**Figure. S5**.

**ABPP experimental workflow for direct targets exploration based on LC-MS/MS analysis. (*n* = 3).**

**Supplementary Figure 6**


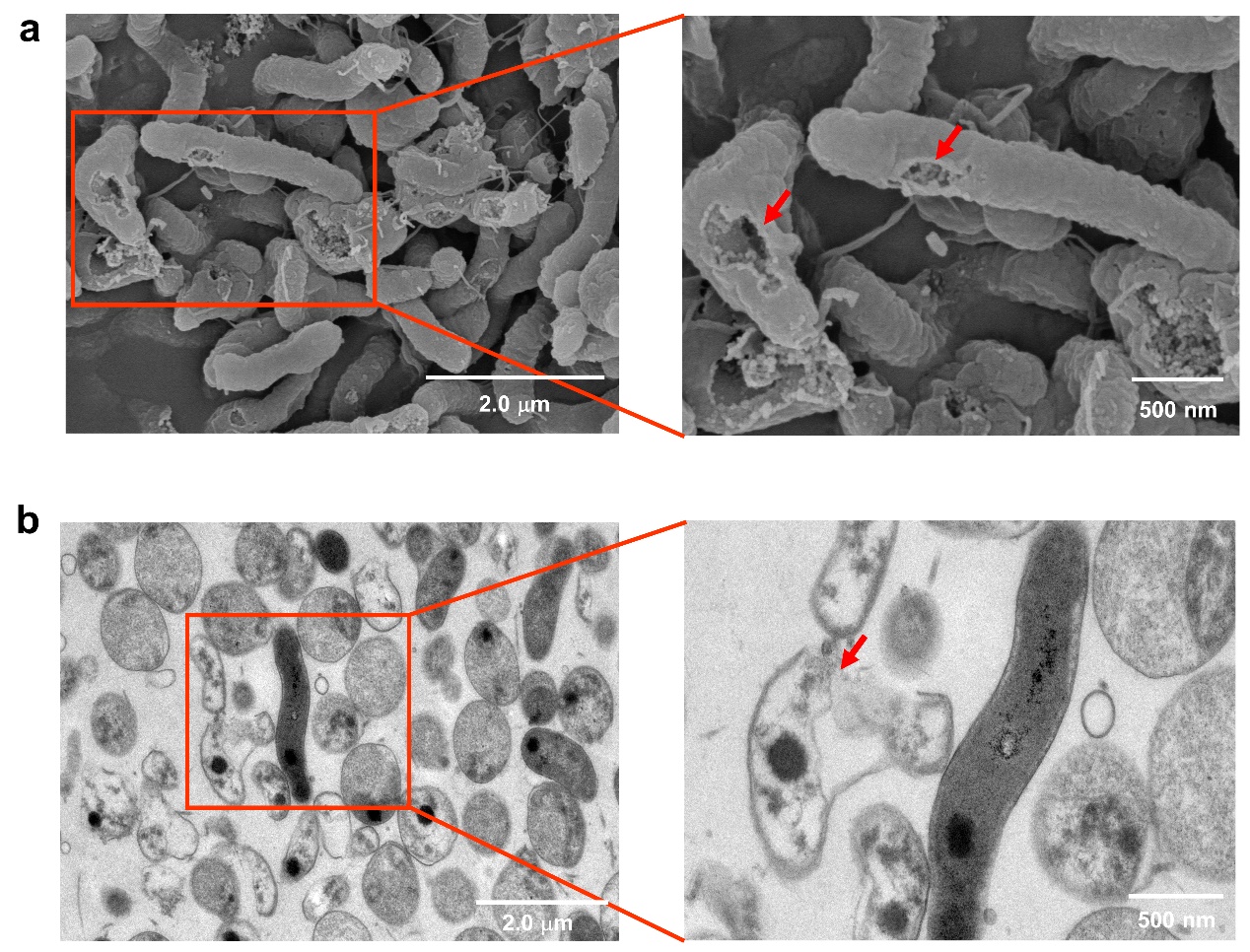


**Figure. S6**.

**Images for morphology of *H. pylori* under electron microscope.**

(**a**) SEM images of *H. pylori* treated with **8**-**O** (upper). (**b**) TEM images of *H. pylori* treated with **8**-**O** (lower).

**Supplementary Figure 7**


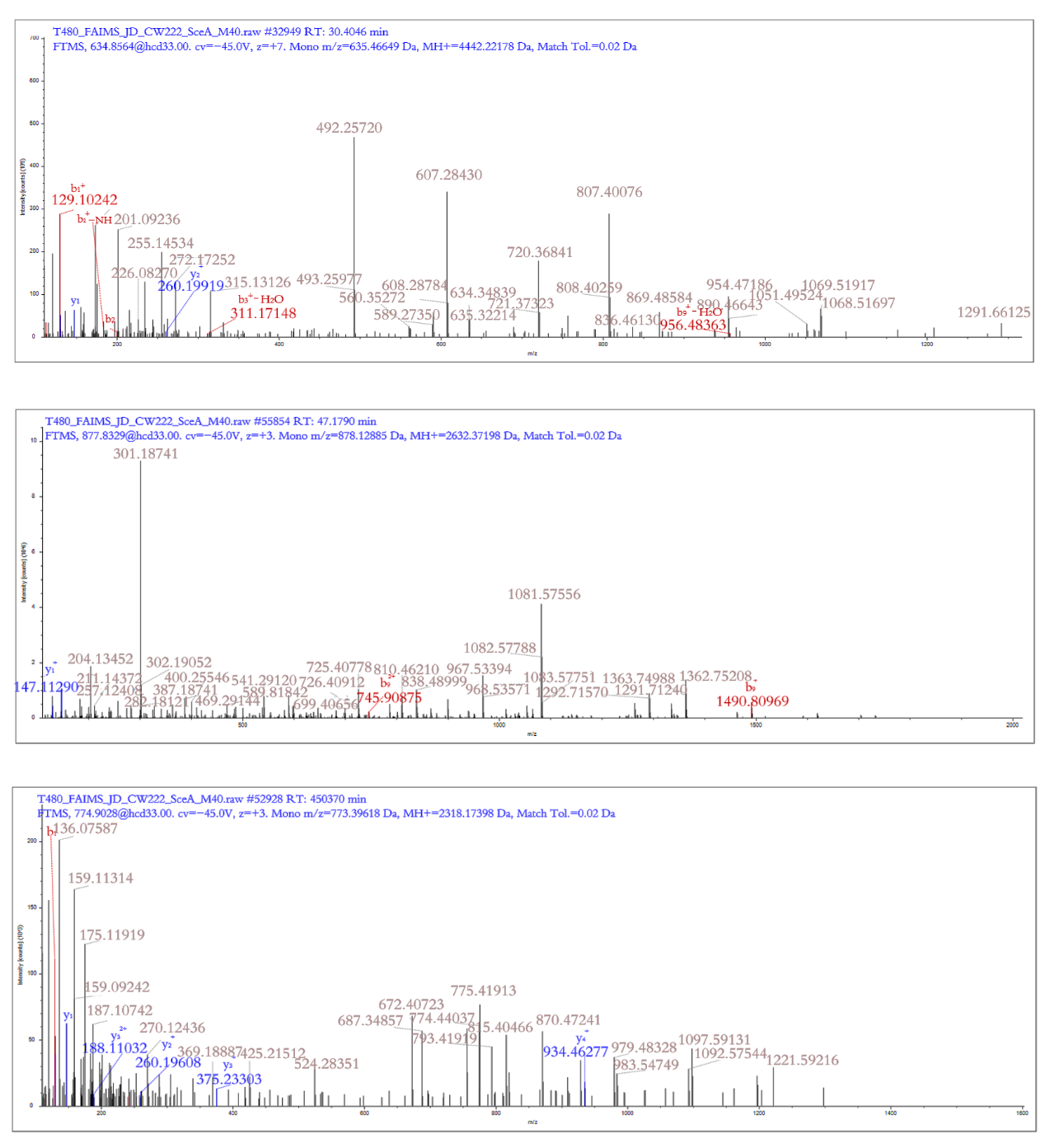


**Figure. S7**.

**Characteristic peaks of the specific peptides of SecA binding to 8 detected by mass spectrometry.**

**Supplementary Figure 8**


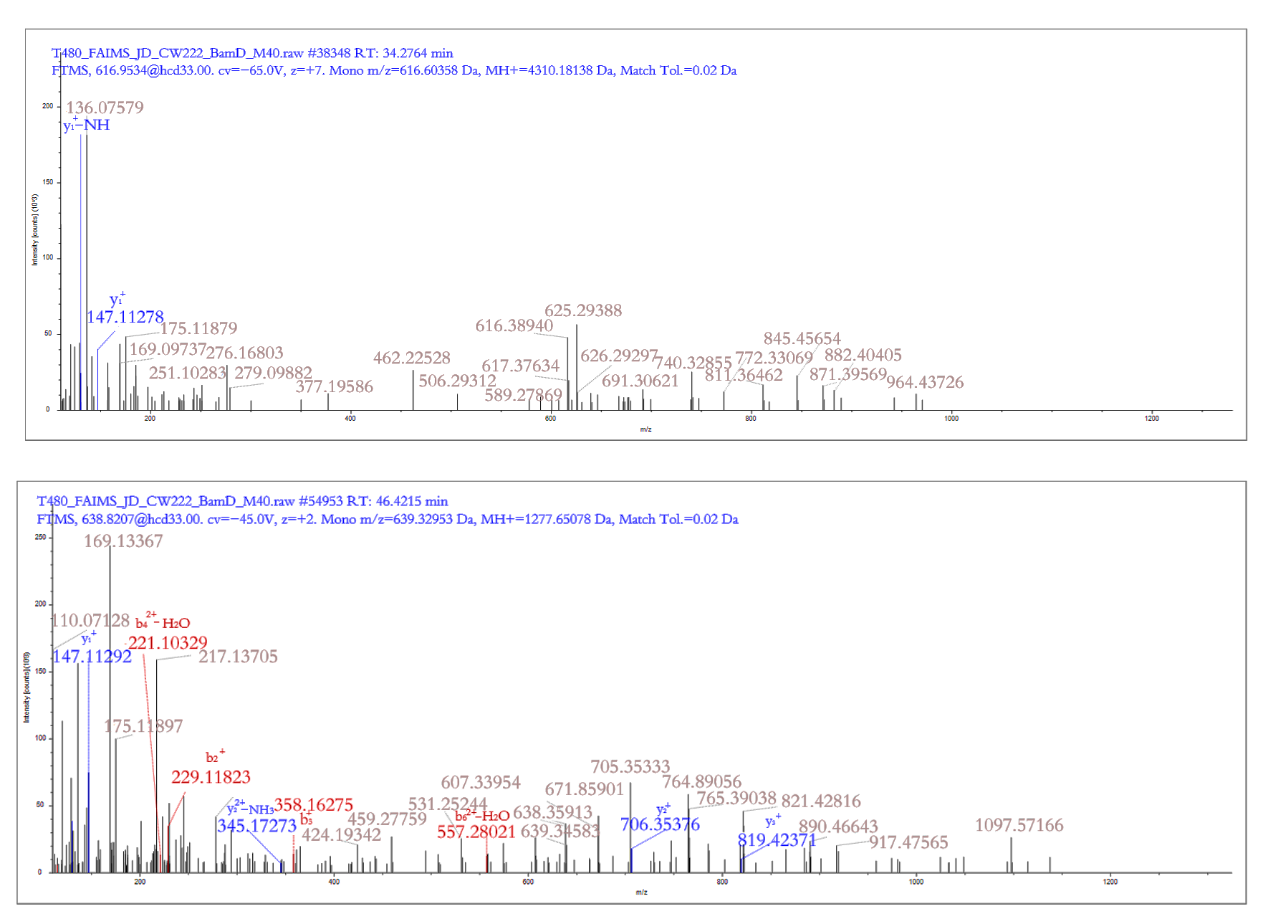


**Figure. S8**.

**Characteristic peaks of the specific peptides of BamD binding to 8 detected by mass spectrometry.**

**Supplementary Figure 9**

**
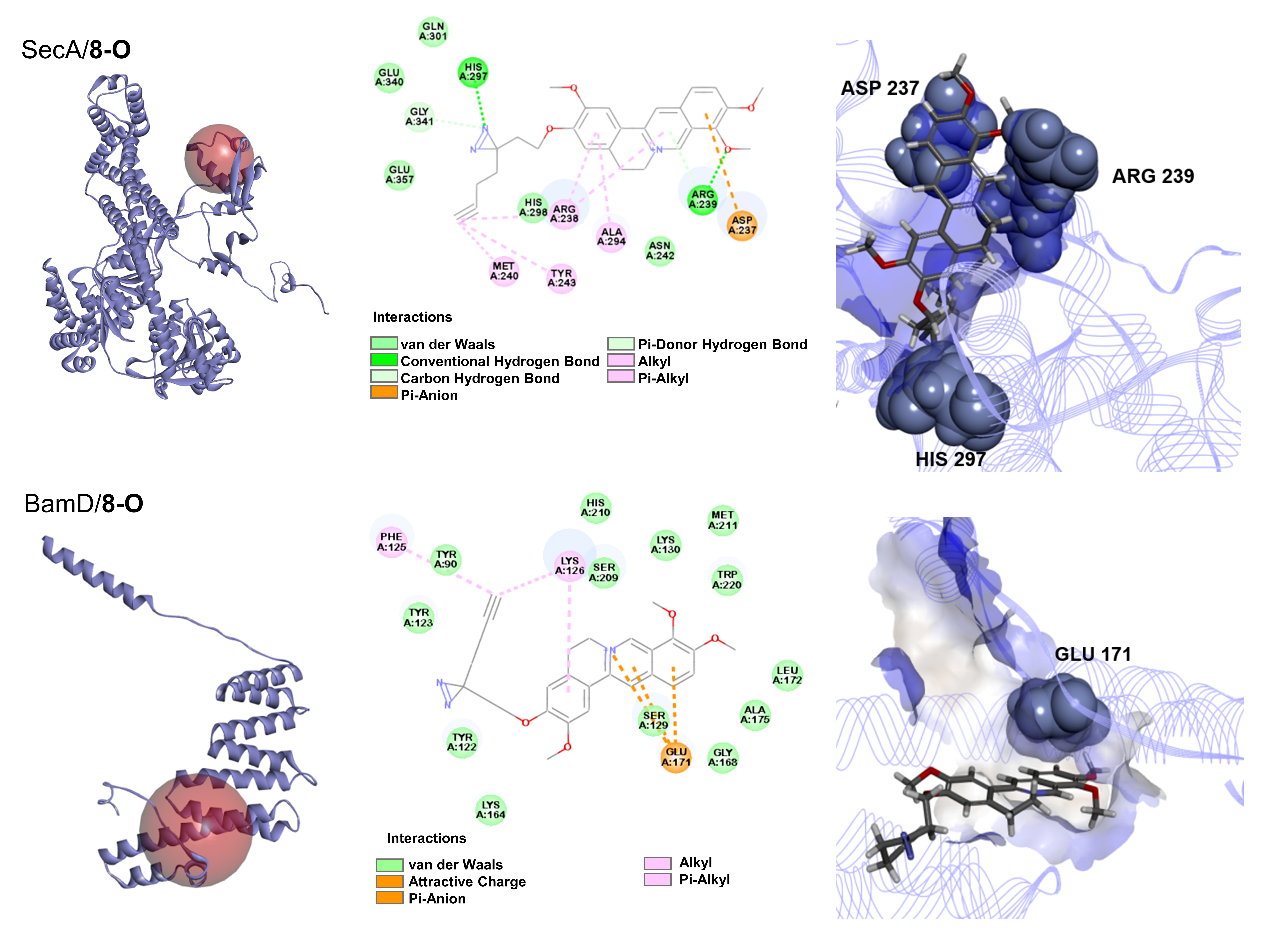
**

**Figure. S9**.

**The predicted docking pattern analysis between 8-O and SecA/BamD.**

**Supplementary Figure 10**


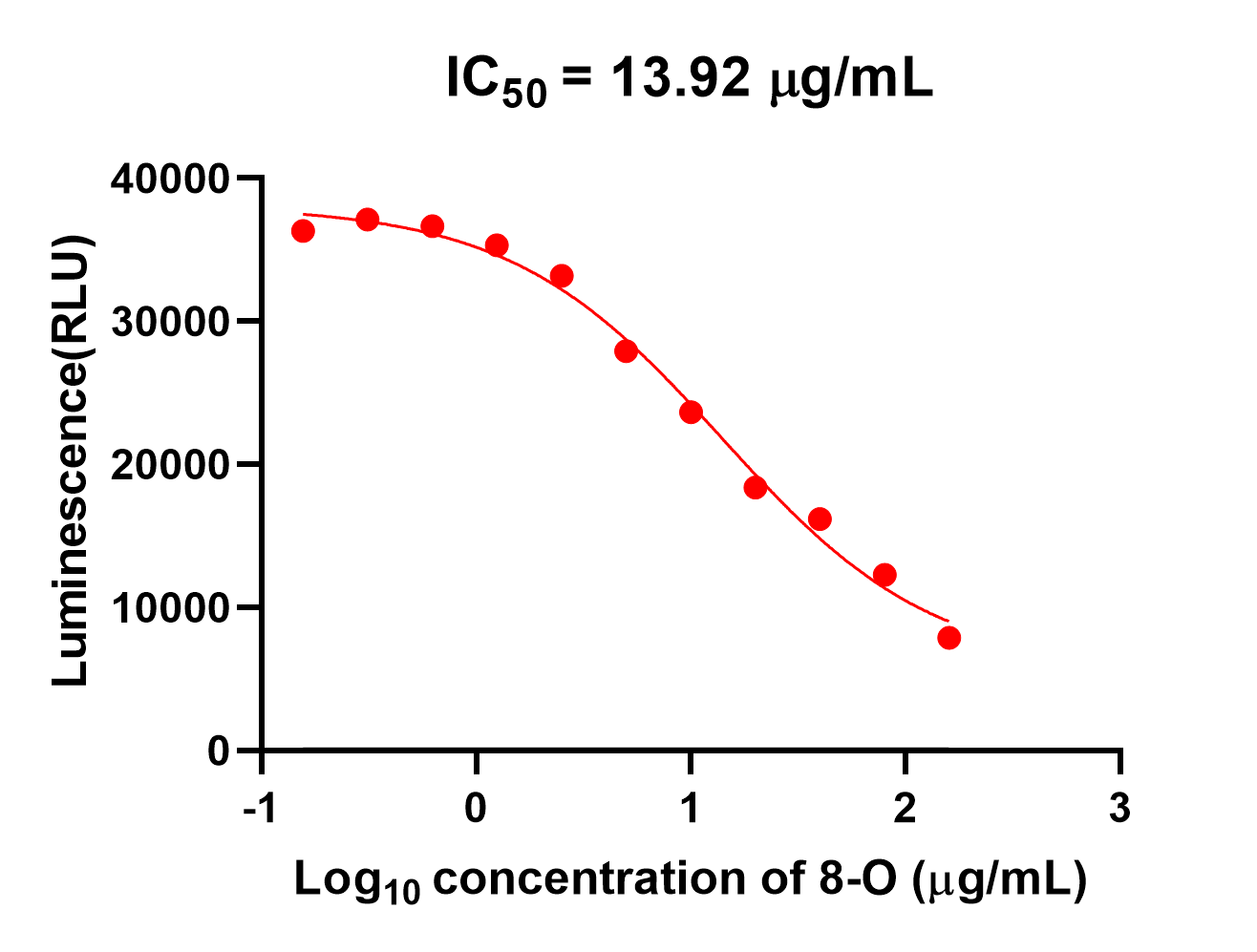


**Figure. S10**.

**Inhibition of 8-O on the ATPase activity of SecA.**

**Supplementary Figure 11**


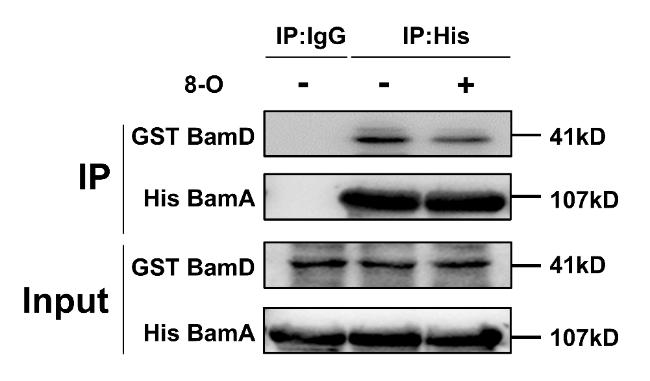


**Figure. S11**.

**The interaction of BamA and BamD was inhibited by 8-O in Co-IP analysis.**

**Supplementary Figure 12**


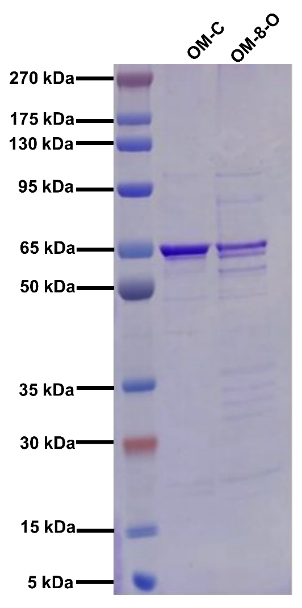


**Figure. S12**.

**The change of the total amount of *H. pylori* OMPs after the treatment of 8-O. OM-C, untreated control; OM-8-O, treated by 8-O.**

**Table S1.**

Cytotoxicity of **5**, **7** and **8** in four different cells (TC_50_, μM).

| **Code** | **5** | **7** | **8** |
| --- | --- | --- | --- |
| GES-1 | 24.96 | 8.81 | 54.20 |
| HepG2 | 23.67 | 10.38 | 50.59 |
| H460 | 19.21 | 12.70 | 57.07 |
| 293T | 17.68 | 9.13 | 53.80 |

**Table S2.**

Concentrations of **8** in mouse stomachs at different time points following a single oral dose of 30 mg/kg.

| **Time (h)** | **Concentration (μg/g)** | | | | **Mean** | **SD** |
| --- | --- | --- | --- | --- | --- | --- |
| **0.5** | 64.2 | 62.7 | 66.1 | 69.2 | 65.55 | 2.80 |
| **1** | 72.6 | 67.0 | 54.8 | 74.9 | 67.33 | 8.98 |
| **2** | 51.4 | 62.4 | 51.8 | 70.2 | 58.95 | 9.07 |
| **4** | 45.8 | 50.7 | 56.8 | 68.8 | 55.53 | 9.93 |
| **6** | 31.2 | 37.2 | 43.1 | 28.5 | 35.00 | 6.51 |
| **8** | 14.5 | 10.3 | 19.3 | 18.9 | 15.76 | 4.25 |
| **12** | 8.4 | 21.6 | 19.9 | 14.8 | 16.17 | 5.94 |
| **24** | 4.9 | 0.8 | 3.6 | 3.8 | 3.25 | 1.74 |

**Table S3.**

Concentrations of **8** in mouse plasma at different time points following a single oral dose of 30 mg/kg.

| **Time (h)** | **Concentration (μg/mL)** | | | | **Mean** | **SD** |
| --- | --- | --- | --- | --- | --- | --- |
| **0.5** | 0.0900 | 0.1000 | 0.0778 | 0.0938 | 0.0904 | 0.0094 |
| **1** | 0.0379 | 0.0122 | 0.0214 | 0.0434 | 0.0287 | 0.0144 |
| **2** | 0.0148 | 0.0054 | 0.0201 | 0.0193 | 0.0149 | 0.0067 |
| **4** | 0.0053 | 0.0014 | 0.0034 | 0.0029 | 0.0033 | 0.0016 |
| **6** | BQL^a^ | BQL | BQL | BQL | BQL | BQL |
| **8** | BQL | BQL | 0.0013 | BQL | BQL | BQL |
| **12** | BQL | BQL | BQL | BQL | BQL | BQL |
| **24** | BQL | BQL | BQL | BQL | BQL | BQL |

^a^ Below the Lower Limit of Quantitation (LLOQ) of 0.001 μg/mL.

**Table S4.**

Acid stability of **8** in different conditions.

| **Time points** | **Relative content ^a^**  **(%, pH = 1.0)** | **Relative content ^a^**  **(%, pH = 3.0)** |
| --- | --- | --- |
| 2 h | 95.0 | 91.1 |
| 8 h | 90.9 | 93.8 |
| 24 h | 91.4 | 96.1 |

^a^ The relative content percentage of each sample at different conditions and time points is calculated based on the peak area of compound **8** without acid treatment at the same concentration, which was set as 100% in HPLC.

HPLC conditions are as follows: the mobile phase is acetonitrile (chromatographically pure) and KH_2_PO_4_ (pH = 2.8), the ratio of acetonitrile to KH_2_PO_4_ is 75%:25%, isocratic elution, the flow rate is 1 mL/min. The injection volume is 5 μL. The column temperature is 30 °C; the detection wavelength of the detector is 254 nm.

**Table S5.**

Combination effect of **8** and CLA against 25 *H. pylori* strains in checkerboard assay.

| Strain | MIC^a^ (μg/mL) alone | | MIC^a^ (μg/mL) combined | | FICI^b^ | Combination  Effects |
| --- | --- | --- | --- | --- | --- | --- |
|  | **8** | CLA^c^ | **8** | CLA^c^ |  |  |
| CCPM(A)-P-372302 | 1 | 0.031 | 0.25 | 0.016 | 0.750 | Additive |
| CCPM(A)-P-372303 | 0.25 | 16 | 0.063 | 1 | 0.313 | Synergistic |
| CCPM(A)-P-372304 | 1 | 16 | 1 | 1 | 1.063 | Additive |
| CCPM(A)-P-372315 | 1 | 16 | 0.125 | 2 | 0.250 | Synergistic |
| CCPM(A)-P-372316 | 0.5 | 16 | 0.5 | 0.004 | 1.000 | Additive |
| CCPM(A)-P-372317 | 1 | 16 | 1 | 0.008 | 1.000 | Additive |
| CCPM(A)-P-372320 | 0.25 | 0.015 | 0.063 | 0.004 | 0.510 | Additive |
| CCPM(A)-P-372321 | 1 | 0.063 | 0.063 | 0.015 | 0.303 | Synergistic |
| CCPM(A)-P-372322 | 1 | 0.063 | 0.5 | 0.015 | 0.740 | Additive |
| CCPM(A)-P-372323 | 1 | 0.031 | 0.125 | 0.008 | 0.375 | Synergistic |
| CCPM(A)-P-372324 | 0.5 | 0.063 | 0.25 | 0.008 | 0.625 | Additive |
| CCPM(A)-P-372325 | 1 | 0.063 | 0.5 | 0.004 | 0.563 | Additive |
| CCPM(A)-P-372326 | 0.5 | 0.063 | 0.25 | 0.008 | 0.625 | Additive |
| CCPM(A)-P-372327 | 1 | 0.031 | 0.25 | 0.008 | 0.500 | Synergistic |
| CCPM(A)-P-372329 | 1 | 0.031 | 0.25 | 0.008 | 0.500 | Synergistic |
| CCPM(A)-P-372330 | 0.5 | 0.031 | 0.5 | 0.004 | 1.125 | Additive |
| CCPM(A)-P-372331 | 1 | 0.060 | 0.5 | 0.004 | 0.565 | Additive |
| CCPM(A)-P-372332 | 0.5 | 8 | 0.063 | 0.500 | 0.188 | Synergistic |
| CCPM(A)-P-372334 | 0.5 | 16 | 0.063 | 4 | 0.375 | Synergistic |
| CCPM(A)-P-372335 | 1 | 0.031 | 0.5 | 0.008 | 0.750 | Additive |
| CCPM(A)-P-372336 | 1 | 16 | 0.5 | 4 | 0.750 | Additive |
| ATCC 43504 | 1 | 0.031 | 0.5 | 0.004 | 0.625 | Additive |
| ATCC 700392 | 1 | 0.031 | 0.5 | 0.016 | 1.000 | Additive |
| SS1 | 0.5 | 0.031 | 0.063 | 0.008 | 0.375 | Synergistic |
| CCPM(A)-P-3722159 | 0.5 | 16 | 0.125 | 4 | 0.500 | Synergistic |

^a^MIC measurements were repeated three times with identical results. ^b^FICI ≤ 0.5, synergistic; 0.5 < FICI ≤ 4, additive; FICI > 4, antagonistic. ^c^CLA-resistant strains: MIC breakpoints of CLA for *H. pylori*: ≤ 0.25 μg/mL for susceptible, and > 0.25 μg/mL for resistant, according to EUCAST.

**Table S6.**

Combination effect of **8** and AMX against 25 *H. pylori* strains in checkerboard assay.

| Strain | MIC^a^ (μg/mL) alone | | MIC^a^ (μg/mL) combined | | FICI^b^ | Combination  Effects |
| --- | --- | --- | --- | --- | --- | --- |
|  | **8** | AMX^c^ | **8** | AMX^c^ |  |  |
| CCPM(A)-P-372302 | 1 | 0.0078 | 0.5 | 0.0039 | 1.000 | Additive |
| CCPM(A)-P-372303 | 0.25 | 0.0625 | 0.063 | 0.0313 | 0.750 | Additive |
| CCPM(A)-P-372304 | 1 | 0.0156 | 1 | 0.0005 | 1.031 | Additive |
| CCPM(A)-P-372315 | 1 | 0.25 | 0.063 | 0.125 | 0.563 | Additive |
| CCPM(A)-P-372316 | 0.5 | 0.0156 | 0.500 | 0.0005 | 1.031 | Additive |
| CCPM(A)-P-372317 | 1 | 0.0078 | 0.25 | 0.0039 | 0.750 | Additive |
| CCPM(A)-P-372320 | 0.25 | 0.0020 | 0.125 | 0.0010 | 1.000 | Additive |
| CCPM(A)-P-372321 | 1 | 0.0313 | 0.125 | 0.0156 | 0.625 | Additive |
| CCPM(A)-P-372322 | 1 | 0.0625 | 0.5 | 0.0313 | 1.000 | Additive |
| CCPM(A)-P-372323 | 1 | 0.0313 | 0.125 | 0.0156 | 0.625 | Additive |
| CCPM(A)-P-372324 | 0.5 | 0.0078 | 0.063 | 0.0039 | 0.625 | Additive |
| CCPM(A)-P-372325 | 1 | 0.0078 | 0.25 | 0.0039 | 0.750 | Additive |
| CCPM(A)-P-372326 | 1 | 0.0020 | 0.063 | 0.0020 | 1.063 | Additive |
| CCPM(A)-P-372327 | 1 | 0.0078 | 0.5 | 0.0039 | 1.000 | Additive |
| CCPM(A)-P-372329 | 1 | 0.0625 | 0.5 | 0.0313 | 1.000 | Additive |
| CCPM(A)-P-372330 | 0.5 | 0.0313 | 0.5 | 0.0005 | 1.016 | Additive |
| CCPM(A)-P-372331 | 1 | 0.0078 | 0.25 | 0.0039 | 0.750 | Additive |
| CCPM(A)-P-372332 | 0.5 | 0.0020 | 0.125 | 0.0010 | 0.750 | Additive |
| CCPM(A)-P-372334 | 0.5 | 0.125 | 0.125 | 0.0625 | 0.750 | Additive |
| CCPM(A)-P-372335 | 1 | 0.0078 | 0.125 | 0.0039 | 0.625 | Additive |
| CCPM(A)-P-372336 | 1 | 0.0313 | 0.5 | 0.0156 | 1.000 | Additive |
| ATCC 43504 | 1 | 0.0156 | 1 | 0.0005 | 1.031 | Additive |
| ATCC 700392 | 1 | 0.0313 | 1 | 0.0005 | 1.016 | Additive |
| SS1 | 0.25 | 0.25 | 0.063 | 0.0625 | 0.500 | Additive |
| CCPM(A)-P-3722159 | 1 | 0.25 | 0.125 | 0.125 | 0.625 | Additive |

^a^MIC measurements were repeated three times with identical results. ^b^FICI ≤ 0.5, synergistic; 0.5 < FICI ≤ 4, additive; FICI > 4, antagonistic. ^c^AMX-resistant strains: MIC breakpoints of AMX for *H. pylori*: ≤ 0.125μg/mL for susceptible, and > 0.125 μg/mL for resistant, according to EUCAST.

**Table S7.**

The antibacterial spectrum of compound **8**.

| **Strains** | **Gram stain** | **MIC (μg/mL)** |
| --- | --- | --- |
| ***Staphylococcus aureus* ATCC 43300** | **G+** | 64 |
| ***Staphylococcus aureus* ATCC 700698** | **G+** | >128 |
| ***Staphylococcus aureus* ATCC 29213** | **G+** | 8 |
| ***Enterococcus faecalis* ATCC 29212** | **G+** | 64 |
| ***Enterococcus faecalis* ATCC 51299** | **G+** | 128 |
| ***Enterococcus faecalis* ATCC 51575** | **G+** | 128 |
| ***Enterococcus faecalis* 20-2** | **G+** | 64 |
| ***Enterococcus faecium* ATCC 700221** | **G+** | 32 |
| ***Enterococcus faecium* 20-2** | **G+** | >128 |
| ***Enterococcus faecium* 15-6** | **G+** | >128 |
| ***Escherichia coli* ATCC 25922** | **G-** | >128 |
| ***Escherichia coli* ATCC 35218** | **G-** | >128 |
| ***Escherichia coli* ATCC 2469** | **G-** | >128 |
| ***Escherichia coli* 21-2** | **G-** | 64 |
| ***Escherichia coli* 21-3** | **G-** | 64 |
| ***Klebsiella pneumoniae* ATCC 700603** | **G-** | >128 |
| ***Klebsiella pneumoniae* ATCC 2146** | **G-** | >128 |
| ***Klebsiella pneumoniae* 7** | **G-** | >128 |
| ***Klebsiella pneumoniae* 21-16** | **G-** | >128 |
| ***Klebsiella pneumoniae* 21-18** | **G-** | >128 |
| ***Pseudomonas aeruginosa* ATCC 27853** | **G-** | >128 |
| ***Pseudomonas aeruginosa* PAO1** | **G-** | >128 |
| ***Acinetobacter baumannii* ATCC 19606** | **G-** | >128 |
| ***Acinetobacter baumannii* 16-33** | **G-** | >128 |
| ***Enterobacter cloacae* ATCC 23560** | **G-** | >128 |
| ***Klebsiella aerogenes* ATCC 13048** | **G-** | >128 |
| ***Serratia marcescens* ATCC 21074** | **G-** | >128 |
| ***Proteus mirabilis* ATCC 49565** | **G-** | >128 |
| ***Stenotrophomonas maltophilia* ATCC 13636** | **G-** | >128 |
| ***Shigella flexneri* ATCC 12022** | **G-** | >128 |

**Supplementary Table S8.**

Activities of the probe **8-O** against *H. pylori* strains (MIC, μg/mL).

| ***H.pylori* strains** | **ATCC**  **43504** | **ATCC**  **700392** | **CCPM(A)-P-3716280** | **CCPM(A)-P-3716289** | **CCPM(A)-P-3716370** |
| --- | --- | --- | --- | --- | --- |
| **8-O** | 1 | 0.5 | 1 | 1 | 2 |

**Table S9.**

The potential targets (identified twice in three biological replicates) of **8** identified by LC/MS-MS.

| **Protein ID** | **Protein Name** | **Coverage [%]** | **Unique Peptides** | **MW [kDa]** | **Sum PEP Score** |
| --- | --- | --- | --- | --- | --- |
| A0A2X4ZUA0 | Nucleoside diphosphate kinase | 31 | 4 | 15.3 | 16.682 |
| A0A2X5A961 | Lipopolysaccharide heptosyltransferase-1 | 10 | 4 | 38.1 | 8.706 |
| I0ZFF6 | 50S ribosomal protein L29 | 21 | 1 | 7.7 | 5.411 |
| A0A2X4ZXL1 | Glyceraldehyde-3-phosphate dehydrogenase | 13 | 4 | 37.1 | 17.664 |
| A0A2X4ZMX9 | Urease accessory protein UreE | 33 | 6 | 19.4 | 30.074 |
| A0A2X4ZSS9 | Potassium channel protein | 8 | 3 | 42.8 | 14.02 |
| A0A2X5DNQ6 | Chemotaxis protein CheW | 34 | 4 | 19 | 15.4 |
| I0ZHZ3 | Outer membrane protein assembly factor BamD | 16 | 3 | 26.2 | 8.519 |
| A0A2X5AIW3 | Uncharacterized protein | 12 | 4 | 39.5 | 12.135 |
| A0A2X4ZSP9 | Glutathione hydrolase proenzyme | 16 | 7 | 61.1 | 35.457 |
| A0A2X5A4Z2 | Glycine--tRNA ligase beta subunit | 22 | 15 | 80.3 | 60.119 |
| A0A2X5A450 | Cytochrome c551 peroxidase | 35 | 12 | 38.8 | 50.613 |
| A0A2X5A947 | Mechanosensitive ion channel family protein | 13 | 7 | 60 | 27.38 |
| A0A2X5CRH1 | Outer membrane protein | 35 | 11 | 55 | 93.63 |
| A0A2X5ABQ6 | Cag pathogenicity island protein (Cag1) | 30 | 3 | 12.4 | 31.606 |
| A0A2X5ATN0 | Carboxyl-terminal protease | 38 | 4 | 50 | 78.519 |
| A0A2X5AGS7 | Acetyl-coenzyme A synthetase | 41 | 27 | 75 | 153.357 |
| A0A2X4ZRL9 | NADH-quinone oxidoreductase subunit I | 40 | 9 | 24.7 | 38.474 |
| A0A2X5A0P5 | Protein translocase subunit SecA | 29 | 23 | 99.1 | 100.12 |
| A0A2X5CH13 | Bifunctional protein PutA | 44 | 13 | 135 | 298.288 |
| A0A2X5AQH1 | Spore coat polysaccharide biosynthesis protein C | 10 | 4 | 42.3 | 13.06 |
| A0A2X5AFT0 | Diaminopimelate decarboxylase | 16 | 7 | 45.1 | 20.686 |
| A0A2X5AG39 | CopG family transcriptional regulator | 28 | 2 | 9.2 | 4.225 |
| A0A2X5A5C6 | DL-methionine transporter ATP-binding subunit | 9 | 3 | 36.6 | 10.244 |

**Table S10.**

Activities of CJ-21058 against *H. pylori* strains (MIC, μg/mL).

| ***H.pylori* strains** | **ATCC**  **43504** | **ATCC**  **700392** | **CCPM(A)-**  **P-3716280** | **CCPM(A)-**  **P-3716289** | **CCPM(A)-**  **P-3716370** | **SS1** |
| --- | --- | --- | --- | --- | --- | --- |
| **CJ-21058** | 4 | 4 | 8 | 8 | 4 | 8 |

**Table S11.**

The qRT-PCR primers used in this study.

| **Primers** |  | **Sequence (5’**–**3’)** |
| --- | --- | --- |
| *babA* | Forward | GGTGGTCCACAGATGGAACC |
|  | Reverse | GATTGACCAGCTCTTGTGCG |
| *sabA* | Forward | GGCTACCCCACTCAATACGC |
|  | Reverse | ATCCTGTGGCTTGAGCTTGC |
| *oipA* | Forward | ACCGATTCGCAGGAAATGGT |
|  | Reverse | GAACCAACGCCACCAAGTTG |
| *16srRNA* | Forward | GTGCCAGCMGCCGCGGTAA |
|  | Reverse | GACTACHVGGGTATCTAATCC |

**Characterization Data of Deep Learning Predicted Compounds**

Compound **1**, a turmeric powder with a melting point of 212–214 °C, was synthesized according to the **synthetic procedure**. This reaction provided the desired compound in 76% yield.

**^1^H NMR** (600 MHz, DMSO-*d*_6_): δ 9.98 (s, 1H), 9.24 (s, 1H), 8.26 (d, *J* = 9.1 Hz, 1H), 8.10 (d, *J* = 9.1 Hz, 1H), 7.92 (s, 1H), 7.29 (s, 1H), 4.99 (t, *J* = 6.3 Hz, 2H), 4.12 (s, 3H), 4.09 (s, 3H), 3.97 (s, 3H), 3.23 (t, *J* = 6.3 Hz, 2H), 1.95 (tt, *J* = 8.2, 4.6 Hz, 1H), 1.17–1.09 (m, 2H), 1.07–0.90 (m, 2H).

**^13^C NMR** (151 MHz, DMSO-*d*_6_): δ 172.1, 151.0, 150.8, 145.9, 143.8, 141.5, 136.8, 132.7, 127.9, 126.8, 125.4, 123.7, 122.9, 121.7, 121.4, 110.1, 62.0, 57.1, 56.6, 55.3, 25.4, 12.4, 9.1.

**HRMS (ESI)** *m/z* calc for C_24_H_24_NO_5_Cl^+^ [M–Cl]^+^ : 406.16490, found: 406.16463.

^1^H NMR


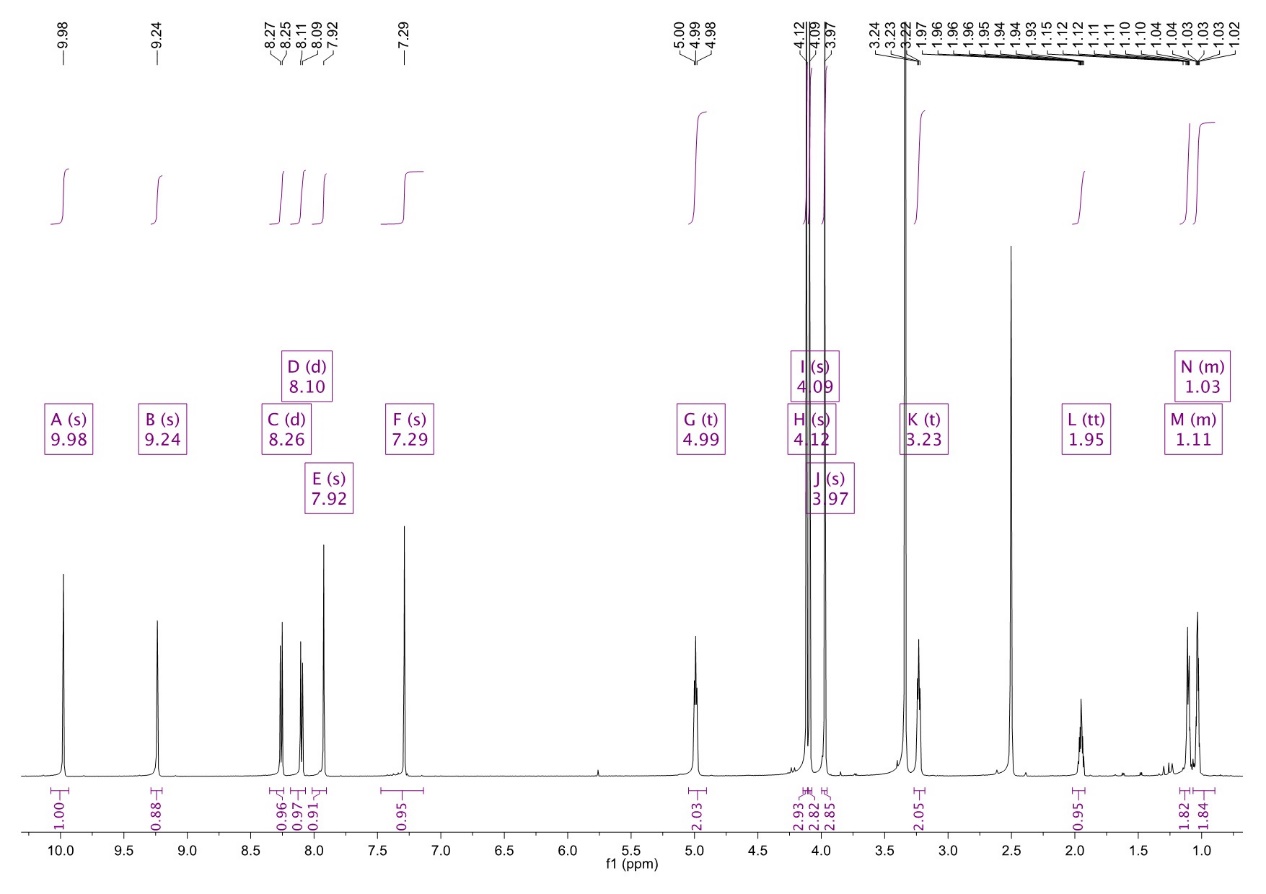


^13^C NMR


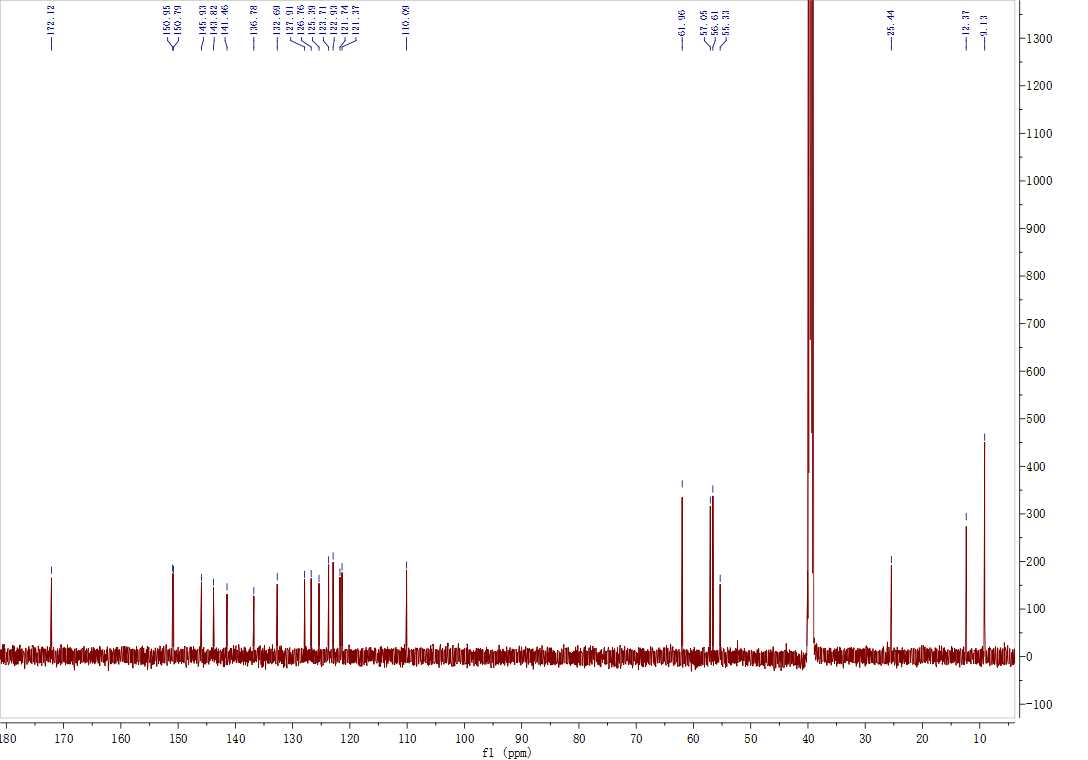


HRMS (ESI)


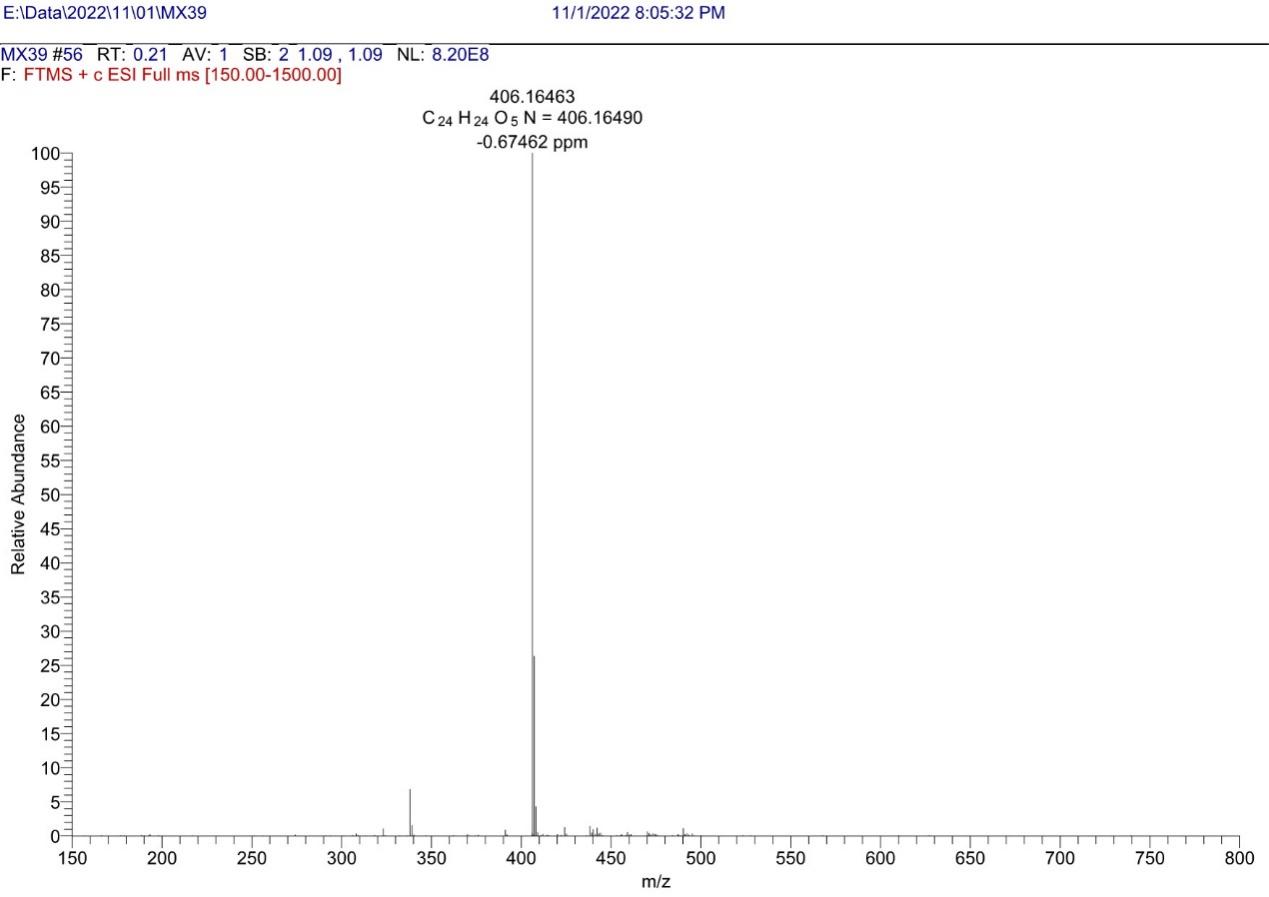

Compound **2**, a turmeric powder with a melting point of 208–210 °C, was synthesized according to the **synthetic procedure**. This reaction provided the desired compound in 72% yield.

**^1^H NMR** (600 MHz, DMSO-*d*_6_): δ 9.98 (s, 1H), 9.26 (s, 1H), 8.26 (d, *J* = 9.1 Hz, 1H), 8.11 (d, *J* = 9.0 Hz, 1H), 7.94 (s, 1H), 7.28 (s, 1H), 5.00 (t, *J* = 6.3 Hz, 2H), 4.12 (s, 3H), 4.09 (s, 3H), 3.97 (s, 3H), 3.53–3.47 (m, 1H), 3.24 (t, *J* = 6.3 Hz, 2H), 2.39–2.29 (m, 4H), 2.03 (dp, *J* = 11.0, 8.7 Hz, 1H), 1.96–1.88 (m, 1H).

**^13^C NMR** (151 MHz, DMSO-*d*_6_): δ 172.6, 150.9, 150.8, 145.9, 143.8, 141.6, 136.8, 132.7, 128.0, 126.8, 125.4, 123.7, 122.8, 121.7, 121.4, 110.1, 62.0, 57.1, 56.7, 55.3, 36.9, 25.4, 24.7, 17.9.

**HRMS (ESI)** *m/z* calc for C_25_H_26_NO_5_Cl^+^ [M–Cl]^+^: 420.18055, found: 420.18085.

^1^H NMR


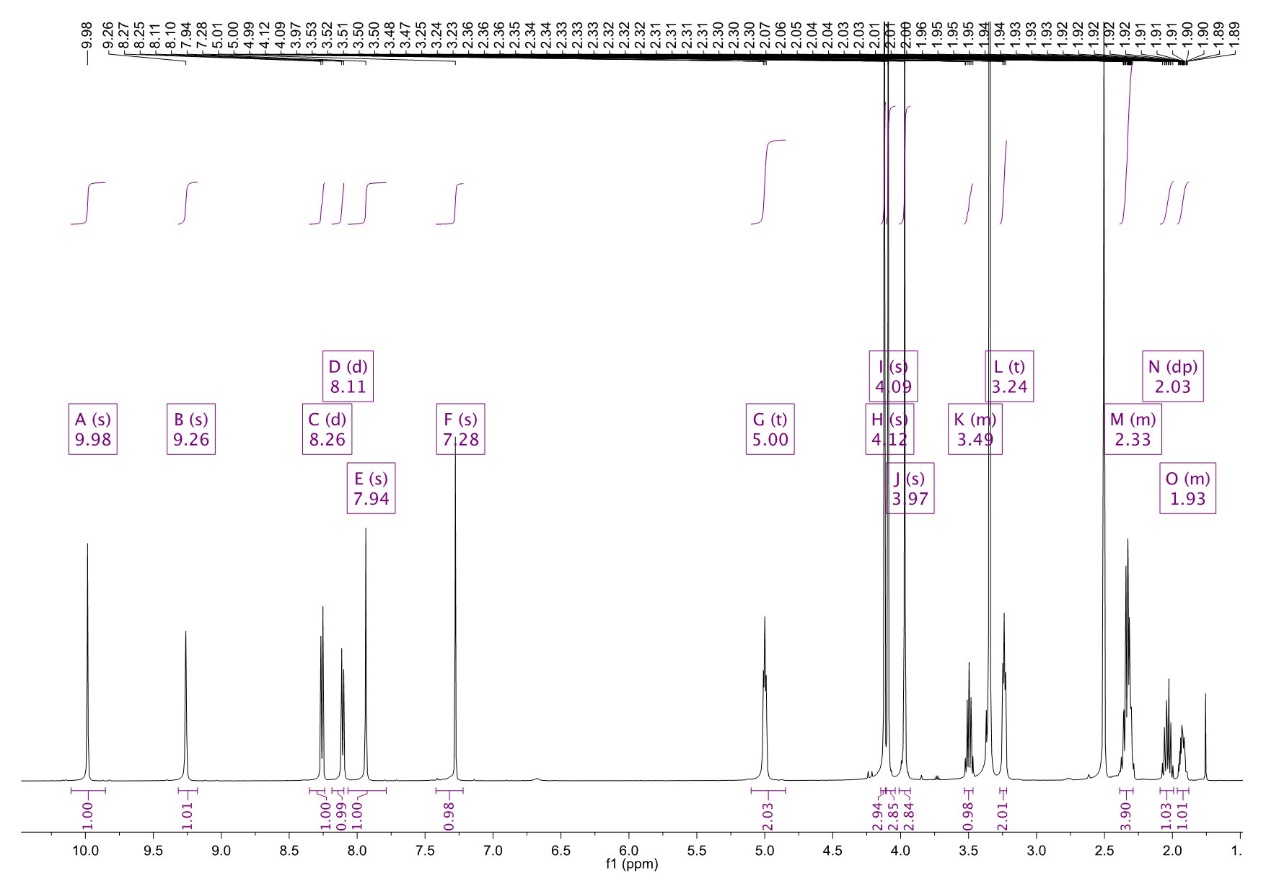


^13^C NMR


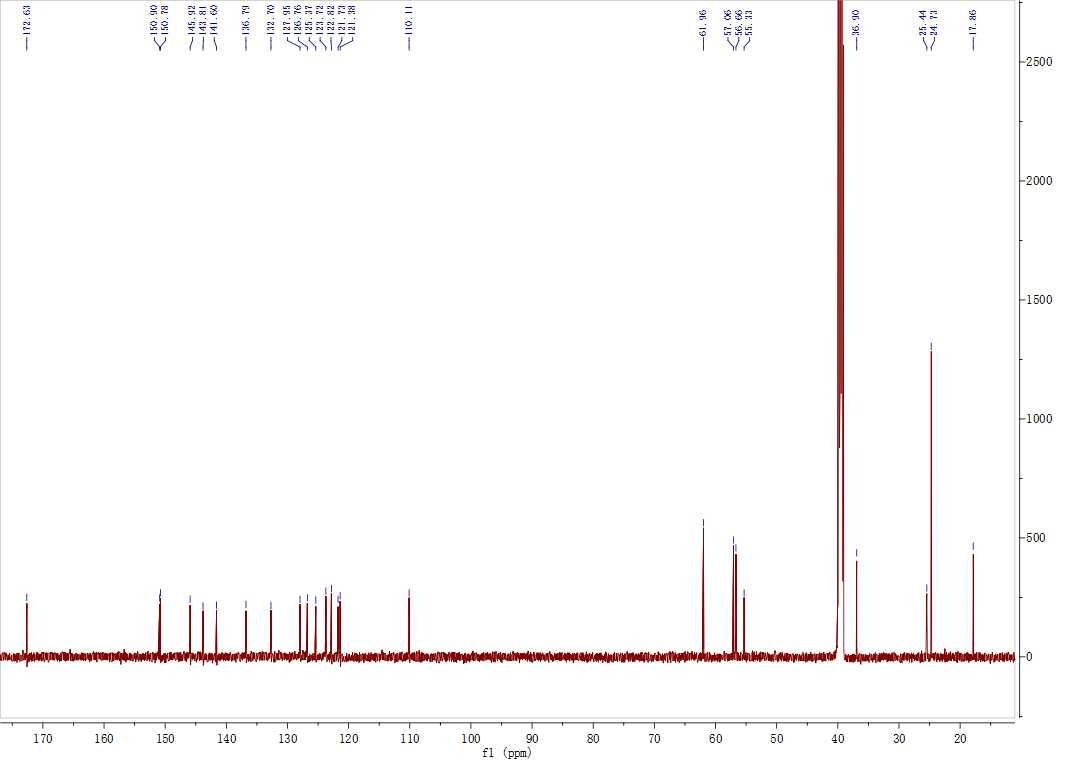


HRMS (ESI)


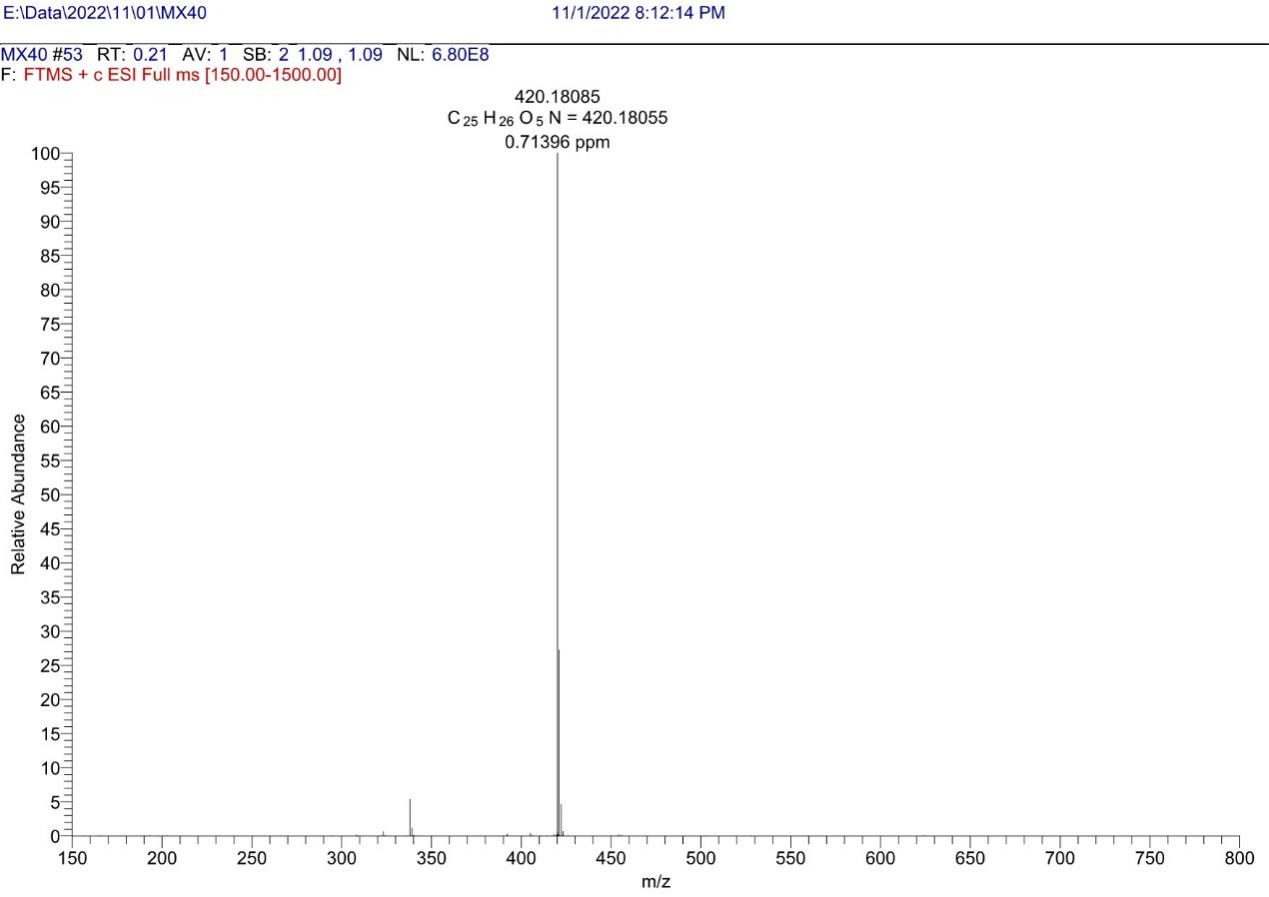


Compound **3**, a turmeric powder with a melting point of 202–204 °C, was synthesized according to the **synthetic procedure**. This reaction provided the desired compound in 80% yield.

**^1^H NMR** (600 MHz, DMSO-*d*_6_): δ 9.89 (s, 1H), 9.04 (s, 1H), 8.22 (d, *J* = 9.1 Hz, 1H), 8.04 (d, *J* = 9.1 Hz, 1H), 7.74 (s, 1H), 7.49 (d, *J* = 7.0 Hz, 2H), 7.46–7.41 (m, 2H), 7.40–7.35 (m, 1H), 7.22 (s, 1H), 5.21 (s, 2H), 4.95 (t, *J* = 6.3 Hz, 2H), 4.10 (s, 3H), 4.08 (s, 3H), 3.95 (s, 3H), 3.21 (t, *J* = 6.4 Hz, 2H).

**^13^C NMR** (151 MHz, DMSO-*d*_6_): δ 173.9, 150.9, 150.8, 145.9, 143.8, 141.7, 136.8, 132.7, 127.9, 126.7, 125.3, 123.8, 122.8, 121.7, 121.4, 110.2, 62.0, 57.1, 56.7, 55.3, 42.7, 29.5, 29.0, 25.4.

**HRMS (ESI)** *m/z* calc for C_26_H_28_NO_5_Cl^+^ [M–Cl]^+^: 434.19620, found: 434.19555.

^1^H NMR


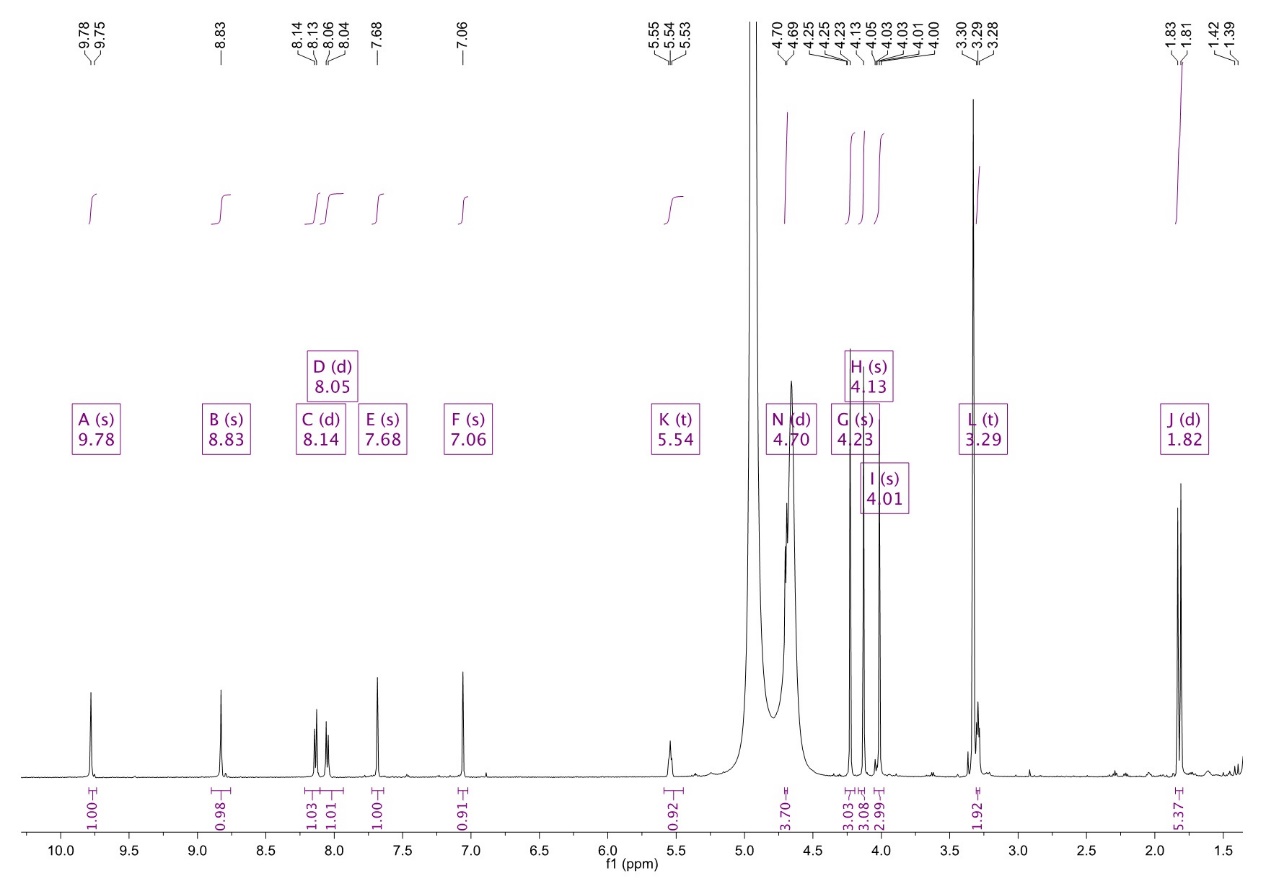


^13^C NMR


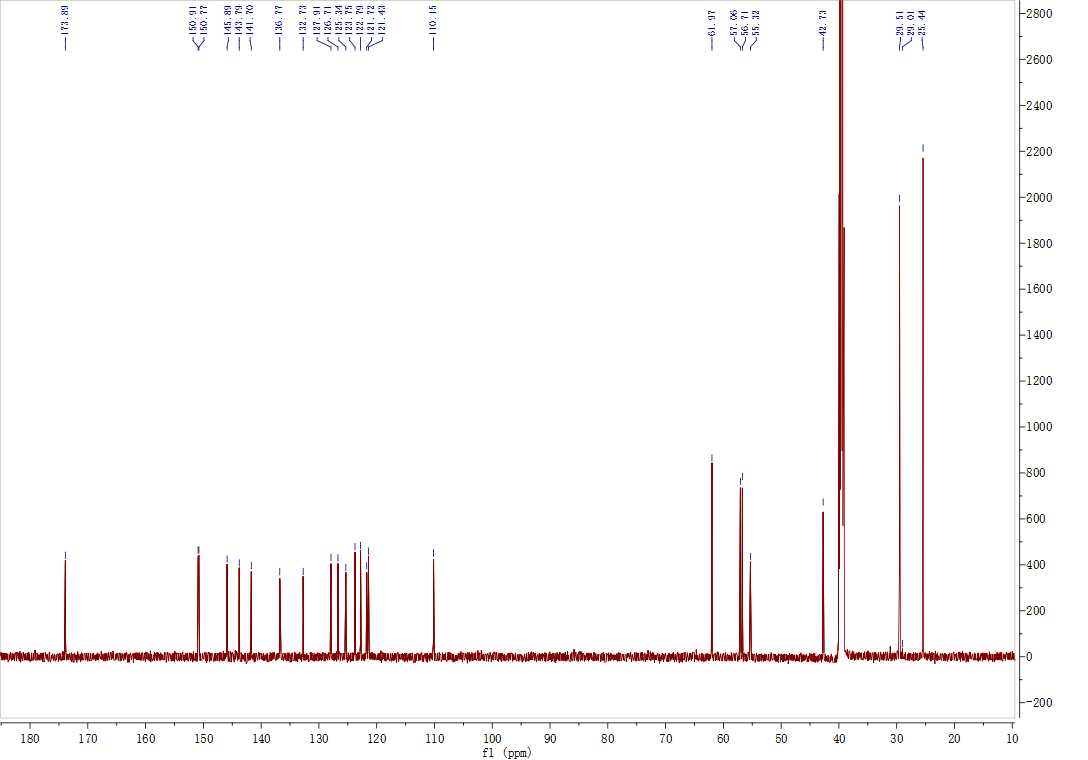


HRMS (ESI)


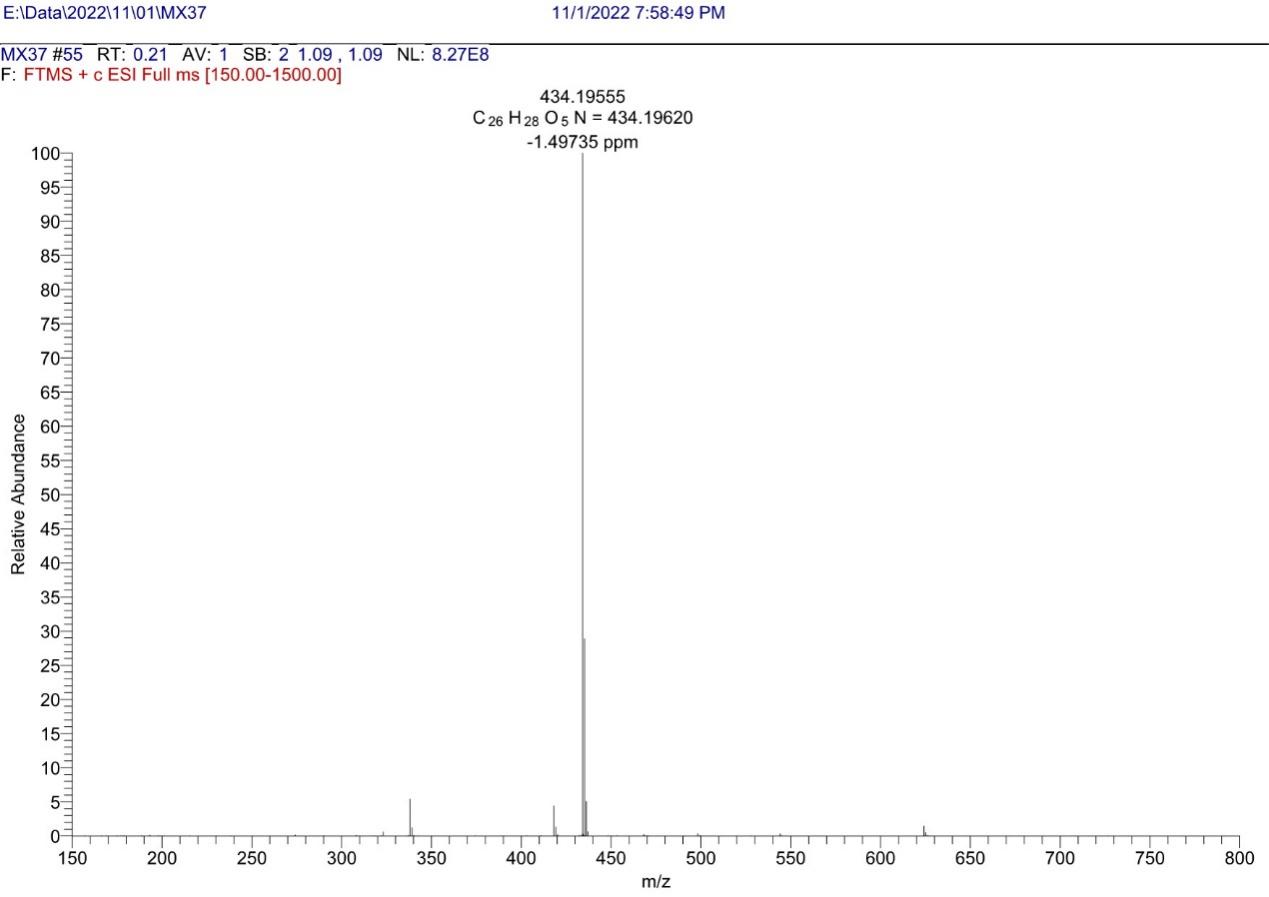

Compound **4**, a turmeric powder with a melting point of 215–217 °C, was synthesized according to the **synthetic procedure**. This reaction provided the desired compound in 80% yield.

**^1^H NMR** (600 MHz, DMSO-*d*_6_): δ 9.98 (s, 1H), 9.29–9.22 (m, 1H), 8.26 (d, *J* = 9.2 Hz, 1H), 8.10 (d, *J* = 9.1 Hz, 1H), 7.93 (s, 1H), 7.26 (s, 1H), 4.99 (t, *J* = 6.4 Hz, 2H), 4.12 (s, 3H), 4.09 (s, 3H), 3.96 (s, 3H), 3.24 (t, *J* = 6.4 Hz, 2H), 2.61 (d, *J* = 7.4 Hz, 2H), 2.27 (p, *J* = 7.7 Hz, 1H), 1.90–1.83 (m, 2H), 1.65 (tdd, *J* = 12.5, 11.2, 9.5, 4.7 Hz, 2H), 1.58–1.54 (m, 2H), 1.26 (dddd, *J* = 16.0, 13.0, 6.5, 2.4 Hz, 2H).

**^13^C NMR** (151 MHz, DMSO-*d*_6_): δ 170.4, 150.9, 150.8, 145.9, 143.8, 141.6, 136.8, 132.7, 128.0, 126.8, 125.4, 123.7, 122.8, 121.7, 121.4, 110.1, 62.0, 57.1, 56.6, 55.3, 39.2, 36.1, 31.8, 25.4, 24.6.

**HRMS (ESI)** *m/z* calc for C_27_H_30_NO_5_Cl^+^ [M–Cl]^+^: 448.21185, found: 448.21125.

^1^H NMR


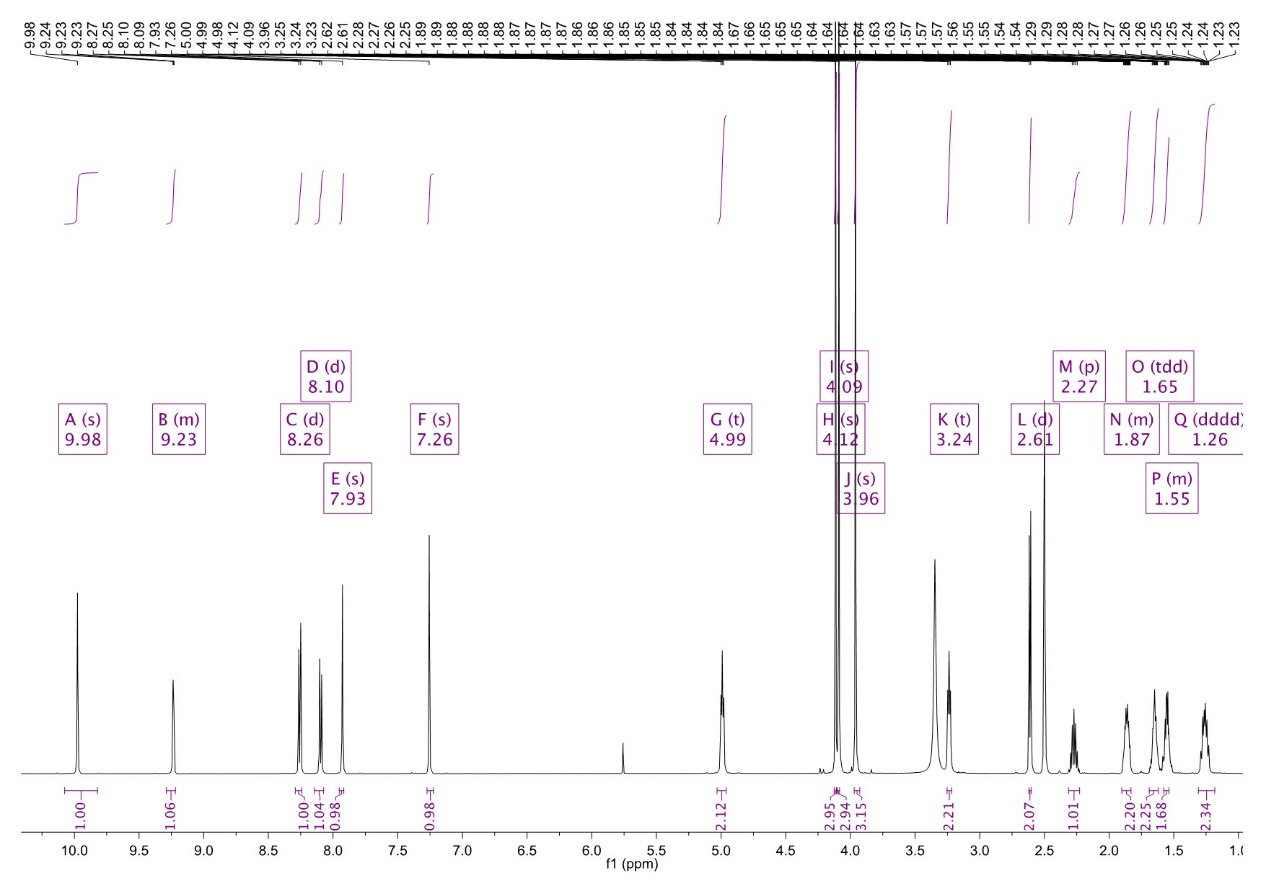


^13^C NMR


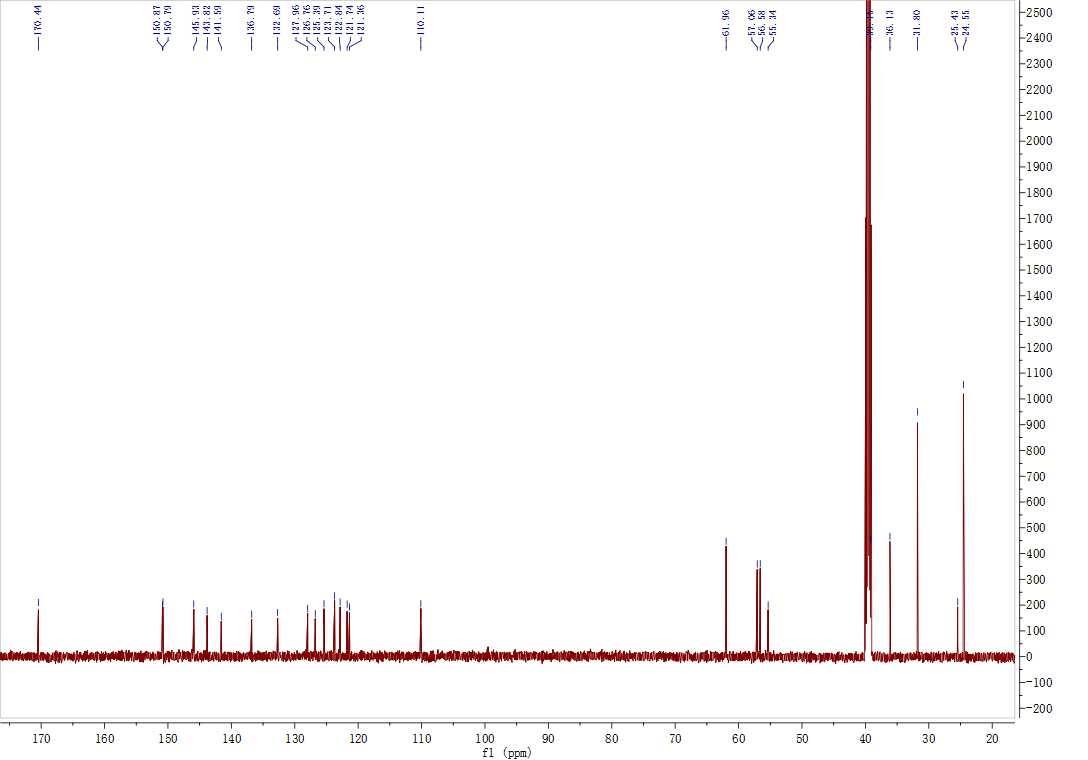


HRMS (ESI)


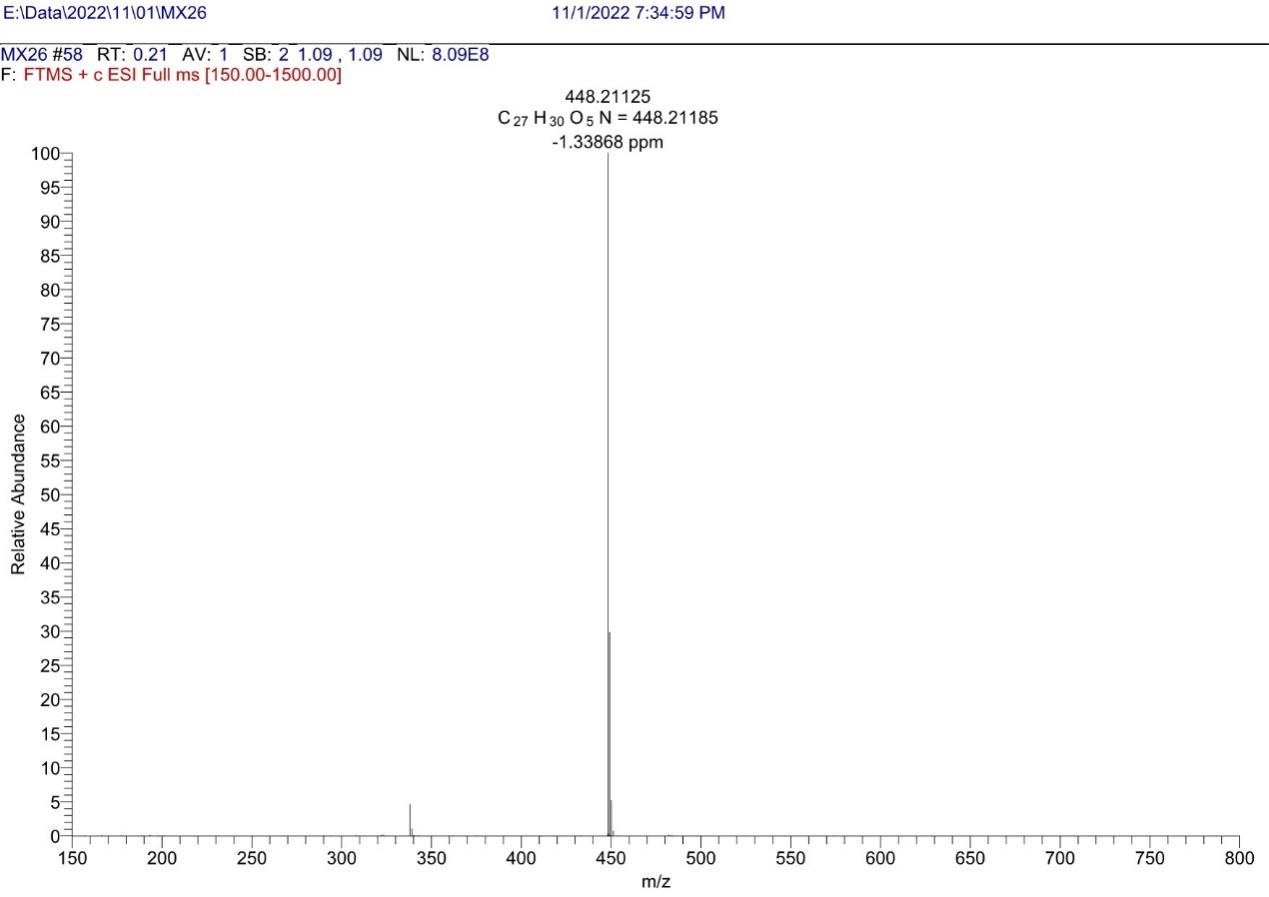


Compound **5**, a turmeric powder with a melting point of 209–211 °C, was synthesized according to the **synthetic procedure**. This reaction provided the desired compound in 77% yield.

**^1^H NMR** (600 MHz, MeOD): δ 9.74 (s, 1H), 8.79 (s, 1H), 8.10 (d, *J* = 9.0 Hz, 1H), 8.06–7.96 (m, 1H), 7.65 (s, 1H), 7.02 (s, 1H), 4.20 (s, 3H), 4.14 (t, *J* = 6.6 Hz, 3H), 4.10 (s, 3H), 4.00 (s, 3H), 3.35 (s, 1H), 3.27 (t, *J* = 6.4 Hz, 2H), 2.27 (dd, *J* = 14.1, 6.8 Hz, 1H), 2.10 (dq, *J* = 14.2, 7.2 Hz, 1H), 1.81 (dd, *J* = 10.7, 4.6 Hz, 4H), 1.74 (dd, *J* = 11.7, 4.9 Hz, 4H), 1.36–1.27 (m, 6H).

**^13^C NMR** (151 MHz, MeOD): δ 153.4, 151.9, 151.1, 146.3, 145.7, 139.8, 135.3, 130.0, 128.1, 124.5, 123.2, 121.2, 120.3, 113.2, 110.2, 68.3, 66.5, 62.6, 57.7, 57.1, 37.6, 35.8, 34.4, 34.3, 32.1, 27.6, 27.4, 21.5.

**HRMS (ESI)** *m/z* calc for C_28_H_34_NO_4_Br^+^ [M–Br]^+^: 448.24824, found: 448.24780.

**LCMS (254 nm):** *m/z* for C_28_H_34_NO_4_Br^+^ [M–Br]^+^: 448.2, 99.0% pure.

^1^H NMR


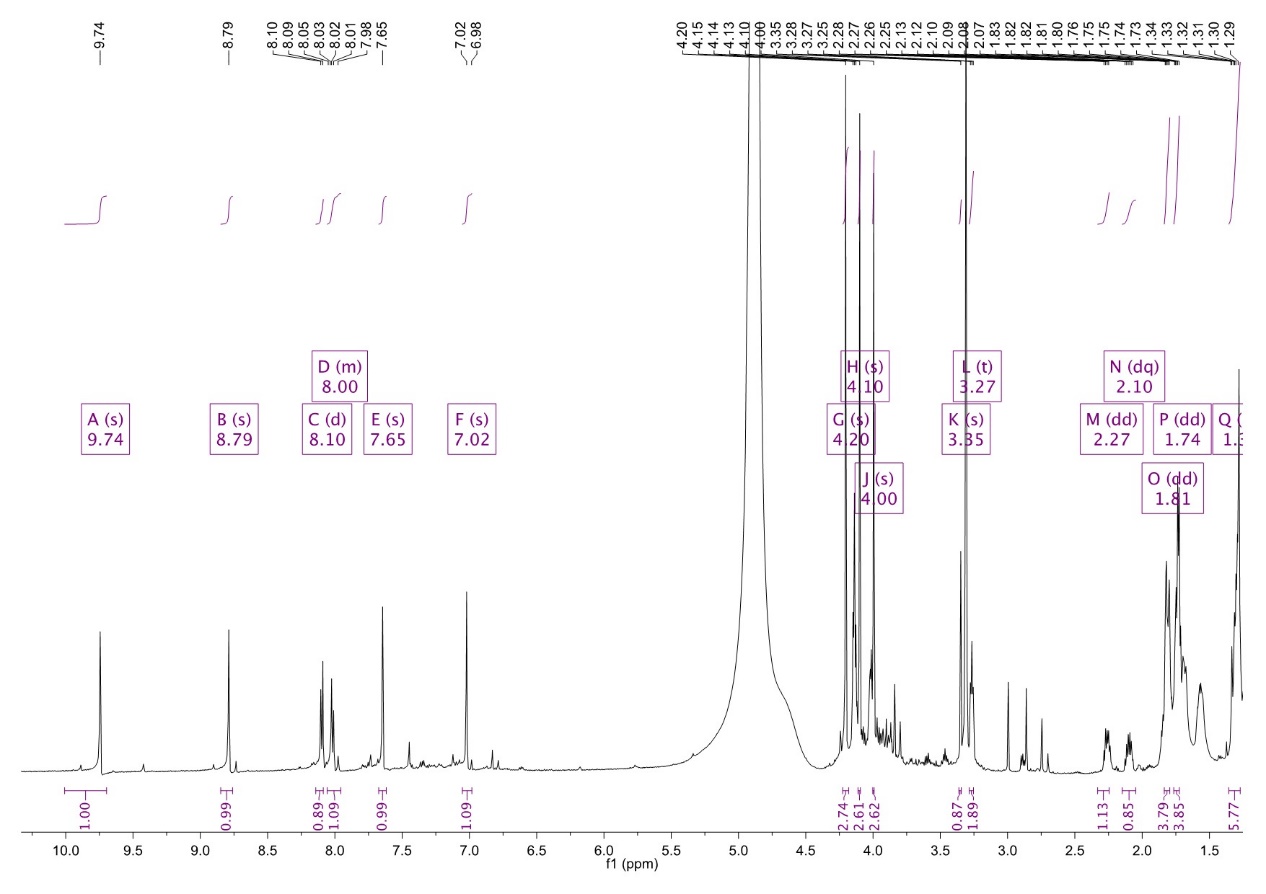


^13^C NMR


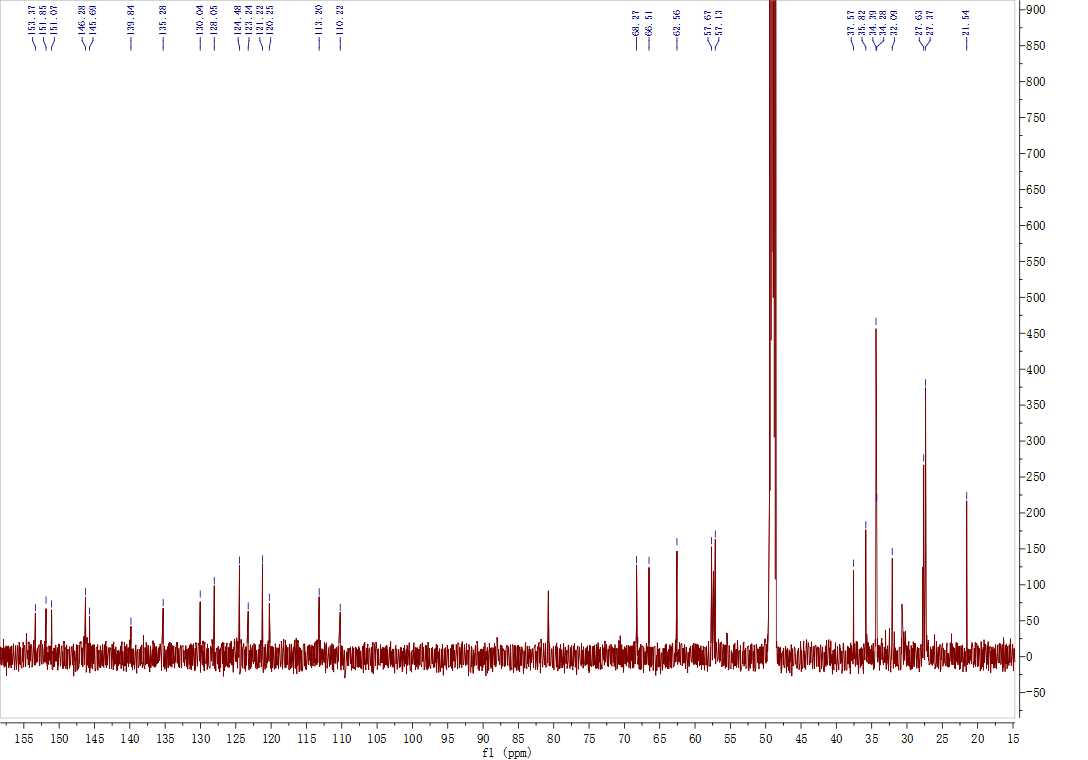


HRMS (ESI)


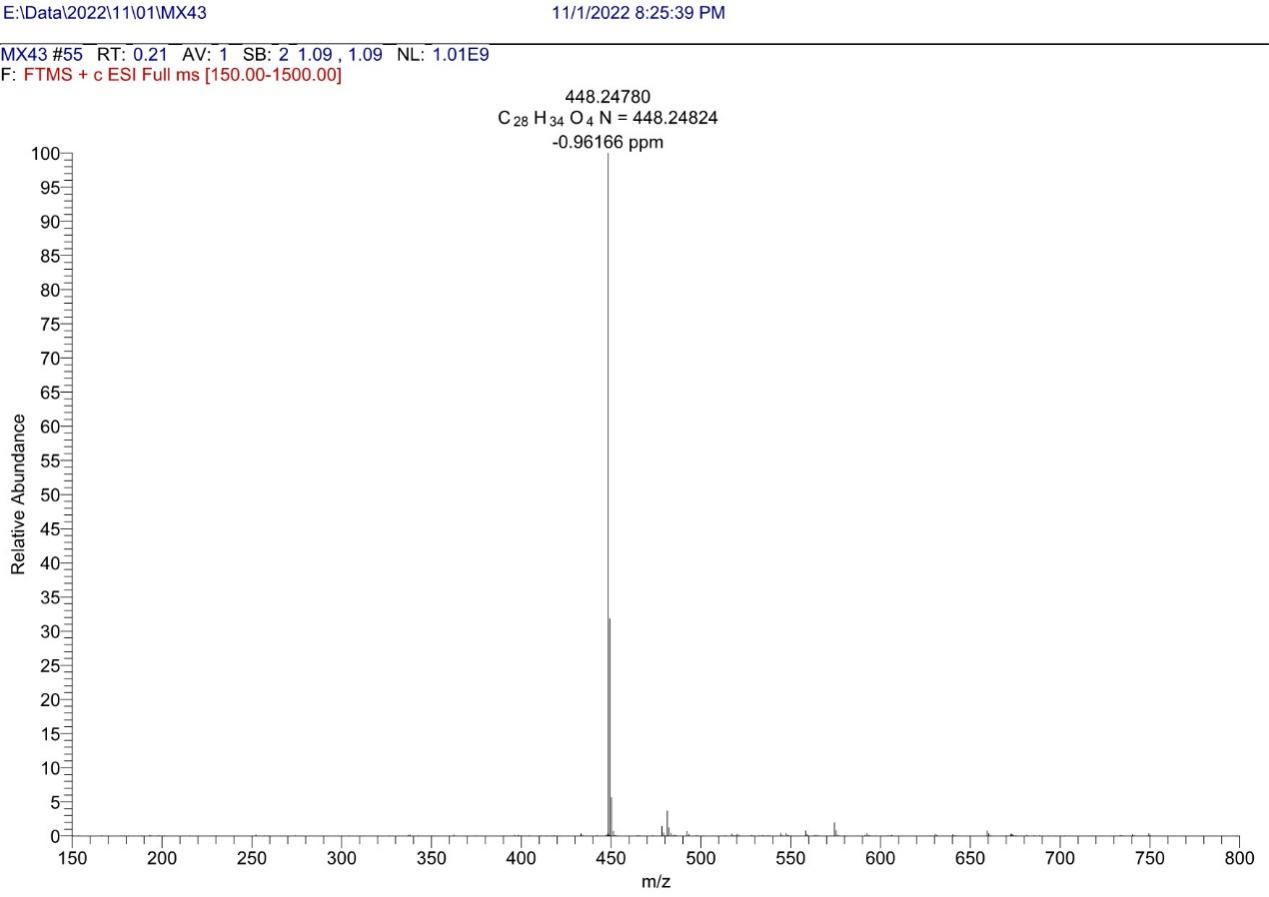


LCMS


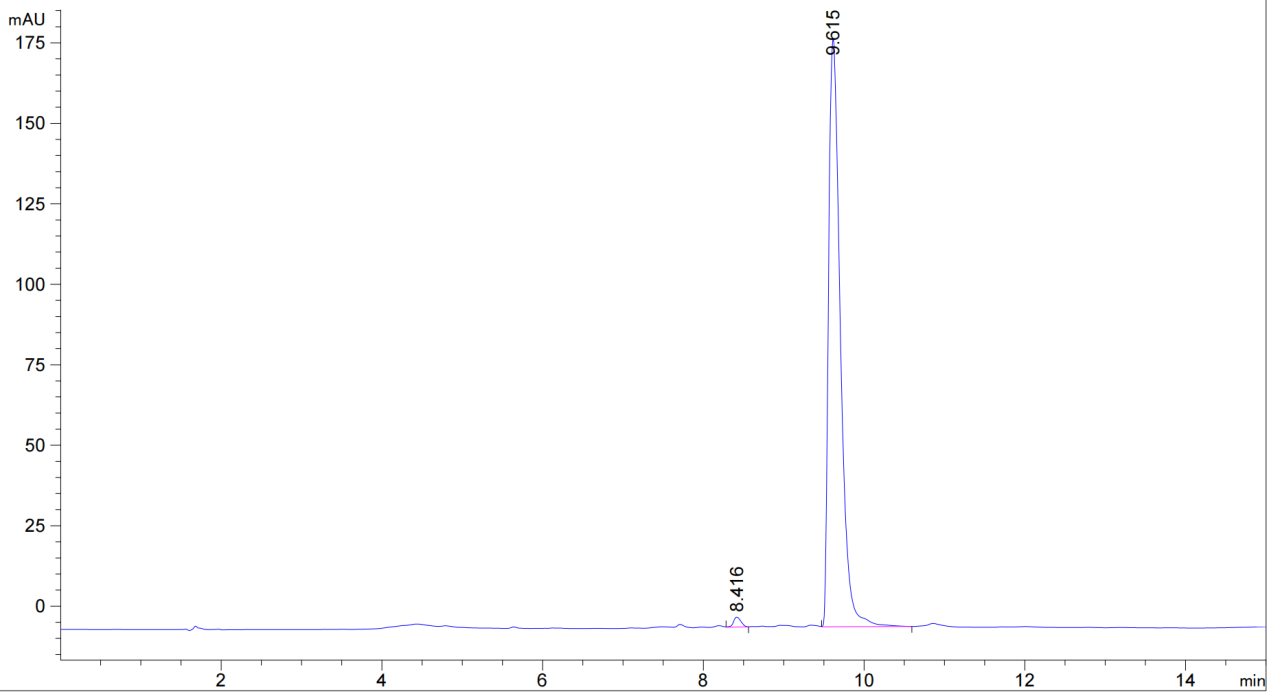


Compound **6**, a turmeric powder with a melting point of 233–235 °C, was synthesized according to the **synthetic procedure**. This reaction provided the desired compound in 92% yield.

**^1^H NMR** (600 MHz, DMSO-*d*_6_): δ 9.91 (s, 1H), 9.06 (s, 1H), 8.23 (d, *J* = 9.1 Hz, 1H), 8.15–7.84 (m, 1H), 7.76 (s, 1H), 7.16 (s, 1H), 4.96 (t, *J* = 6.3 Hz, 2H), 4.93 (d, *J* = 2.4 Hz, 2H), 4.11 (s, 3H), 4.08 (s, 3H), 3.96 (s, 3H), 3.66 (t, *J* = 2.4 Hz, 1H), 3.23 (t, *J* = 6.4 Hz, 2H).

**^13^C NMR** (151 MHz, DMSO-*d*_6_): δ 150.3, 149.1, 149.0, 145.5, 143.7, 137.5, 133.0, 128.2, 126.8, 123.5, 121.4, 120.2, 119.9, 113.0, 109.2, 78.9, 78.8, 61.9, 57.1, 56.3, 56.2, 55.4, 26.0.

**HRMS (ESI)** *m/z* calc for C_23_H_22_NO_4_Br^+^ [M–Br]^+^: 376.15433, found: 376.15399.

^1^H NMR


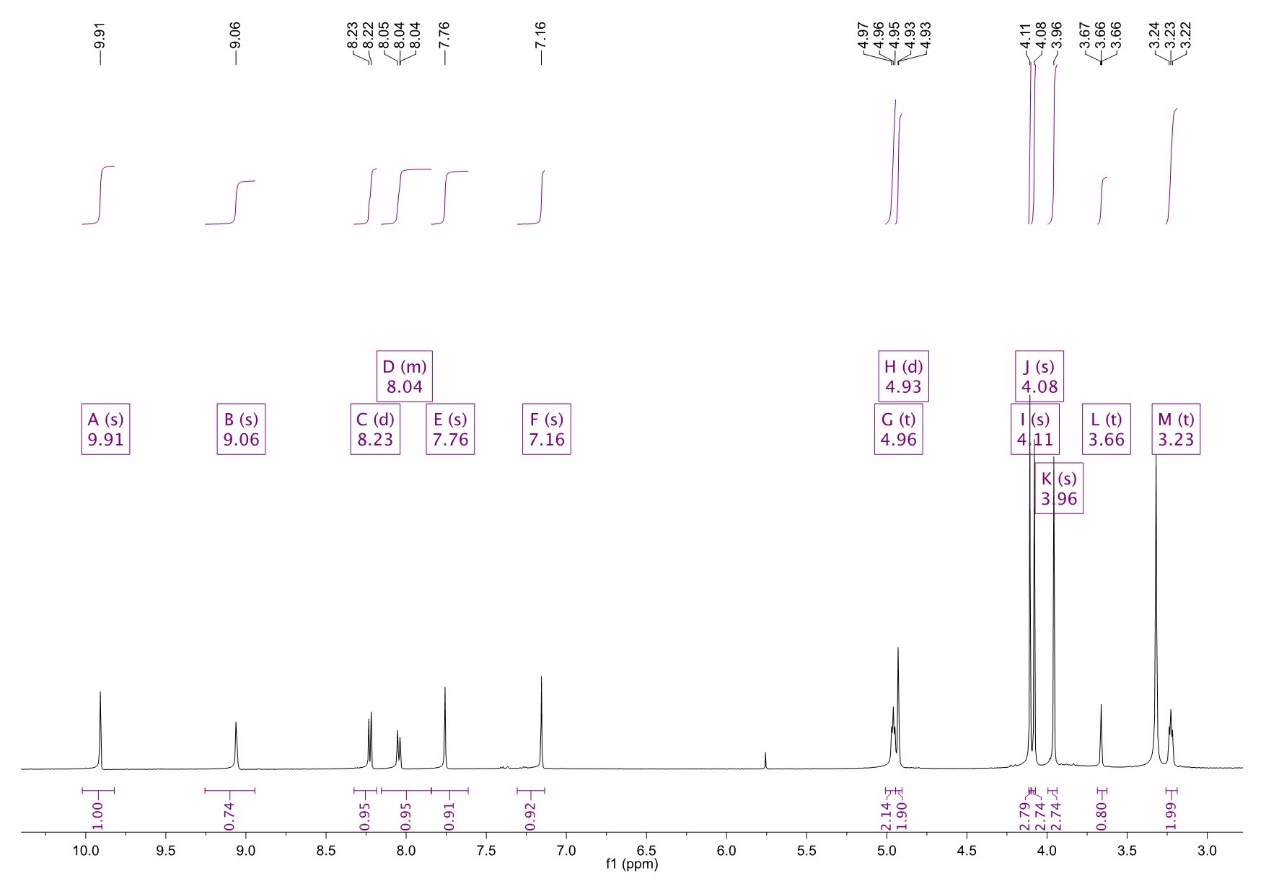


^13^C NMR


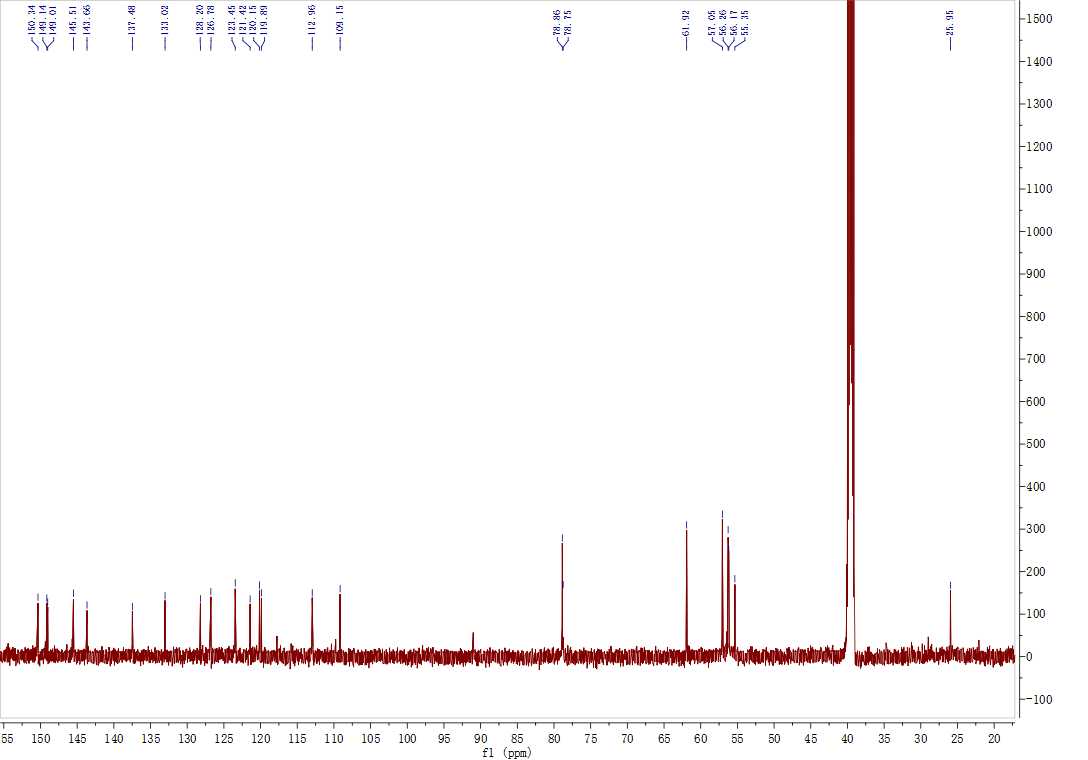


HRMS (ESI)


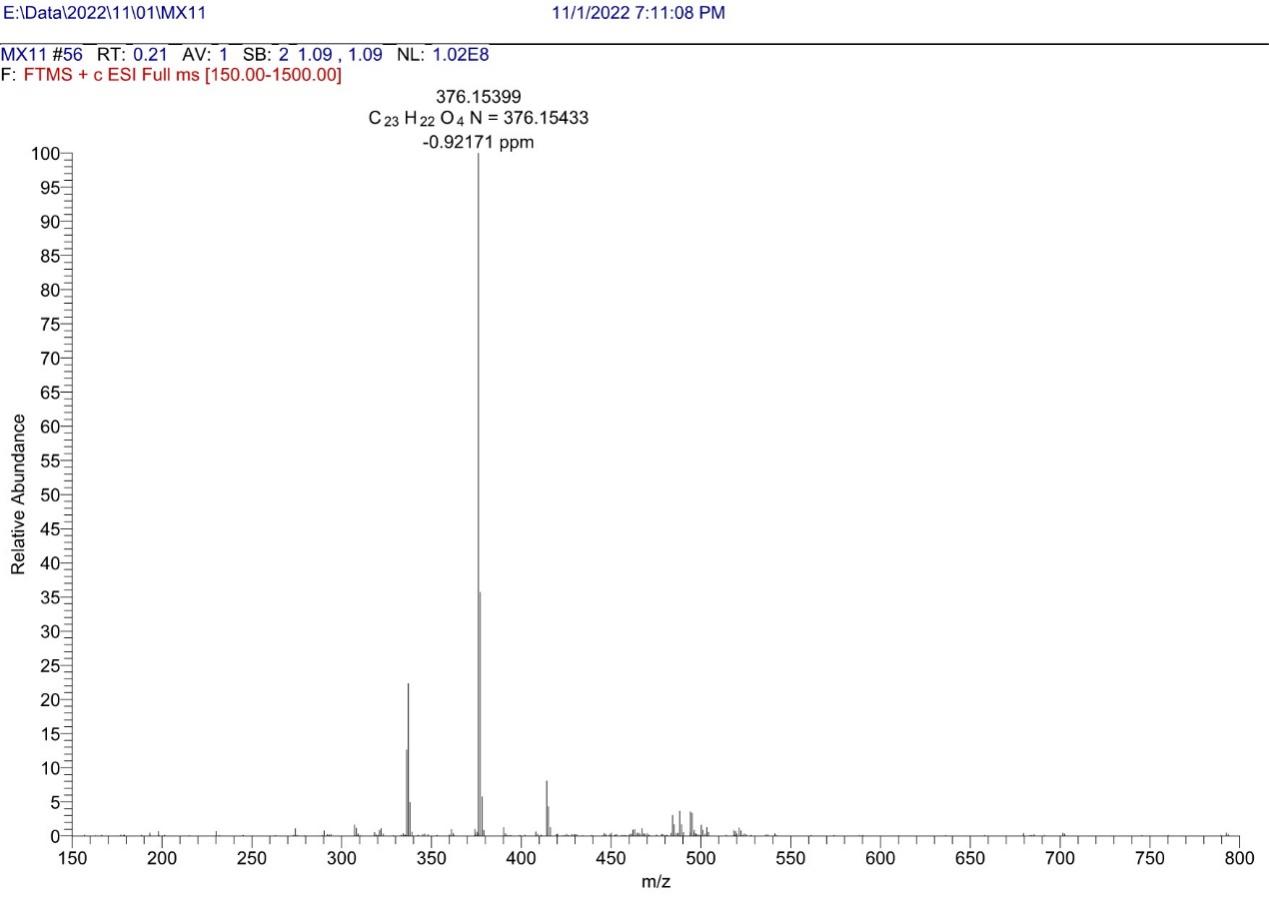

Compound **7**, a turmeric powder with a melting point of 157–159 °C, was synthesized according to the **synthetic procedure**. This reaction provided the desired compound in 82% yield.

**^1^H NMR** (600 MHz, DMSO-*d*_6_): δ 9.89 (s, 1H), 9.04 (s, 1H), 8.22 (d, *J* = 9.1 Hz, 1H), 8.04 (d, *J* = 9.1 Hz, 1H), 7.74 (s, 1H), 7.49 (d, *J* = 7.0 Hz, 2H), 7.46–7.41 (m, 2H), 7.40–7.35 (m, 1H), 7.22 (s, 1H), 5.21 (s, 2H), 4.95 (t, *J* = 6.3 Hz, 2H), 4.10 (s, 3H), 4.08 (s, 3H), 3.95 (s, 3H), 3.21 (t, *J* = 6.4 Hz, 2H).

**^13^C NMR** (151 MHz, DMSO-*d*_6_): δ 150.2, 149.7, 147.4, 145.4, 144.2, 139.0, 137.5, 136.4, 133.0, 131.5, 129.3, 129.0, 128.5, 128.2, 128.1, 128.0, 126.7, 126.3, 121.4, 121.2, 119.1, 112.4, 112.0, 70.0, 62.1, 57.1, 56.9, 54.3, 35.7, 26.7.

**HRMS (ESI)** *m/z* calc for C_34_H_32_NO_4_Br^+^ [M–Br]^+^: 518.23258, found: 518.23188.

**LCMS (254 nm):** *m/z* for C_34_H_32_NO_4_Br^+^ [M–Br]^+^: 518.2, 96.2% pure.

^1^H NMR


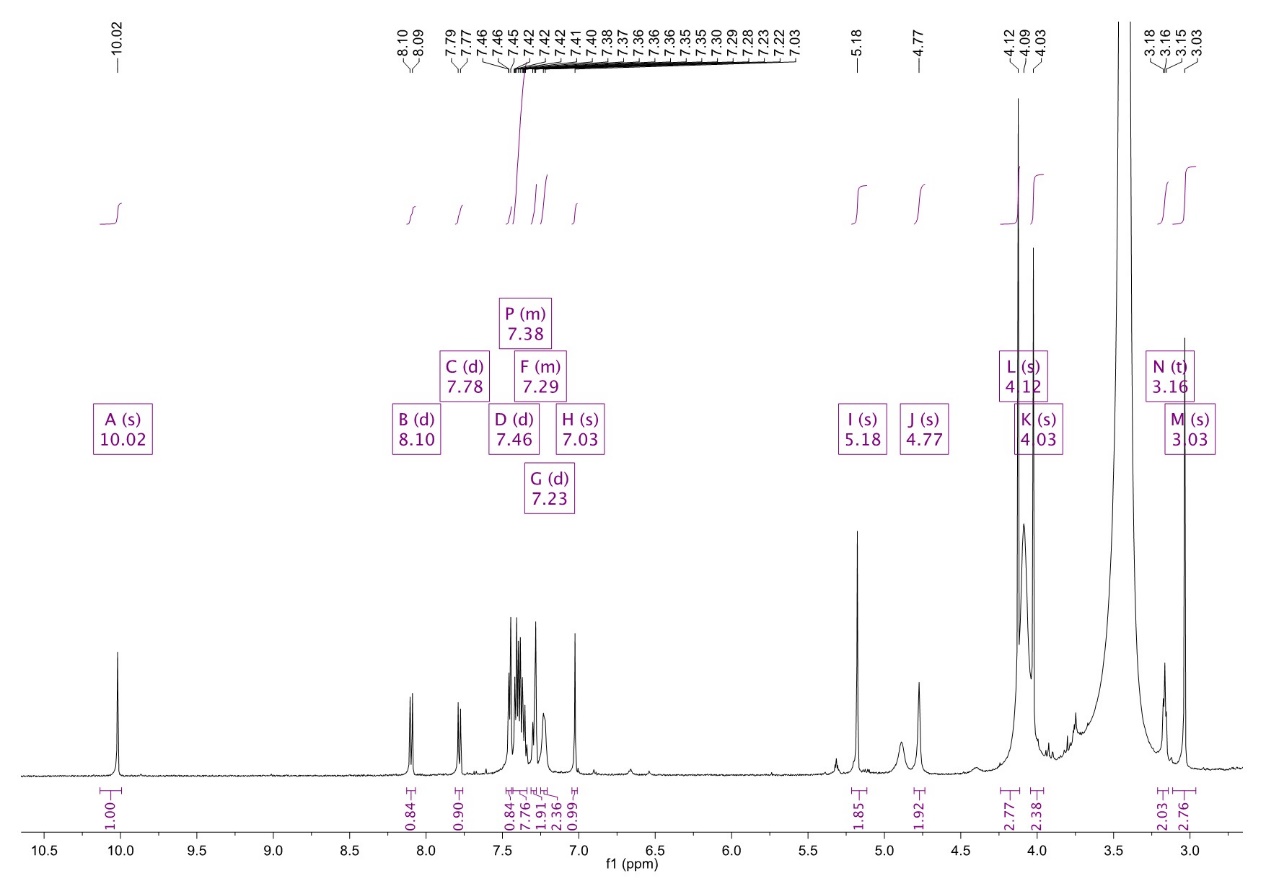


^13^C NMR


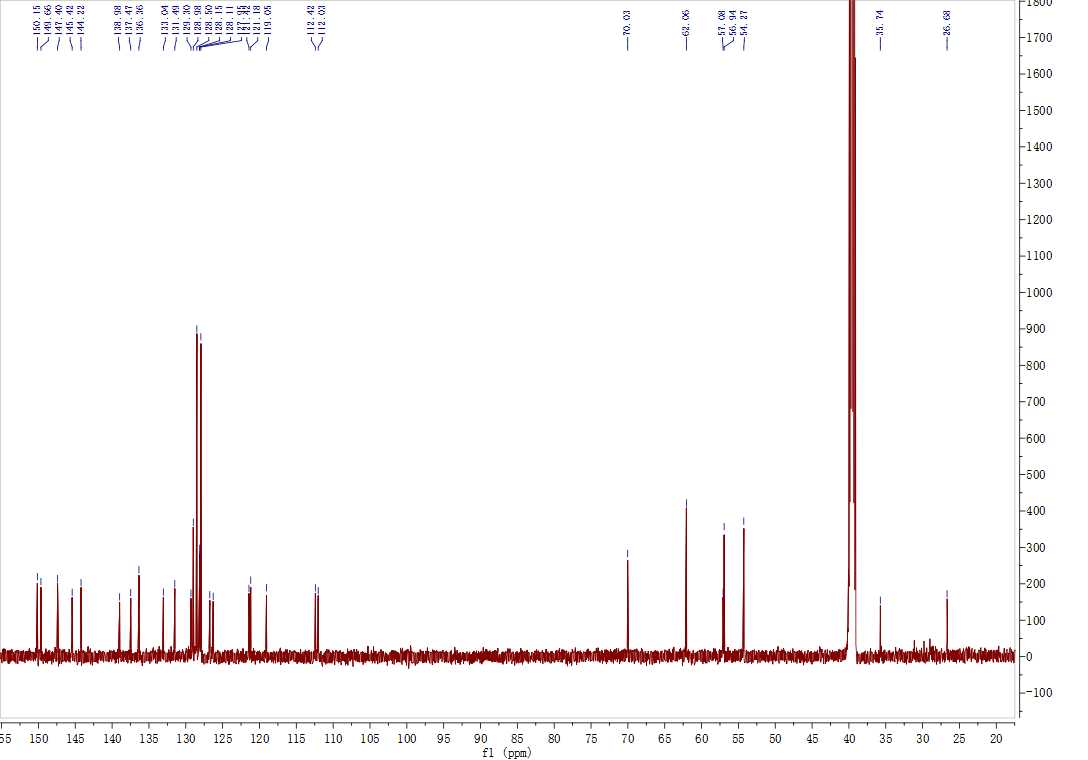


HRMS (ESI)


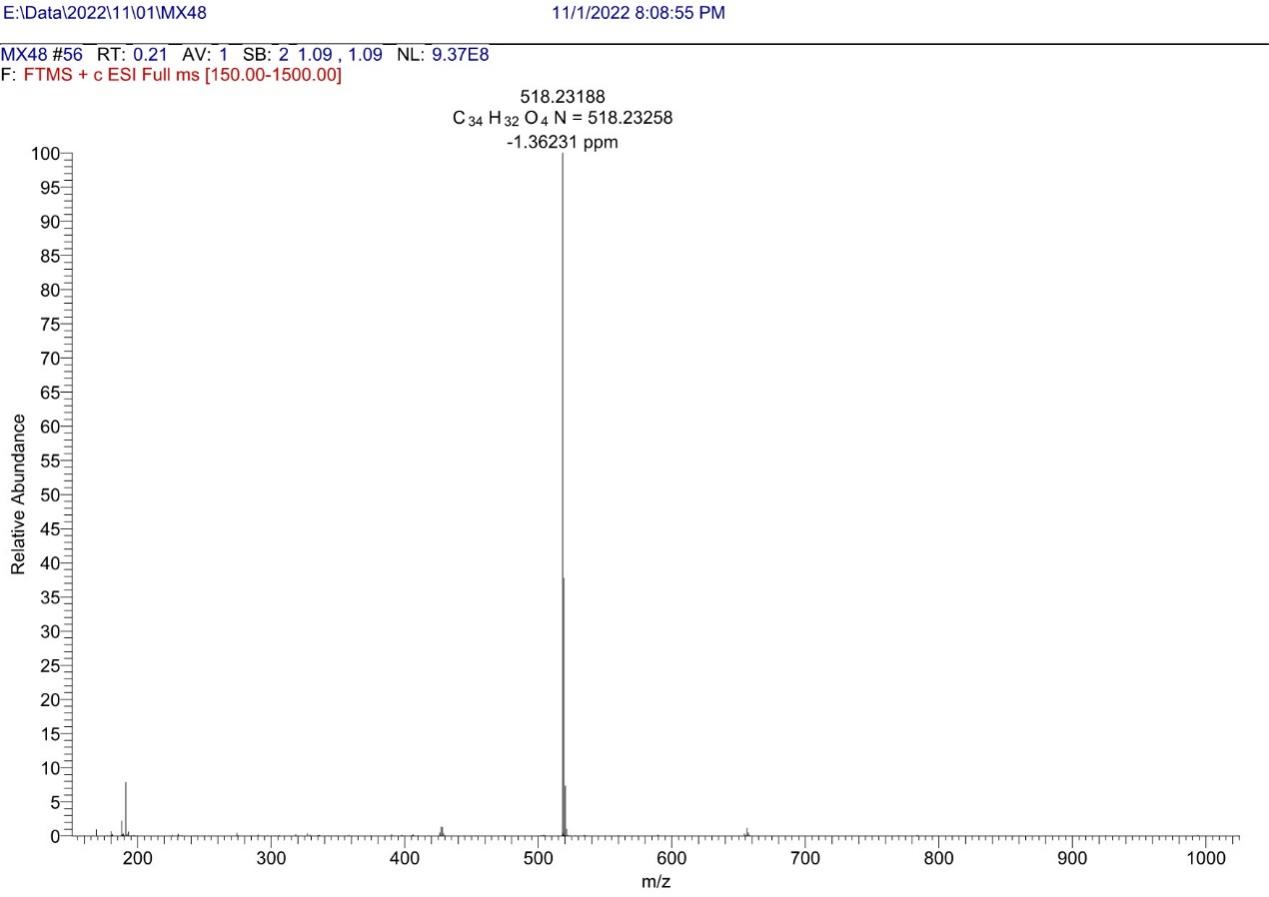


LCMS


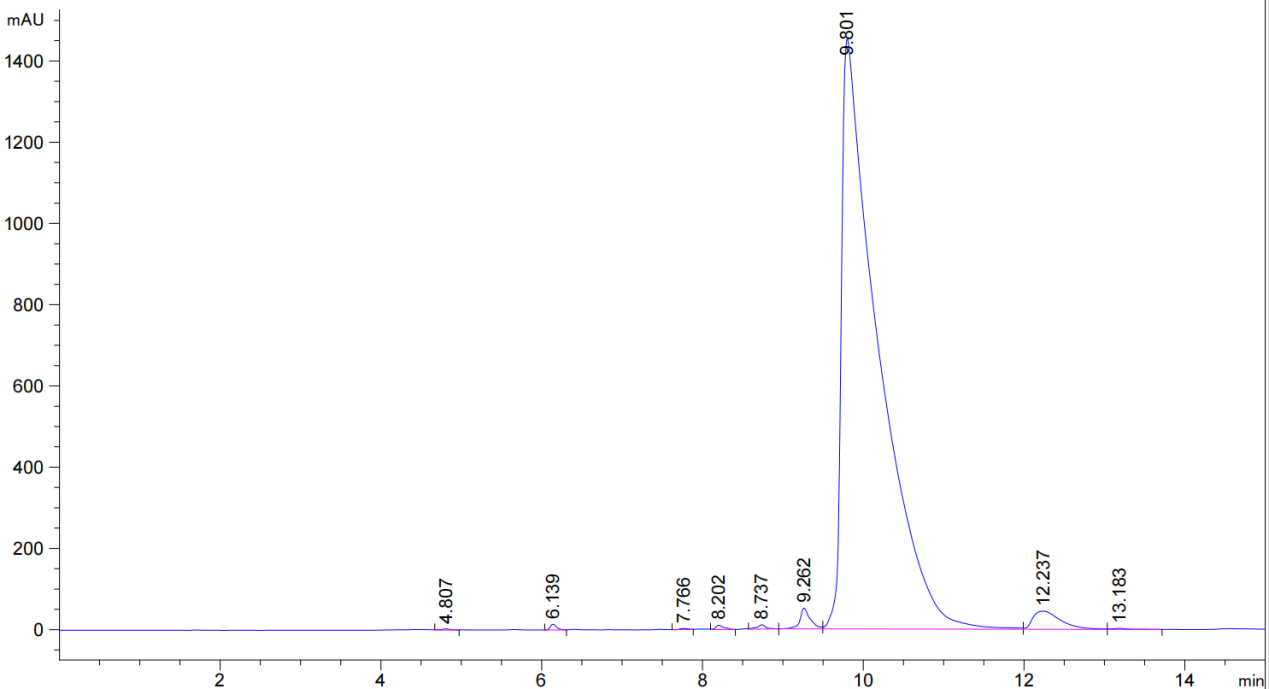


Compound **8**, a turmeric powder with a melting point of 141–143 °C, was synthesized according to the **synthetic procedure**. This reaction provided the desired compound in 88% yield.

**^1^H NMR** (600 MHz, DMSO-*d*_6_): δ 9.94 (s, 1H), 8.19 (d, *J* = 9.5 Hz, 1H), 8.00 (d, *J* = 9.4 Hz, 1H), 7.51 (s, 1H), 7.17 (s, 1H), 6.13 (d, *J* = 14.3 Hz, 1H), 5.91 (dd, *J* = 15.3, 6.5 Hz, 1H), 5.77–5.70 (m, 1H), 5.30 (dd, *J* = 15.7, 6.6 Hz, 1H), 4.85 (s, 2H), 4.60 (d, *J* = 6.1 Hz, 2H), 4.09 (dd, *J* = 19.0, 6.9 Hz, 6H), 3.98 (s, 2H), 3.76 (s, 3H), 3.12 (t, *J* = 5.7 Hz, 2H), 1.72 (dd, *J* = 21.2, 5.6 Hz, 6H).

**^13^C NMR** (151 MHz, MeOD): δ 151.7, 149.8, 146.2, 145.9, 138.9, 135.2, 132.7, 132.5, 131.8, 130.9, 130.1, 127.4, 127.0, 122.9, 122.8, 120.5, 114.1, 113.5, 101.4, 70.7, 62.6, 59.0, 57.6, 56.9, 34.6, 28.5, 18.2, 18.0.

**HRMS (ESI)** *m/z* calc for C_28_H_32_NO_4_Br^+^ [M–Br]^+^ : 446.23258, found: 446.23240.

**LCMS (254 nm):** *m/z* for C_28_H_32_NO_4_Br^+^ [M–Br]^+^: 446.2, ≥ 99% pure.

^1^H NMR


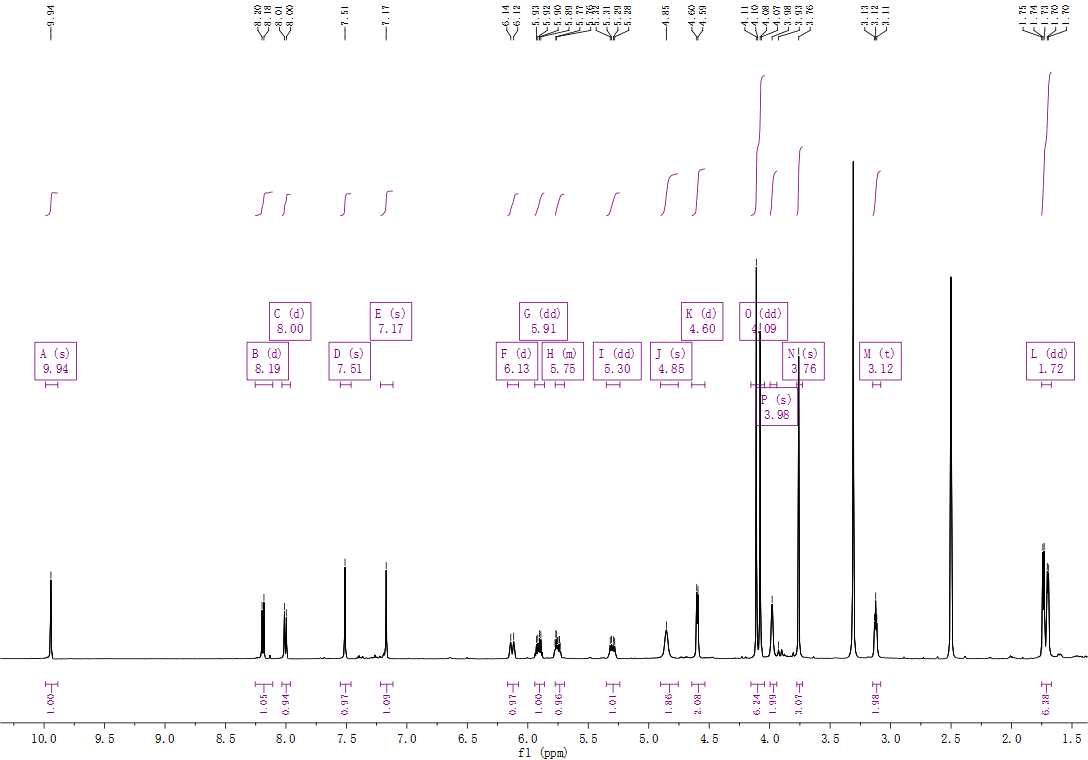


^13^C NMR


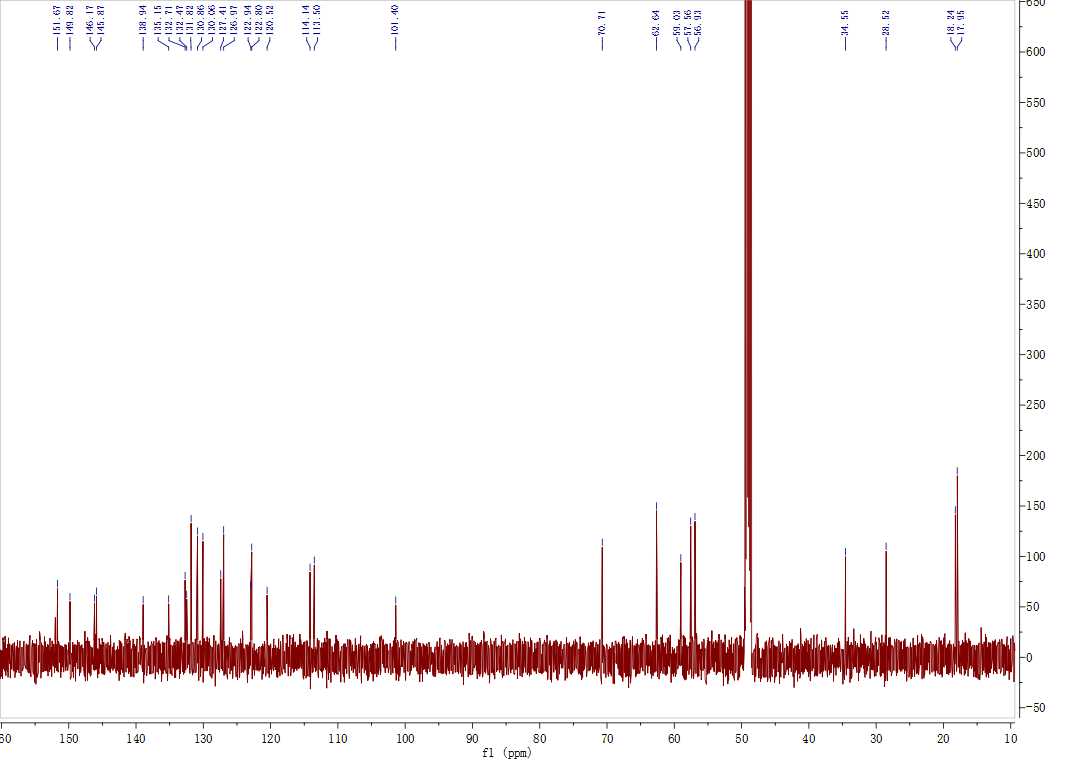


DEPT


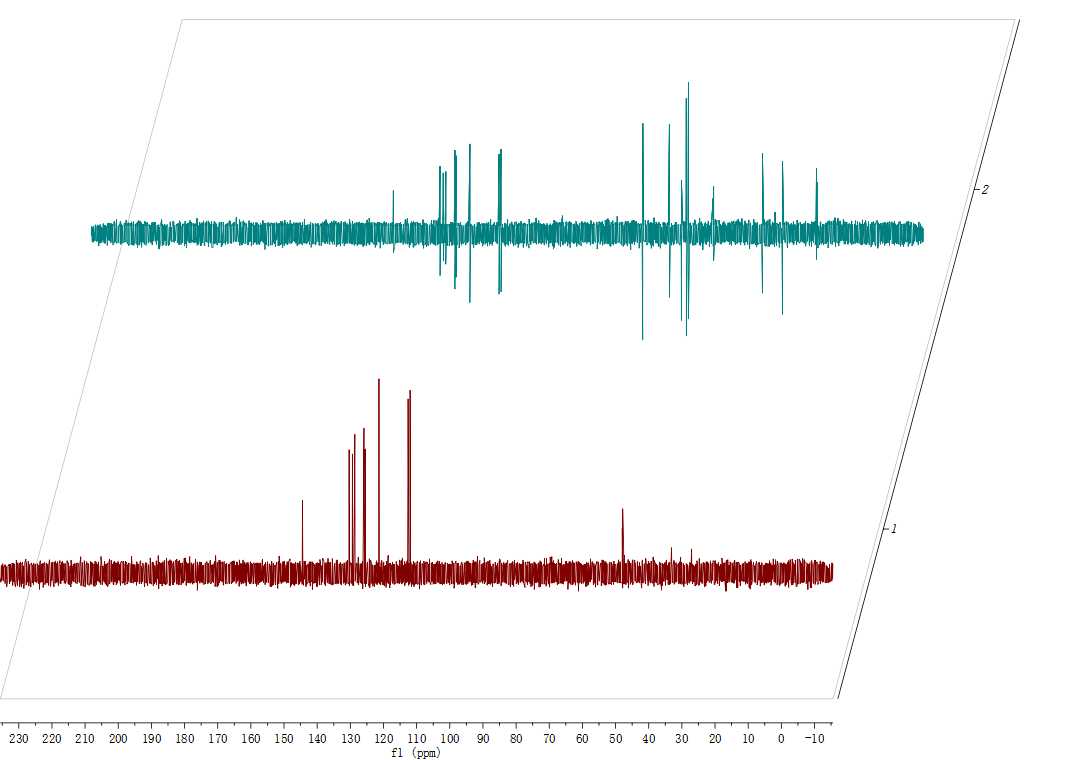


COSY
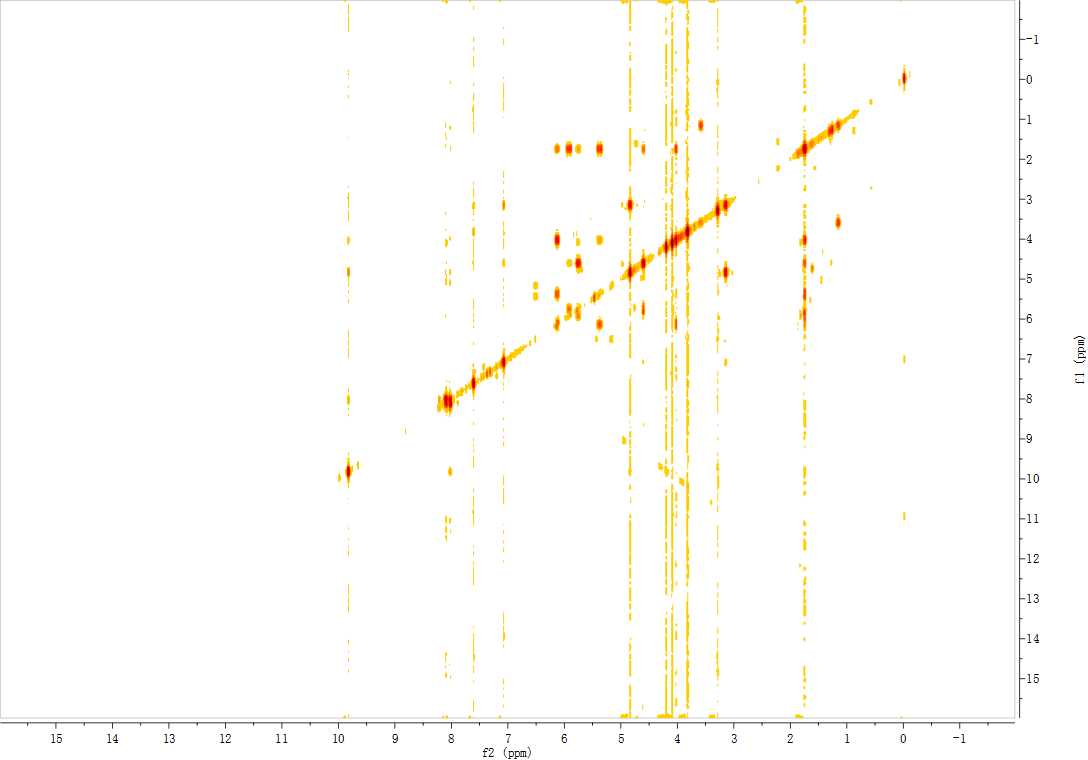


HSQC


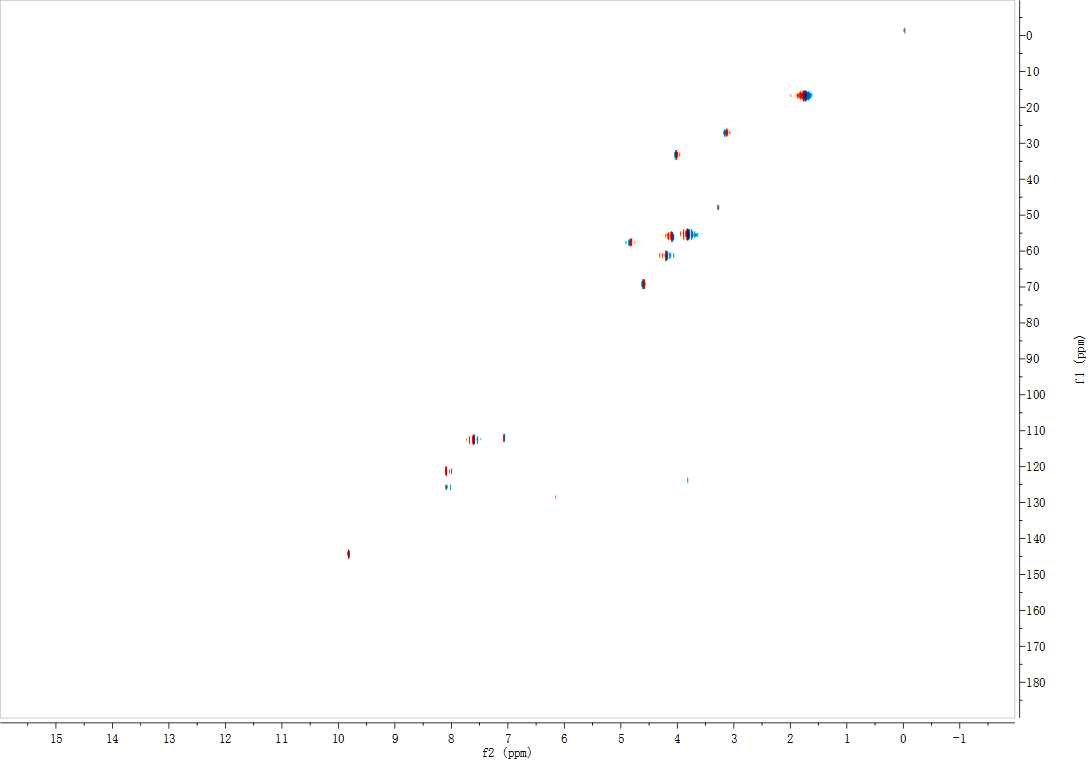


HRMS (ESI)


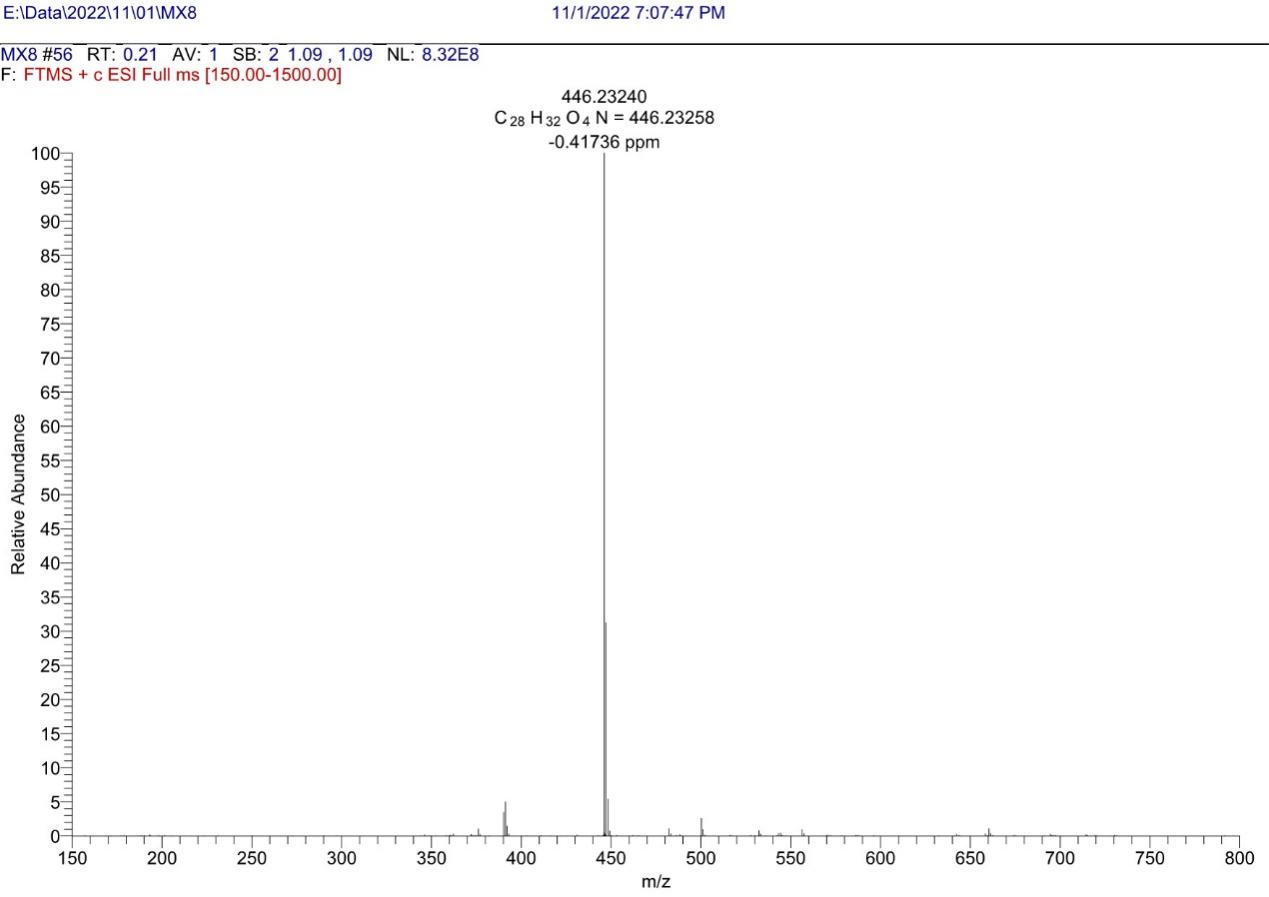


LCMS


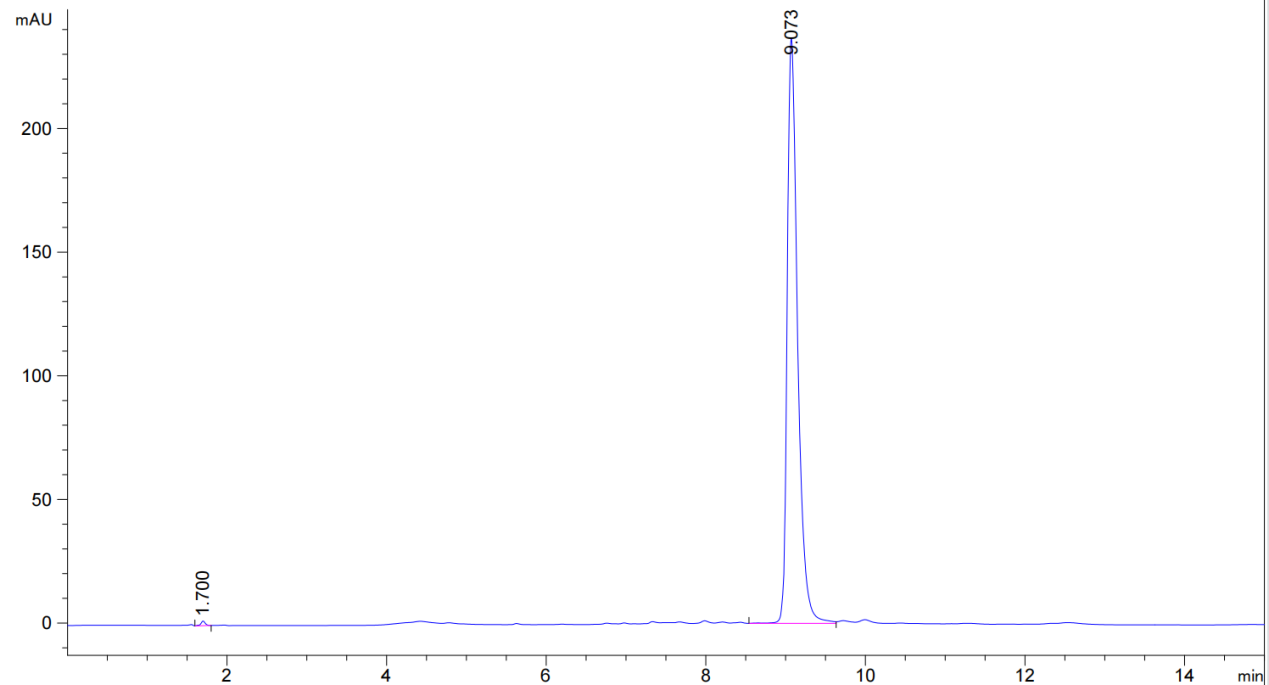

Compound **9**, a turmeric powder with a melting point of 188–190 °C, was synthesized according to the **synthetic procedure**. This reaction provided the desired compound in 74% yield.

**^1^H NMR** (600 MHz, DMSO-*d*_6_): δ 9.89 (s, 1H), 9.05 (s, 1H), 8.22 (d, *J* = 9.2 Hz, 1H), 8.06 (d, *J* = 9.1 Hz, 1H), 7.74 (s, 1H), 6.89 (s, 1H), 5.26 (s, 2H), 4.98 (s, 4H), 4.98 (s, 4H), 4.94 (s, 1H), 4.94 (s, 1H), 4.10 (s, 3H), 4.09 (d, *J* = 15.7 Hz, 6H), 4.08 (s, 3H), 3.96 (s, 3H), 3.18 (t, *J* = 6.5 Hz, 2H), 1.20 (s, 9H).

**^13^C NMR** (151 MHz, DMSO-*d*_6_): δ 208.2, 150.3, 150.1, 148.8, 145.5, 143.6, 137.6, 133.1, 128.3, 126.8, 123.4, 121.4, 119.9, 119.3, 112.3, 109.3, 64.4, 61.9, 57.1, 56.3, 52.8, 44.0, 25.8, 25.4.

**HRMS (ESI)** *m/z* calc for C_26_H_30_NO_5_Br^+^ [M–Br]^+^: 436.21185, found: 436.21088.

^1^H NMR


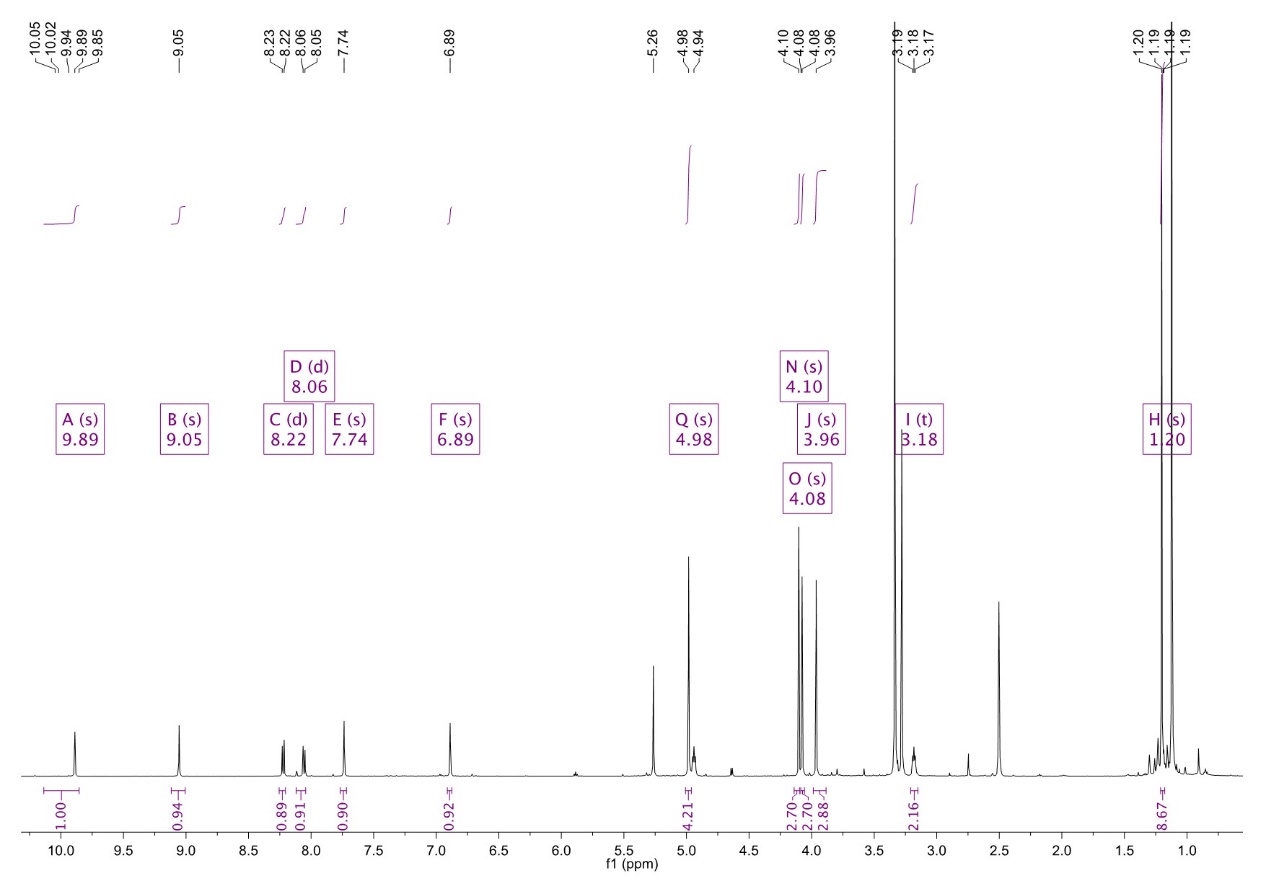


^13^C NMR


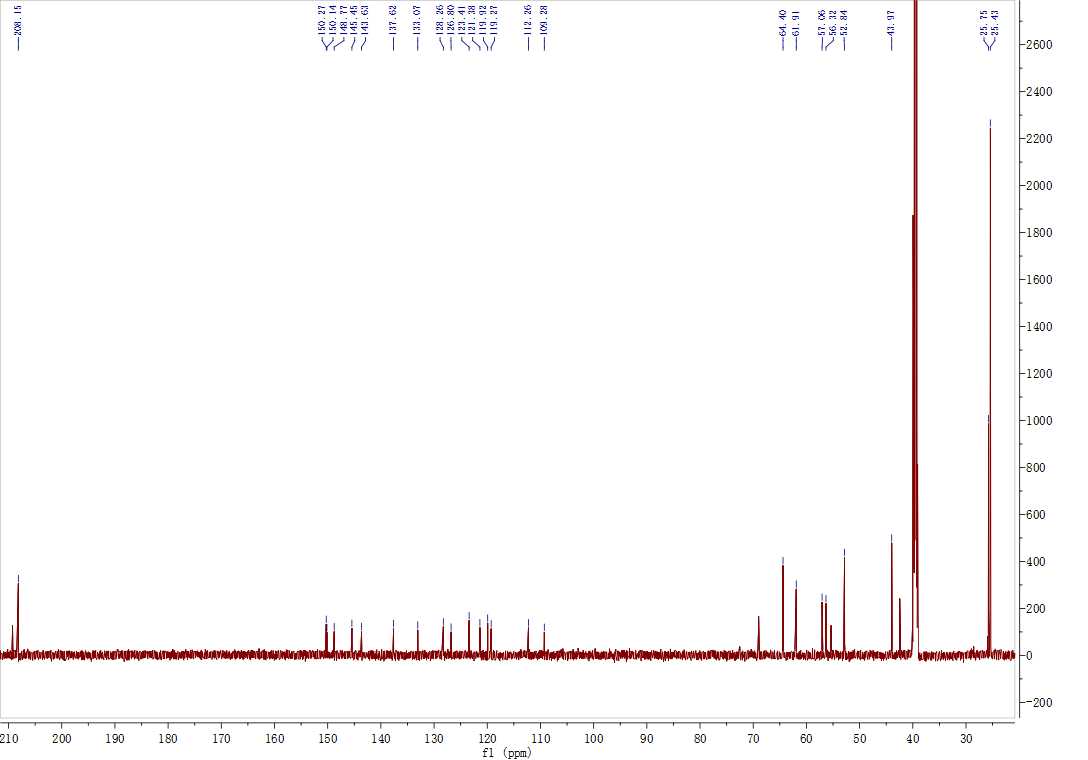


HRMS (ESI)


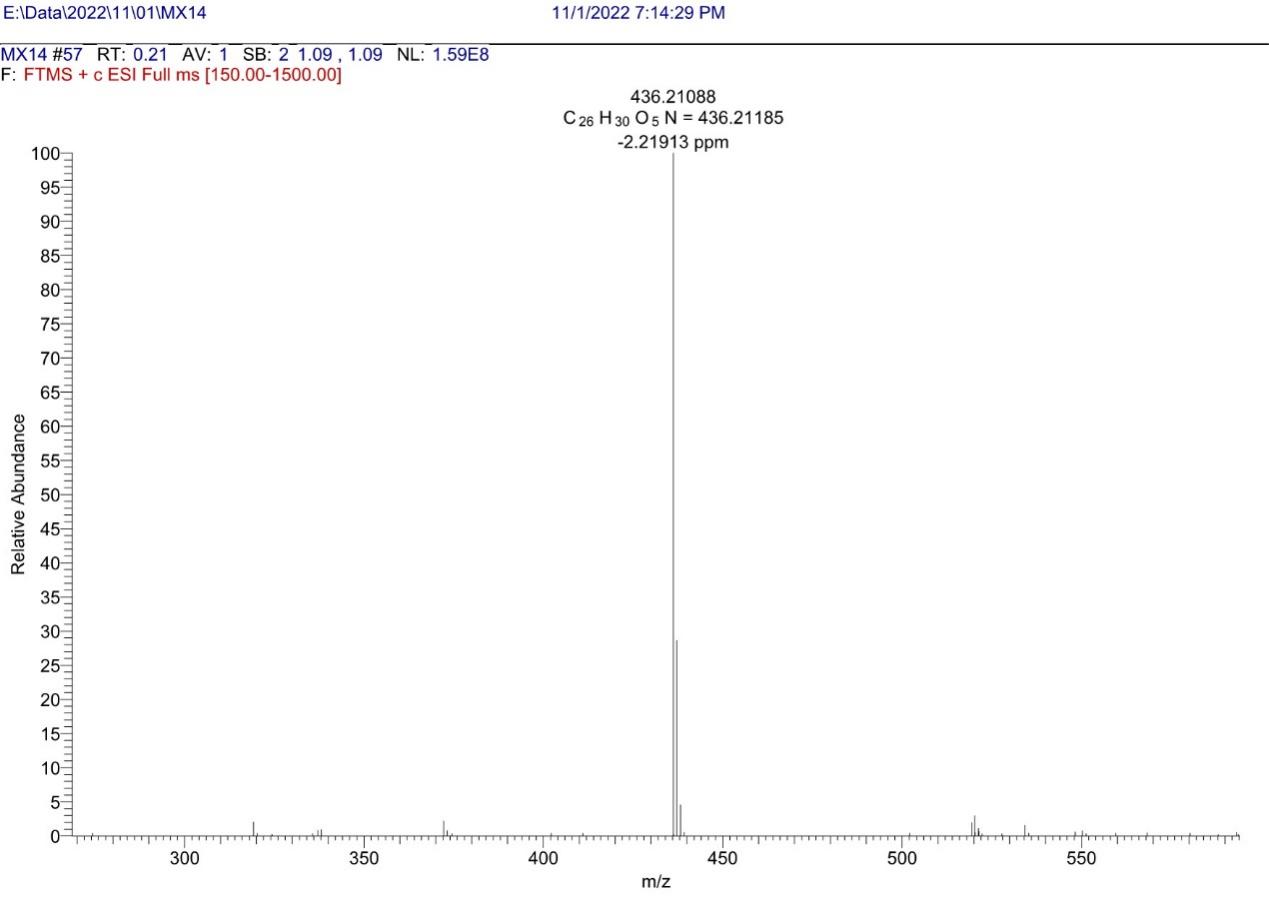

Compound **10**, a turmeric powder with a melting point of 236–238 °C, was synthesized according to the **synthetic procedure**. This reaction provided the desired compound in 78% yield.

**^1^H NMR** (600 MHz, DMSO-*d*_6_): δ 9.90 (s, 1H), 9.04 (d, *J* = 2.7 Hz, 1H), 8.22 (d, *J* = 9.1 Hz, 1H), 8.04 (d, *J* = 9.0 Hz, 1H), 7.75 (s, 1H), 7.03 (s, 1H), 4.94 (t, *J* = 6.4 Hz, 2H), 4.91 (s, 2H), 4.20 (q, *J* = 7.1 Hz, 2H), 4.10 (s, 3H), 4.08 (s, 3H), 3.97 (s, 3H), 3.19 (t, *J* = 6.4 Hz, 2H), 1.24 (t, *J* = 7.1 Hz, 3H).

**^13^C NMR** (151 MHz, MeOD): δ 170.3, 152.0, 151.9, 151.2, 146.5, 145.8, 139.5, 135.2, 129.7, 128.0, 124.6, 123.4, 121.7, 121.6, 114.4, 110.8, 66.9, 62.6, 62.5, 57.7, 57.4, 57.2, 27.7, 14.5.

**HRMS (ESI)** *m/z* calc for C_24_H_26_NO_6_Br^+^ [M–Br]^+^: 424.17546, found: 424.17556.

^1^H NMR


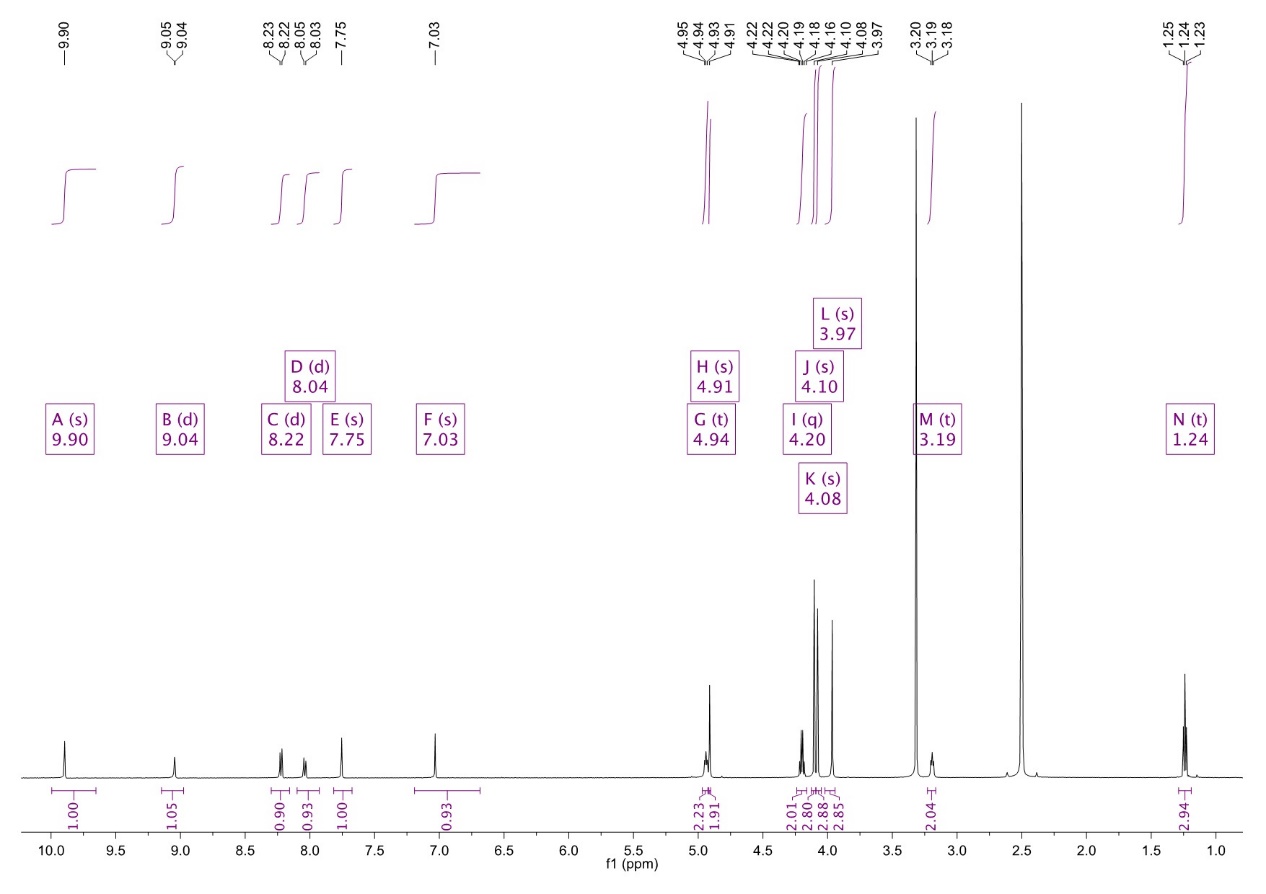


^13^C NMR


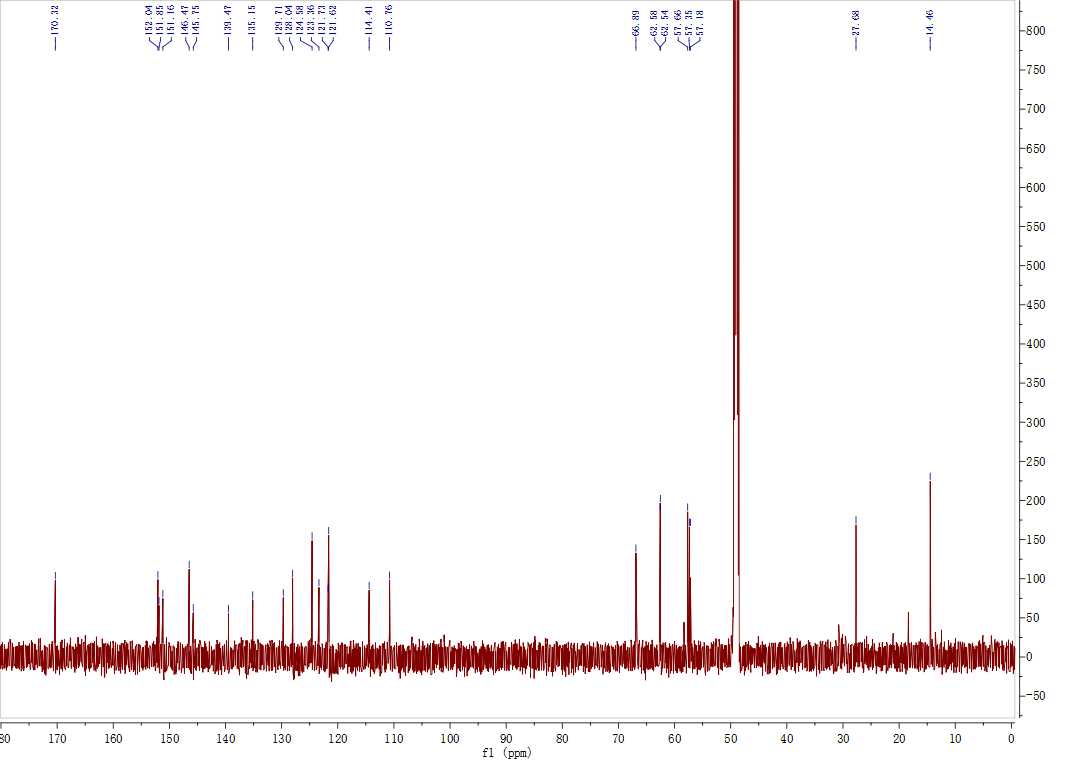


HRMS (ESI)


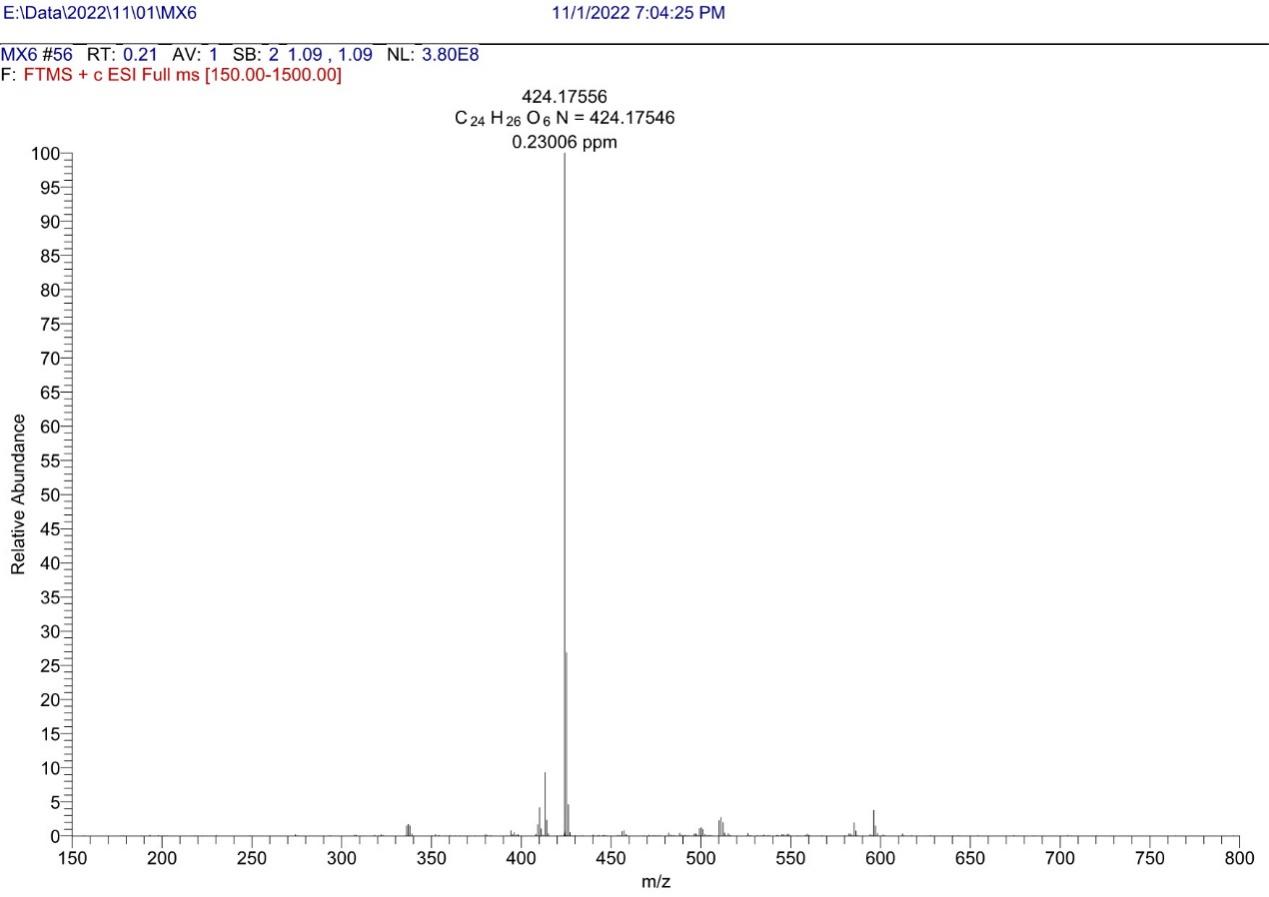

Compound **11**, a turmeric powder with a melting point of 214–216 °C, was synthesized according to the **synthetic procedure**. This reaction provided the desired compound in 80% yield.

**^1^H NMR** (600 MHz, DMSO-*d*_6_): δ 9.96 (s, 1H), 9.19 (s, 1H), 8.26 (d, *J* = 9.1 Hz, 1H), 8.10 (d, *J* = 9.0 Hz, 1H), 7.91 (s, 1H), 7.27 (s, 1H), 4.99 (t, *J* = 6.3 Hz, 2H), 4.12 (s, 3H), 4.09 (s, 3H), 3.96 (s, 3H), 3.24 (t, J = 6.3 Hz, 2H), 2.60 (t, *J* = 7.2 Hz, 2H), 1.69 (q, *J* = 7.3 Hz, 2H), 1.00 (t, *J* = 7.4 Hz, 3H).

**^13^C NMR** (151 MHz, DMSO-*d*_6_): δ 171.4, 151.4, 151.3, 146.4, 144.3, 142.1, 137.3, 133.2, 128.5, 127.3, 125.9, 124.2, 123.4, 122.2, 121.8, 110.5, 62.4, 57.6, 57.1, 55.9, 35.5, 25.9, 18.5, 13.8.

**HRMS (ESI)** *m/z* calc for C_24_H_26_NO_5_Cl^+^ [M–Cl]^+^: 408.18055, found: 408.17998.

^1^H NMR


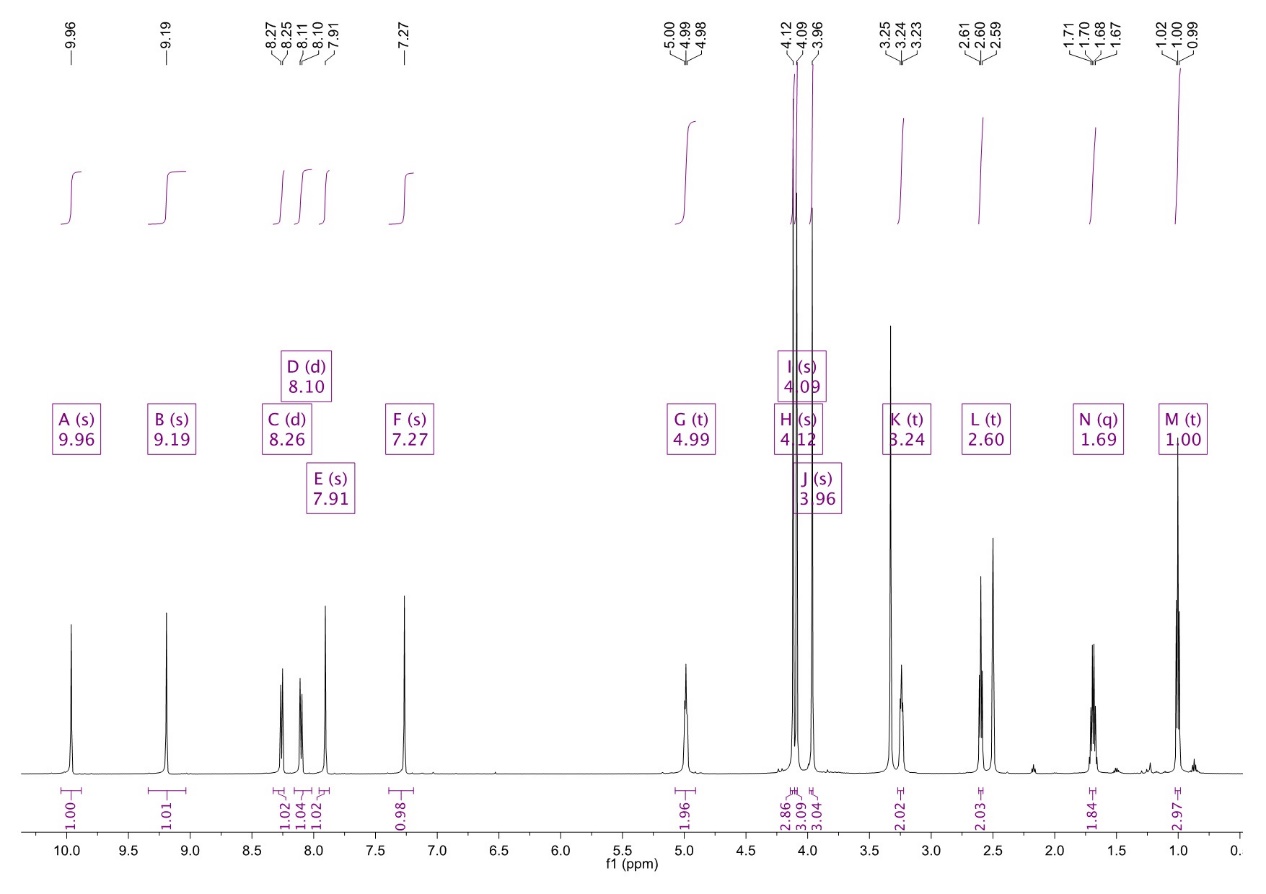


^13^C NMR


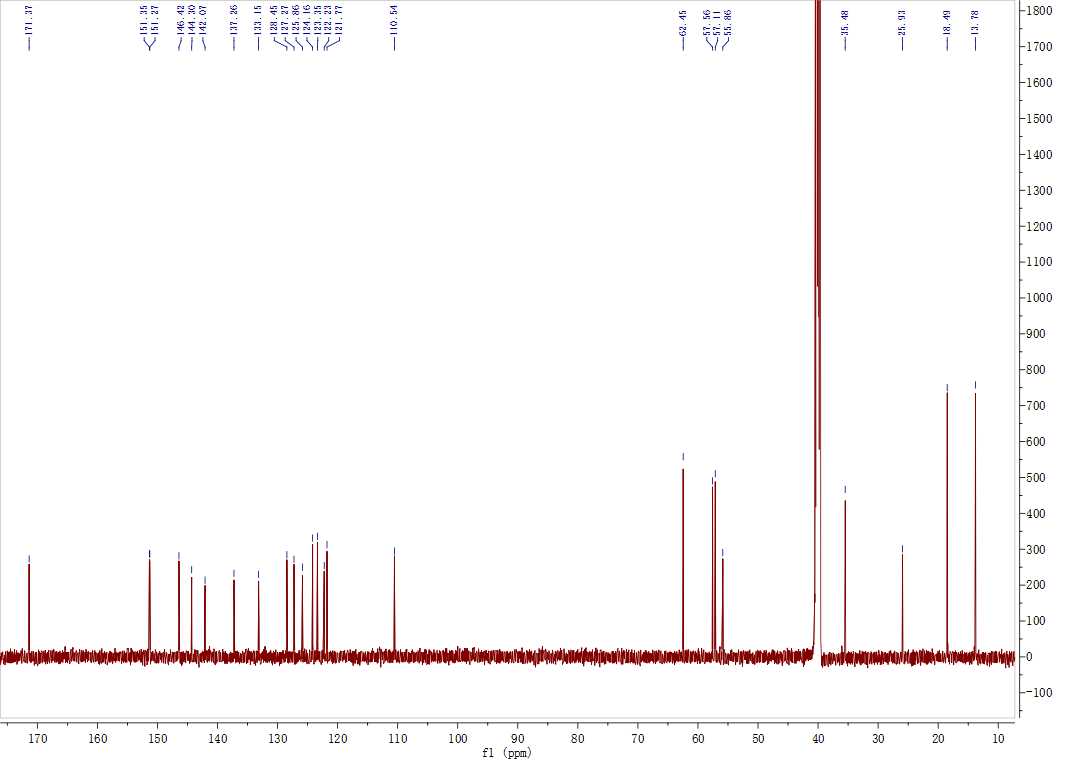


HRMS (ESI)


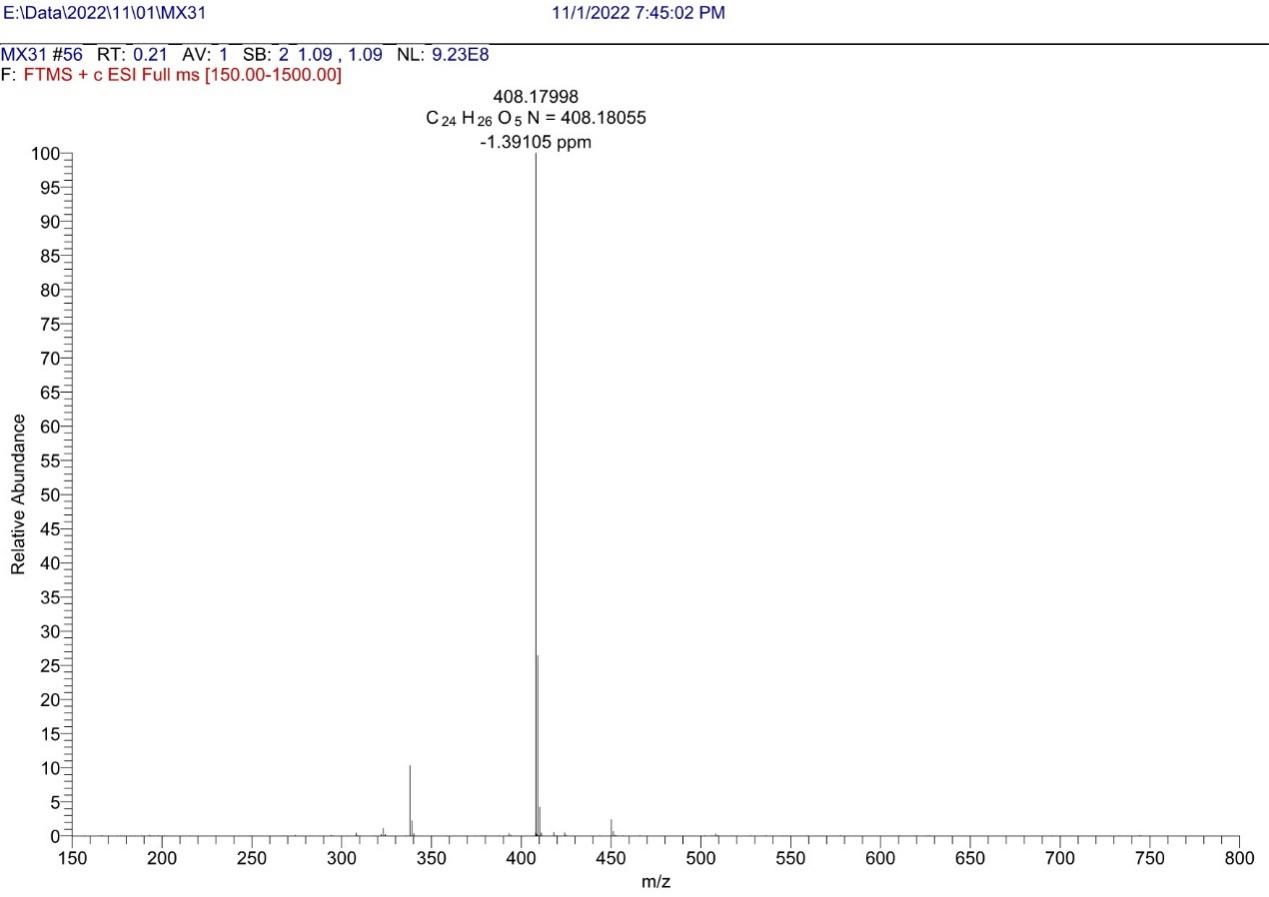

Compound **12**, a turmeric powder with a melting point of 202–204 °C, was synthesized according to the **synthetic procedure**. This reaction provided the desired compound in 85% yield.

**^1^H NMR** (600 MHz, DMSO-*d*_6_): δ 9.96 (s, 1H), 9.16 (s, 1H), 8.26 (d, *J* = 9.1 Hz, 1H), 8.08 (d, *J* = 9.0 Hz, 1H), 7.90 (s, 1H), 7.26 (s, 1H), 4.98 (t, *J* = 6.4 Hz, 2H), 4.12 (s, 3H), 4.09 (s, 3H), 3.95 (s, 3H), 3.24 (t, *J* = 6.4 Hz, 2H), 1.33 (s, 9H).

**^13^C NMR** (151 MHz, DMSO-*d*_6_): δ 175.6, 150.9, 150.8, 146.0, 143.8, 142.0, 136.8, 132.7, 128.0, 126.8, 125.3, 123.7, 122.8, 121.7, 121.2, 110.1, 62.0, 57.1, 56.7, 55.4, 38.6, 26.8, 25.4.

**HRMS (ESI)** *m/z* calc for C_25_H_28_NO_5_Cl^+^ [M–Cl]^+^ : 422.19620, found: 422.19565.

^1^H NMR


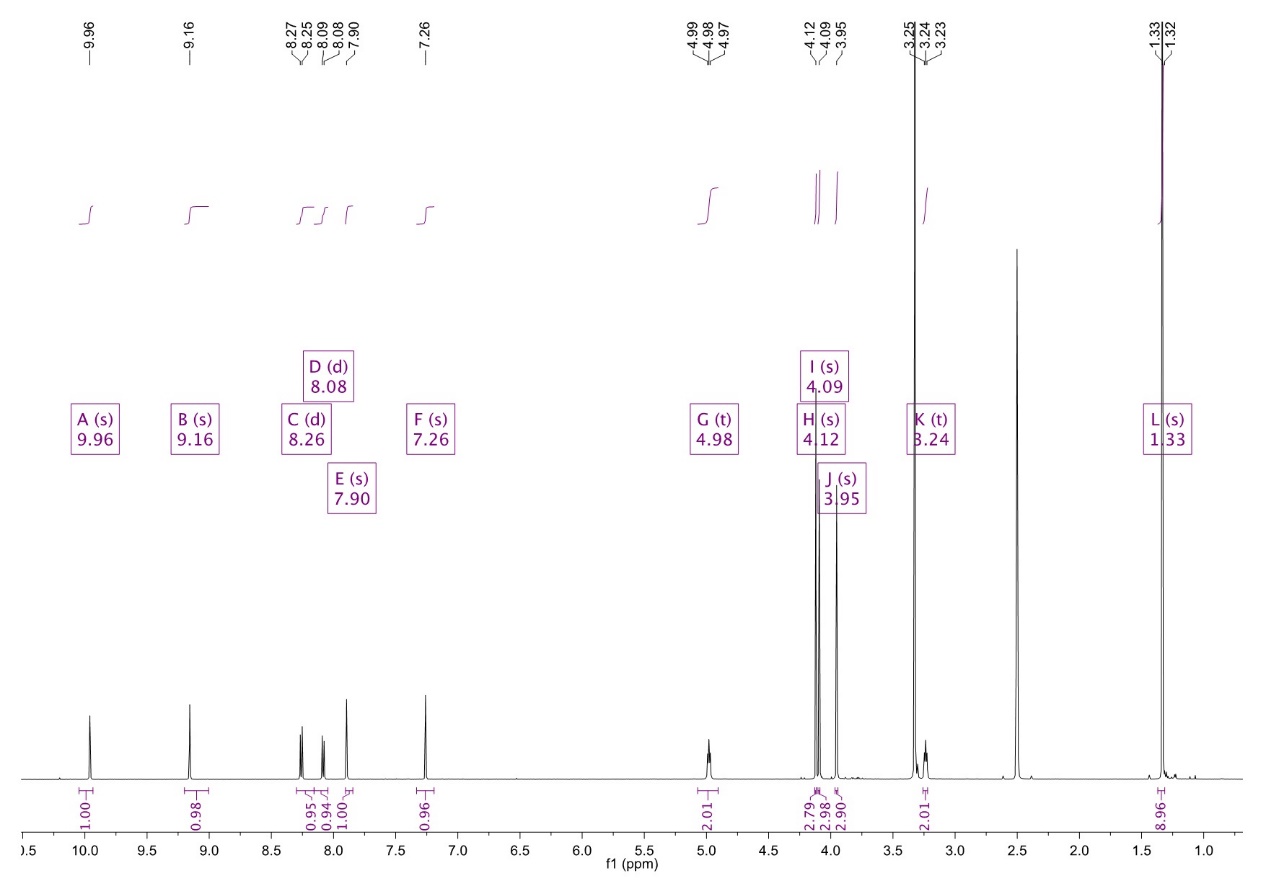


^13^C NMR


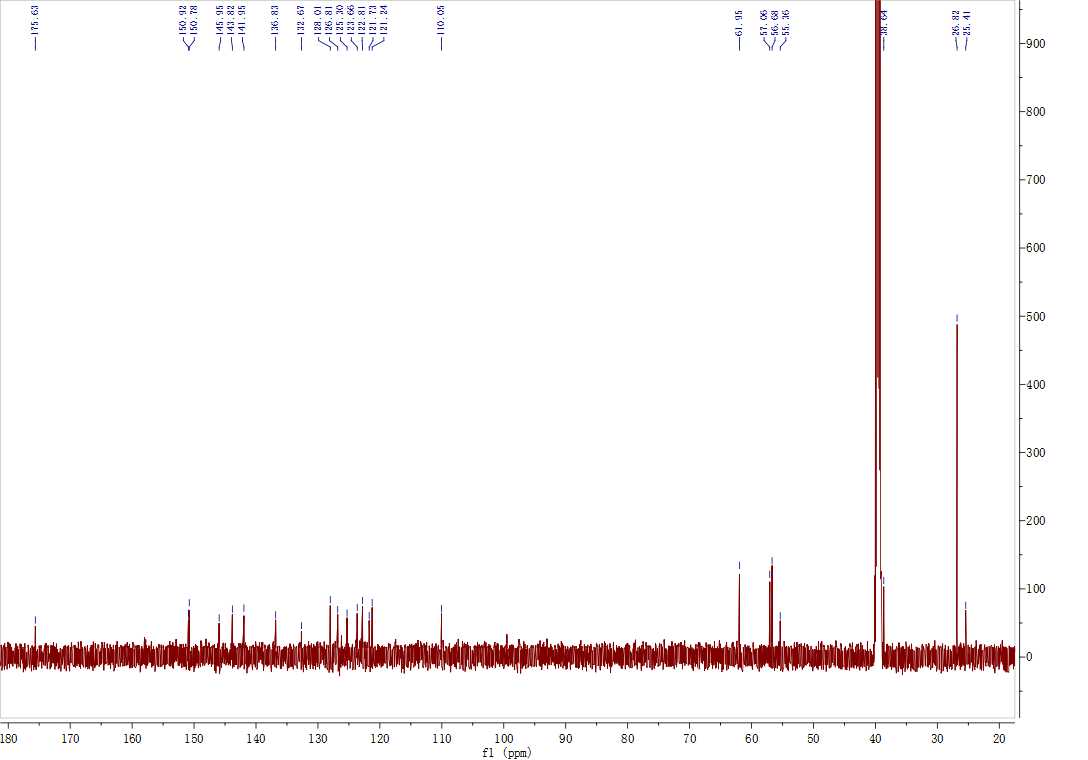


HRMS (ESI)


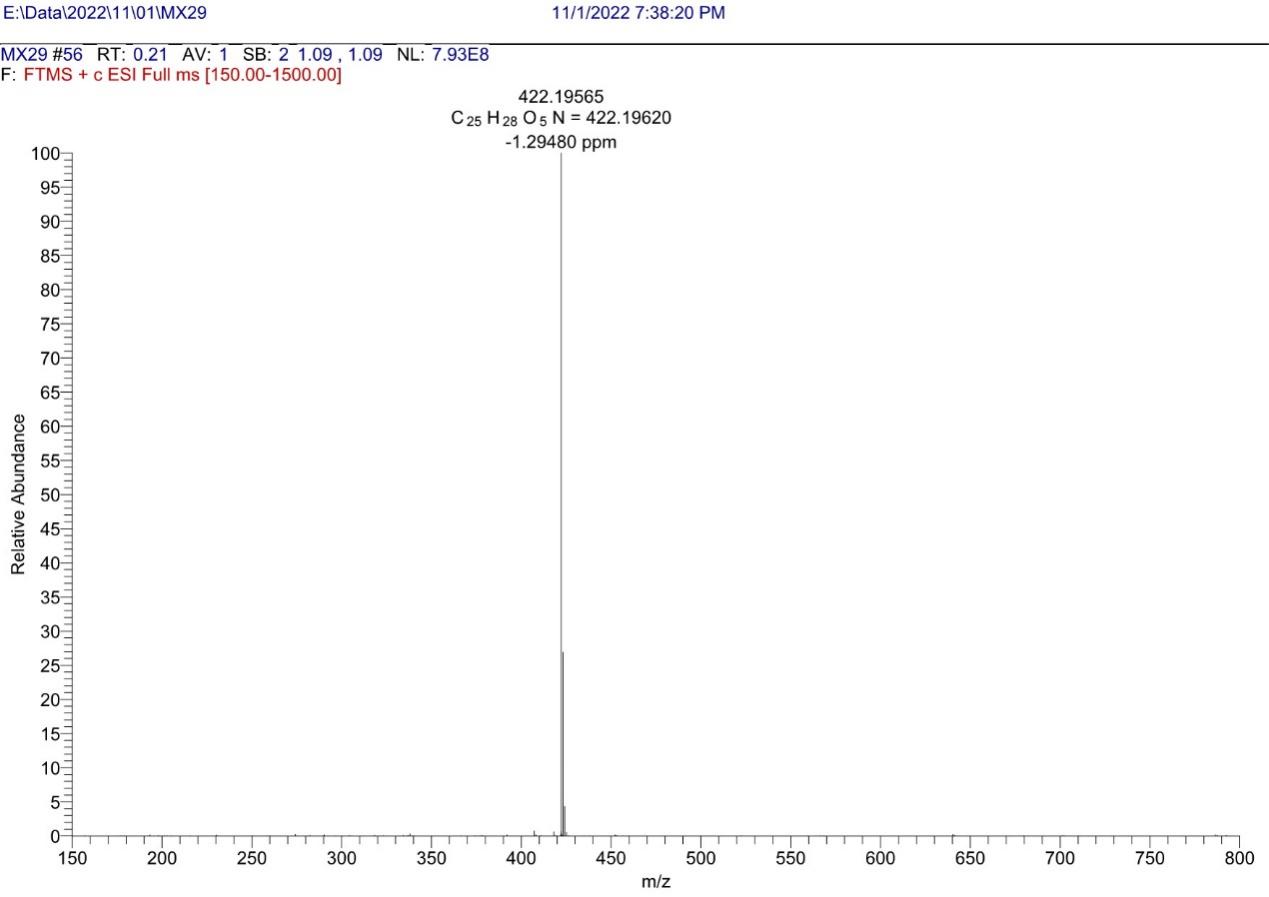

Compound **13**, a turmeric powder with a melting point of 191–193 °C, was synthesized according to the **synthetic procedure**. This reaction provided the desired compound in 77% yield.

**^1^H NMR** (600 MHz, MeOD): δ 9.86 (s, 1H), 9.00 (s, 1H), 8.17 (d, *J* = 9.0 Hz, 1H), 8.12 (d, *J* = 9.0 Hz, 1H), 7.89 (s, 1H), 7.40 (s, 1H), 4.99 (t, *J* = 6.3 Hz, 2H), 4.23 (s, 3H), 4.13 (s, 3H), 4.08 (s, 3H), 3.49–3.45 (m, 2H), 2.83–2.76 (m, 1H), 1.96 (t, *J* = 7.7 Hz, 2H), 1.56 (p, *J* = 7.4 Hz, 2H), 1.02 (t, *J* = 7.4 Hz, 3H), 0.94 (t, *J* = 7.4 Hz, 2H), 0.94 (t, *J* = 7.4 Hz, 2H).

**^13^C NMR** (151 MHz, MeOD): δ 153.3, 152.6, 147.1, 145.9, 141.7, 138.3, 134.7, 129.2, 128.0, 127.9, 125.2, 125.0, 123.7, 123.2, 111.6, 66.5, 62.7, 57.7, 57.3, 52.5, 27.3, 26.8, 22.4, 13.9.

**HRMS (ESI)** *m/z* calc for C_24_H_28_NO_6_SCl^+^ [M–Cl]^+^: 458.16318, found: 458.16310.

^1^H NMR


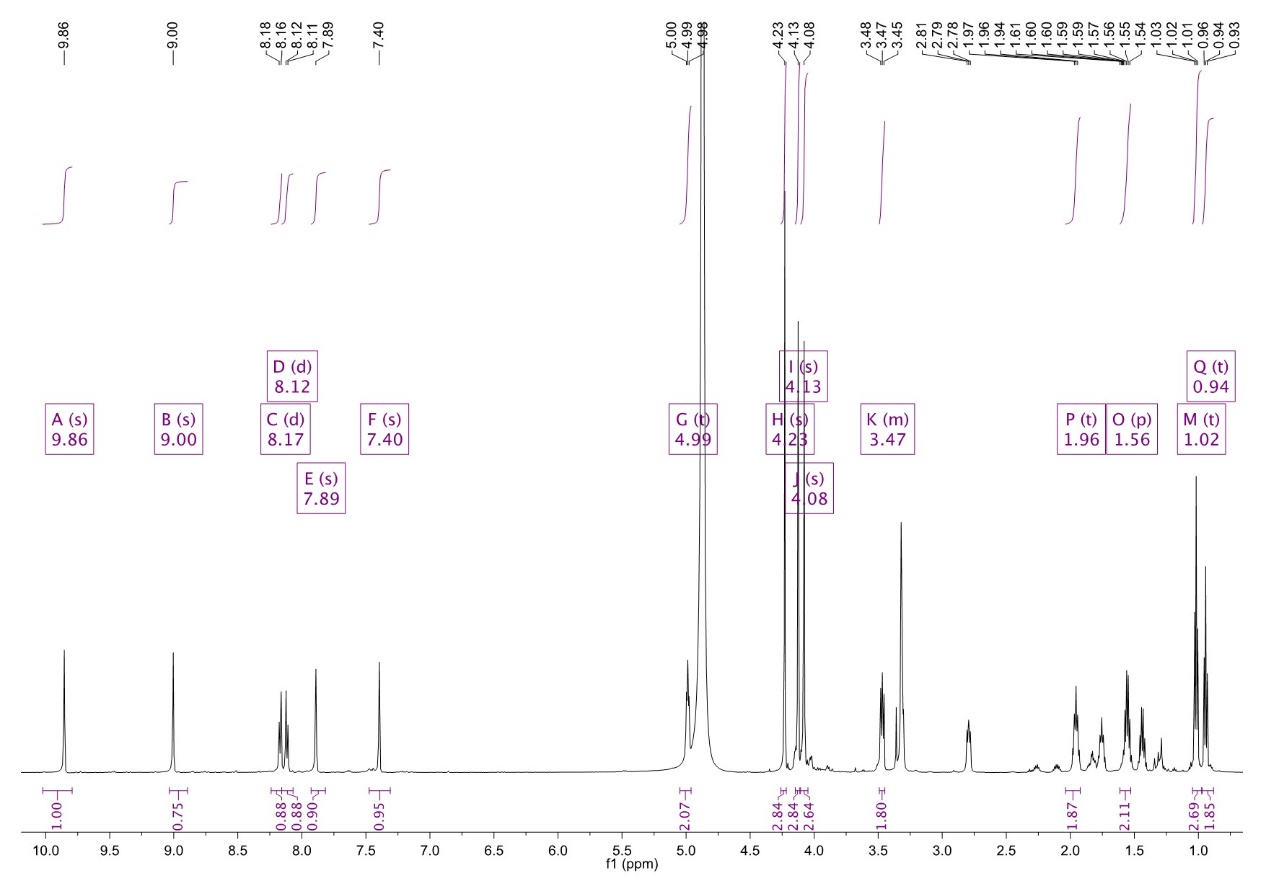


^13^C NMR


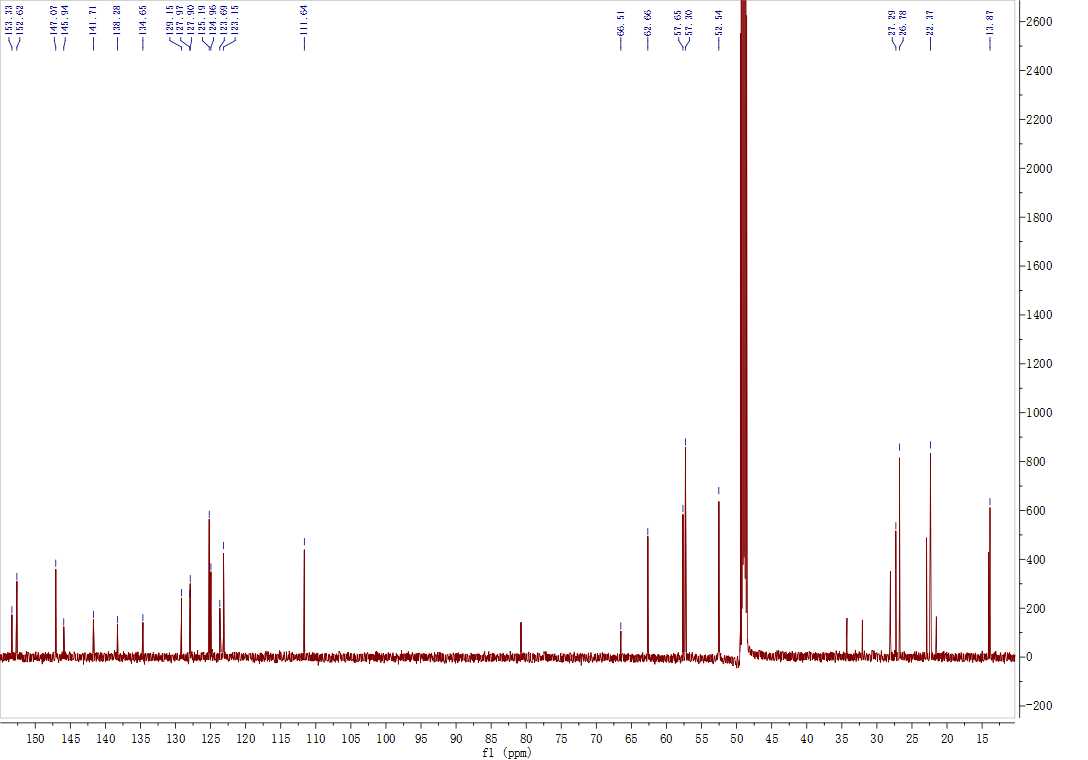


HRMS (ESI)


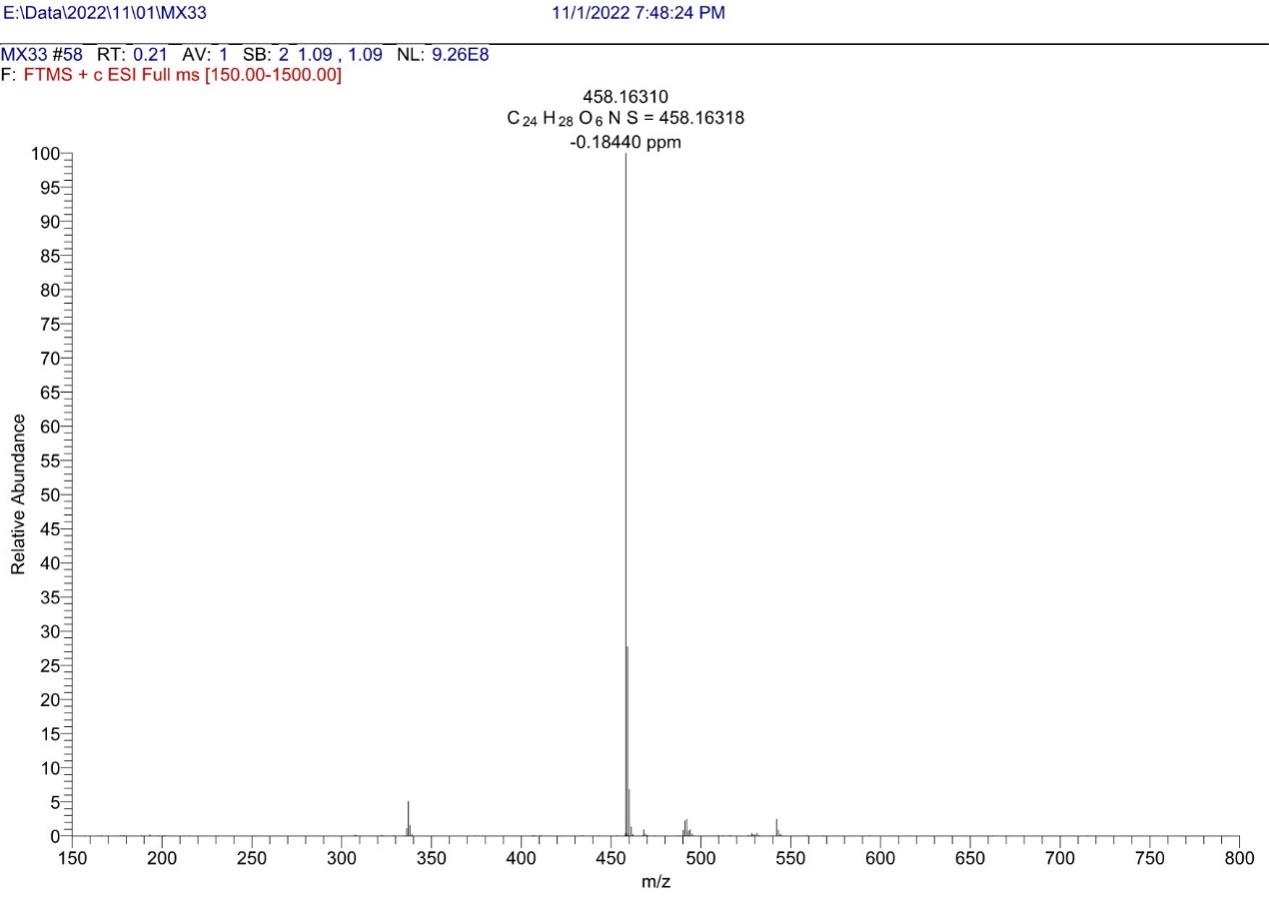


**Sources of deep learning database.**

1. ChEMBL database: 801 reported anti-*H. pylori* compounds (ChEMBL: https://www.ebi.ac.uk/chembl/): see Supplementary Excel file.
2. PubChem database: 6001, 892, and 2812 compounds with known anti-*H. pylori* activity (<https://pubchem.ncbi.nlm.nih.gov/>): see Supplementary Excel file.
3. Self-constructed 137 berberine derivatives: see Supplementary Excel file.
